# Trapping the Excess Electron of FeB-Type CeSi by Hydrogenation — Formation of Ferromagnetic Hydrides CeSiH_*x*_ (*T*_C_ ≈ 24 K)

**DOI:** 10.1021/acs.inorgchem.6c01660

**Published:** 2026-08-04

**Authors:** Leonhard Y. Dorsch, Peter C. Müller, Sarah Michels, Clemens Ritter, Anton Werwein, Oliver Janka, Jasper A. Baldauf, Rainer Pöttgen, Richard Dronskowski, Holger Kohlmann

**Affiliations:** † 9180 Leipzig University, Faculty of Chemistry, Institute of Inorganic Chemistry and Crystallography, Johannisallee 29, Leipzig 04103, Germany; ‡ Institute of Inorganic Chemistry, 9165 RWTH Aachen University, Aachen D-52056, Germany; § Institut Laue Langevin, 71 Avenue des Martyrs, BP 156, Grenoble Cedex 9 F-38042, France; ∥ Inorganic Solid State Chemistry, 9379 Saarland University, Campus C4.1, Saarbrücken 66123, Germany; ⊥ Institut für Anorganische und Analytische Chemie, 9185 Universität Münster, Corrensstrasse 30, Münster 48149, Germany

## Abstract

Two new hydrides, respectively deuterides, of the FeB-type CeSi were synthesized, and their reaction as well as magnetic behavior and structures were studied. Hydrogenation leads to two phases, a kinetic product retaining the FeB structure with hydrogen-filled tetrahedra (LaGeH type, *Pnma, P*-phase: CeSiD_0.82(2)_) and the thermodynamically preferred hydrogen-filled CrB-type structure (ZrNiH type, *Cmcm*, *C*-phase: CeSiD_0.96(1)_). Both hydrides are ferromagnets and feature Curie temperatures of 22.4(1) K (*C*-phase) and 24.3(1) K (*P*-phase), which are exceptionally high for cerium intermetallics. The effective magnetic moments of μ­(Ce^3+^) = 2.53 μ_B_ are very close to the free ion value of 2.54 μ_B_. The compounds were studied using *in situ* neutron powder diffraction and magnetic characterization and by density functional theory and chemical bonding analysis.

## Graphical abstract

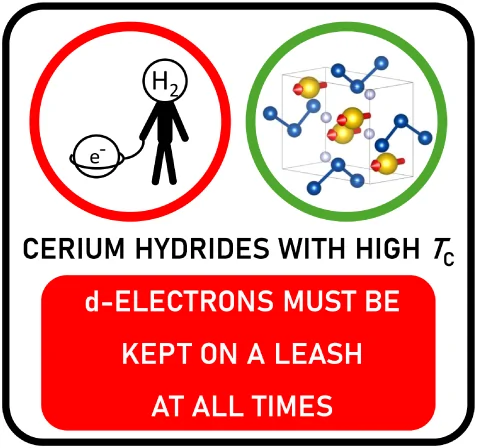

## 1 Introduction

Cerium-containing intermetallics exhibit a plethora of interesting properties, ranging from conventional (anti)­ferromagnetic ordering to intermediate and fluctuating valence, Kondo effects, and heavy Fermions.^1–6^ An extensively researched field is phases of the general composition Ce*MX*, with *M* being a transition metal and *X* a group 13, 14, or 15 element, as the mixing of the Ce­(4f) and *M*(*n*d) orbitals can lead to a wide variety of electronic states.^7–9^ Some of these compounds also take up hydrogen, opening the door for further changes in the electronic situation as well as interatomic distances.^6–9^ Generally, hydrogen uptake leads to a unit cell expansion while often retaining the overall structure. Regarding the magnetic properties of the hydrides, the geometric changes tend to overshadow the chemical effects. A good indicator is the coupling constant *J*_cf_ between the Ce­(4f) and conduction electrons. While applying pressure decreases interatomic distances and increases *J*_cf_, hydrogenation leads to an opposite effect and can therefore be described as a chemically induced negative pressure.^6,8,10^ Another closely related field is phases of the composition Ce*XX*′ which often crystallize in ThSi_2_ or AlB_2_ type structures and their coloring variants. These phases are lacking the influence of d electrons and show exclusively trivalent cerium with (anti)­ferromagnetic ordering at low temperatures.^11^

While only featuring one tetrel element, CeSi follows this behavior, as it exhibits a commensurate square wave magnetic structure below a Néel temperature of *T*_N_ = 5.6 K^12–15^ and is proposed to be a non-Kondo system.^16^ It crystallizes in the orthorhombic FeB structure type (space group *Pnma*/No. 62, Figure 1a) and features a 7 + 2 capped trigonal prismatic coordination of cerium around the silicon atoms (Figure 1g). It is further accompanied by two more silicon atoms, each centering another prism and linking up to the _∞_^1^[Si^2–^] zigzag chains in the crystallographic *a* direction (Figure 1b).^12,13^ With the exception of EuSi (CrB type), *Ln*Si with early lanthanides (*Ln* = La–Tb) crystallize in the FeB type structure, whereas the late lanthanides (*Ln* = Tm–Lu) crystallize in the CrB type. For (*Ln* = Dy–Er), a dimorphism between CrB and FeB type phases is observed.^12,13,17^

**Figure 1:**
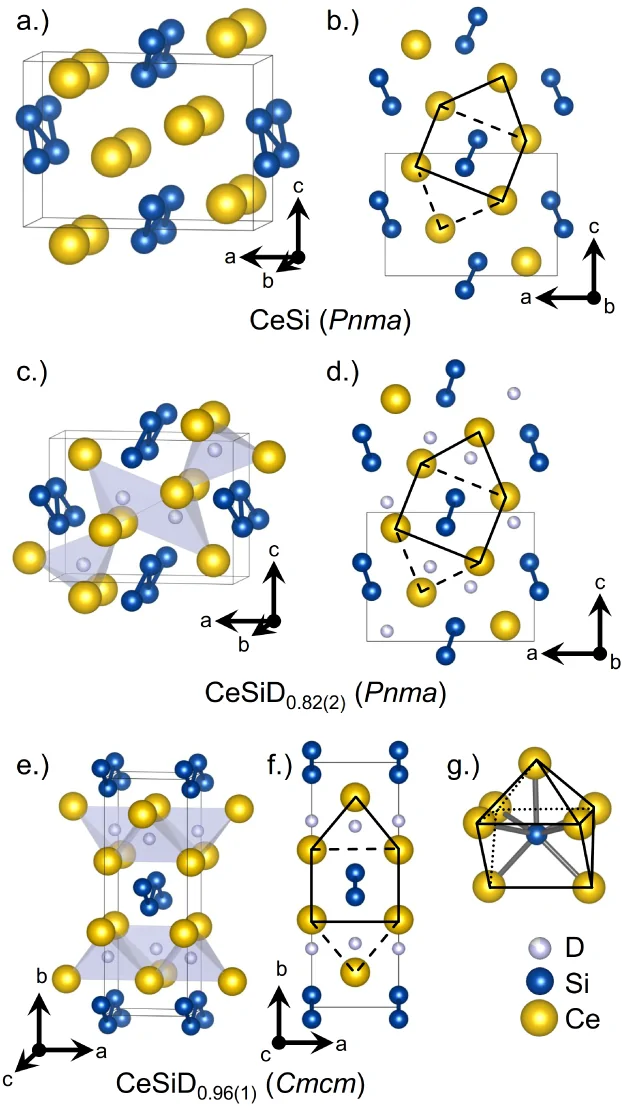
Crystal structure models of a) CeSi (FeB type), c) *P*-CeSiD_0.82(2)_ (filled FeB type), and e) *C*-CeSiD_0.96(1)_ (filled CrB type) with g) the capped trigonal prismatic coordination of Si by Ce. Projections alongside the zigzag chains show the orientation of the prisms, alternating between solid and dashed positions, in b) CeSi, d) *P*-CeSiD_0.82(2)_, and f) *C*-CeSiD_0.96(1)_.

The FeB- and CrB-type structures are closely related, as they share the same 7 + 2 capped trigonal prisms but differ in their arrangement, as the CrB-type features sheets of prisms instead of rows. This can be demonstrated by applying unit-cell twinning to a close-packed structure of *Ln* atoms and filling the resulting trigonal prismatic holes in the twin plane with silicon. For a cubic close-packed structure, this would lead to the FeB-type structure, whereas the hexagonal close-packed structure would result in the CrB type.^12,18^

In accordance with the existence of _∞_^1^[Si^2–^] zigzag chains and their intermetallic character, the phases *Ln*Si (*Ln* = La–Tb) can be formalized as *Ln* ^3+^Si^2–^e^–^.^17,19^

Previous studies on the hydrogenation (deuteration) of FeB-type phases ht-LaSi, LaGe, and NdSi resulted each time in mixtures of two hydrides (deuterides).^17,20^ The minority phase was always related to the FeB-type structure, with filled H*Ln*_4_ tetrahedra, and referred to as the LaGeH type or *P*-phase in space group *Pnma*. The majority phases were of the ZrNiH type or *C*-phase in space group *Cmcm*, a filled CrB-type structure with H*Ln*_4_ tetrahedra. The notation of the ZrNiH-type structure as the *C*-phase and the LaGeH-type structure as the *P*-phase is continued in this work as well. For the hydrogenation of CrB-type ErSi and TmSi, only the *C*-phase was reported.^21^ The hydrogenation to *Ln*SiH_*x*_ (*Ln* = La–Tb) is limited to *x* = 1 (full occupation of *Ln*_4_ voids).^17,20–22^

In these studies, hydrogen is often substituted with deuterium to accurately localize H/D positions via neutron diffraction. The isotope effects on the crystal structure are usually limited to slightly reduced lattice parameters in deuterides.

According to total energy density functional theory (DFT) calculations on the hydrides of LaSi, the *C*-phase is thermodynamically favored. Electronic structure calculations revealed a slight depopulation of the Si-π* states, leading to shorter Si–Si bonds within the chains, while the depopulation of the La-d levels leads to an incomplete metal-to-semiconductor transition during hydrogenation.^17^

This work investigated the hydrogenation behavior of CeSi, as electronic and geometric changes induced by hydrogen can be valuable tools in tuning the magnetic properties. The magnetic behavior of the hydrogenation products was studied by means of *in situ* and temperature-dependent neutron powder diffraction (NPD), magnetic measurements, and thermal analysis. The experimental results were complemented by density functional theory as well as chemical bonding analyses.

## 2 Experimental Section

### 2.1 Precursor Synthesis

As the neutron diffraction studies required multiple grams of material each, throughout all experiments and methods, around 10 g of CeSi was used. The first samples were arc-melted with a limit of ca. 500 mg per synthesis. Later samples were induction heated in tantalum ampules, allowing for samples up to 2000 mg. All samples were investigated by XRPD (*vide infra*) prior to hydrogenation (deuteration), and samples containing excessive amounts of CeSi_2_ were removed. In terms of diffraction experiments, no qualitative difference between batches from the two routes was observed.

For the arc-melted CeSi, starting materials were ingots of cerium (Smart Elements, 99.99%) and silicon lumps (Wacker, >99.9%). Larger cerium ingots were cut into smaller pieces in a glovebox and subsequently stored in Schlenk tubes under dry argon (Westfalen, 99.998%, purified over titanium sponge (870 K), silica gel, and molecular sieves). Cerium and silicon pieces were then weighed in the ideal equiatomic ratio and reacted under 800 mbar of argon in a water-cooled copper crucible of an arc melting furnace.^23^ The product buttons were turned over and remelted three times to ensure their homogeneity. The total weight losses after the arc melting procedure were never above 1%.

The induction-heated CeSi was synthesized from Ce (Smart Elements, 99.9%) and silicon (abcr, 99.9999%) lumps stored and handled under dry argon (Air Liquide, 99.999%) in a glovebox. The silicon was crushed into smaller pieces, mixed with an equimolar amount of cerium filings, and sealed in welded tantalum jackets. The tantalum was heat-treated *in vacuo* via induction heating, first to a temperature of 1275 K for 300 s and for an additional 5 s at 1975 K. The polycrystalline CeSi samples were stable in air.

### 2.2 X-ray Powder Diffraction (XRPD)

Samples of CeSi as well as hydrogenation/deuteration products were studied on a Guinier geometry G670 camera (HUBER Diffraktionstechnik, Rimsting, Germany) with an image-plate detection system and Cu–K_α1_ radiation. Flat samples were fixed in Lithelen or Apiezon grease between Kapton foils under an inert atmosphere.

### 2.3 Hydrogenation/Deuteration in Autoclaves

Hydrogenation reactions were carried out in autoclaves made from Nicofer 5219Nb alloy containing either H_2_ (99.9%, Air Liquide) or D_2_ (Air Liquide, 99.8% isotope purity). For the synthesis of CeSiH_*x*_ an autoclave was put under 6 MPa of H_2_ pressure and heated to 775 K for 48 h. For the deuteration, the autoclave was filled to 8 MPa of D_2_ pressure and heated to 775 K for 24 h. The hydrides and deuterides were obtained as gray, brittle powders.

### 2.4 Neutron Powder Diffraction (NPD)

Neutron powder diffraction measurements on CeSiD_*x*_ were performed at D2b, *λ* = 1.594 Å (Institut Laue-Langevin, Grenoble, France).^24^ Data was collected for about 8.5 h per measurement in thin-walled vanadium cylinders with an inner diameter of 5 mm, sealed with indium wire. All measurements below ambient temperature were taken in an orange cryostat (ILL, Grenoble). The data of experiment 5-24-573 is presented with the ILL internal raw data NUMOR labeling. The raw data is available at ref 25. The data further includes measurements on a second CeSiD_*x*_ sample at 1.6 K (see S14 pp. in the Supporting Information).

**Temperature-dependent NPD at low temperatures** on CeSiD_*x*_ was performed at the high-intensity two-axis diffractometer D20 (Institut Laue-Langevin, Grenoble, France)^26^ in indium-sealed 5 mm vanadium cylinders cooled with an orange cryostat (ILL, Grenoble). The wavelength of *λ* = 2.4166(2) Å was refined using the sample as a reference with structural parameters taken from D2b data (*vide supra*). The experiment started at 10 K with a time resolution of 10 min per measurement and a heating rate of 0.03 K/min from 10 to 35 K and a rate of 0.12 K/min from 35 to 50 K. The raw data of this experiment (5-24-573) is available with the ILL internal raw data NUMOR labeling at ref 27.

Additionally, an ***in situ*** **neutron powder diffraction experiment** on the deuteration of CeSi was carried out at diffractometer D20 (ILL, Grenoble, France).^26^ The sample environment was a single-crystal sapphire cell with an inner diameter of 6 mm, connected to a gas supply system with D_2_ gas (99.8% isotope purity, Air Liquide, Paris, France) and heated via two laser beams as described in ref 28. The sample preparation as well as the connection to the gas supply system was performed under an inert atmosphere, allowing only Ar and D_2_ gas to be present during the reaction. Prior to the experiment, the wavelength of *λ* = 1.8685(3) Å was refined using a Si NIST 640d sample in the same cell as a reference. As a first step, the pristine CeSi was measured for 10 min, whereas during the experiment, diffraction patterns were obtained with a time resolution of 2 min, each represented in a single NUMOR within the data set of experiment 5-24-720 available at ref 29.

### 2.5 Rietveld Refinements

Refinements were performed with the Rietveld method on the obtained XRPD and NPD data sets using the FullProf Suite (ver. 5.10),^30^ including the BasIreps program and trial and error to determine the orientation of the magnetic moments. The data were visualized using Gnuplot 6.0 ^31^ and Origin 2019 ^32^ as well as VESTA.^33^

### 2.6 Magnetic Properties Characterization

The precursor CeSi, the CeSiH_*x*_ and the CeSiD_*x*_ samples were ground to fine powders in a glovebox and filled into polypropylene capsules. These were attached to a brass sample holder rod before being investigated using the VSM (vibrating sample magnetometer) option of a Physical Property Measurement System (PPMS2) or a Dynacool Physical Property Measurement System by Quantum Design. The magnetization *M*(*H*, *T*) was measured in the temperature range of 3.5–305 K or 2.5–305 K using fields up to 80 kOe or 90 kOe (depending on the machine). Fitting and plotting the data was done with OriginPro 2024,^34^ and the graphical editing with the program CorelDraw 2017.^35^

### 2.7 Scanning Electron Microscopy (SEM) and Energy Dispersive X-ray (EDX) Spectroscopy

CeSiD_*x*_ was investigated via SEM-EDX in an LEO 1530 (Carl Zeiss AG) with a voltage of 20 kV using a liquid nitrogen-cooled EDX detector (Oxford Instruments). The ground sample was pressed into a pellet under argon, sputtered with carbon, and quickly transferred into the microscope.

### 2.8 Differential Scanning Calorimetry (DSC)

The samples were prepared with ca. 20 mg of CeSi in 40 μL aluminum crucibles that were crimped in a glovebox under an Ar atmosphere. Measurements were performed in a TA Instruments Q1000 DSC under a hydrogen pressure of 2.4 MPa.

### 2.9 Computational Details

All structures were optimized using the Vienna ab initio Simulation Package (VASP),^36–39^ using projector-augmented wave (PAW)^40^ pseudopotentials (Ce_3_, Si, and H) and a kinetic-energy cutoff of 500 eV. The generalized gradient approximation with the PBEsol^41^ functional was used to model exchange and correlation. The convergence criterion of the electronic optimization was set to 10^–6^ eV, whereas structures were considered optimized when Hellmann–Feynman forces fell below 5·10^–3^ eV Å^–1^. Van der Waals forces were accounted for with Grimme’s D3 correction using Becke–Johnson damping.^42,43^ The Brillouin zones were constructed using Γ-centered Monkhorst–Pack grids^53^ with 15 × 5 × 15 (*C*-CeSiH), 7 × 15 × 9 (*P*-CeSiH), 7 × 15 × 11 (CeSi), and 3 × 3 × 3 (H_2_) k-points, respectively. Molecular hydrogen was modeled as an isolated molecule in a cubic box with lattice parameters of 20 Å. Brillouin-zone integration was done using a Gaussian-smearing width of 0.05 eV for the structural optimization. For the optimized structures, single-point calculations were performed with Blöchl’s tetrahedron^44^ integration, and the resulting wave functions were projected onto a local-orbital basis using the Local-Orbital Basis Suite Toward Electronic-Structure Reconstruction (LOBSTER) program.^45^ With this unitary basis transformation, local densities of state, Löwdin-style populations^46^ as well as the crystal orbital bond index (COBI)^47^ are conveniently determined to quantify chemical bonding. In a consecutive basis-set transformation, we used the Linear Combination of Fragment Orbitals (LCFO) method^48^ to convert the atomic-orbital basis into a fragment-orbital basis in order to examine the frontier orbitals of the Si–Si bonds. Figures were created using wxDragon^49^ and VESTA^33^ and postprocessed using CorelDRAW 2024.^35^

### 2.10 Safety Statement

Caution! Hydrogen is classified as a GHS flammable gas, category 1. Hydrogen gas was only handled in closed systems made of hydrogen-resistant alloys.

## 3 Results and Discussion

### 3.1 Crystal Structures

CeSi was obtained as brittle lumps and was easily ground into gray metallic powder. The lattice parameters of *a* = 8.3014(1) Å, *b* = 3.96559(5) Å, *c* = 5.96533(7) Å (Table 1) match well with previous reports.^12,13^ The samples contained small amounts of CeSi_2_ (< 3 wt %, Figure S1) that remained unchanged upon hydrogenation and did not show in neutron experiments with *ex situ* deuterated samples.

**Table 1:** Crystal Structure Data of CeSi and Selected Interatomic Distances, Determined by Neutron Powder Diffraction at Room Temperature (Figure S1), NUMOR: 244361 ^29^

CeSi (*Pnma*): *a* = 8.3014(1) Å, *b* = 3.96559(5) Å, *c* = 5.96533(7) Å, *V* = 196.379(4) Å^3^						
	Wyckoff site	*x*	*y*	*z*	*B*_iso_/Å^2^	sof
Ce	4*c*	0.1797(1)	1/4	0.1166(3)	0.59(3)	1
Si	4*c*	0.0340(2)	1/4	0.6167(4)	0.62(3)	1
Ce–Ce	3.767(2) Å/3.843(2) Å/3.96560(6) Å/4.445(2) Å					
Si–Si	2.488(2) Å					

The NPD data (Figure 2) of deuterated CeSi revealed the presence of two deuterides, similar in structure to those found in hydrogenation (deuteration) products of other FeB-type *LnTt* (*Ln* = La, Nd; *Tt* = Si, Ge, Sn) phases.^17^ *P*-CeSiD_0.82(2)_ or “*P*-phase” (LaGeH type, Table 2, Figure 1c) in space group *Pnma* retains the FeB-type structure of CeSi (Figure 1a), with the quasi-tetrahedral Ce_4_ voids being partially filled with deuterium (hydrogen). Compared to CeSi, it shows an increase in the unit cell volume of 3.6%. It displays a similar anisotropic behavior regarding the change of lattice parameters, as *P*-LaSiH_*x*_ and *P*-NdSiH_*x*_ with a decrease in *a* to 8.0860(5) Å, a relatively unchanged *b* of 4.0067(3) Å, and an increase in *c* to 6.2801(5) Å.^17^ The second phase, *C*-CeSiD_0.96(1)_ or “*C*-phase” (ZrNiH type, Table 3, Figure 1e) in space group *Cmcm*, is based on the CrB structure type with partially deuterium-filled Ce_4_ voids (*a* = 4.2414(2) Å, *b* = 12.0765(4) Å, *c* = 3.9927(2) Å). It features a higher deuterium content and a slightly larger volume increase compared to CeSi of 4.1%. The same was the case for *C*-LaSiH_*x*_ and *C*-NdSiH_*x*_.^17^ In accordance with the lanthanide contraction, the unit cell volumes of both *P*-CeSiD_0.82(2)_ and *C*-CeSiD_0.96(1)_ slot in between those of the hydrides (deuterides) of LaSi and NdSi.^17^ Crystal structure parameters of the respective hydrides (*C*-CeSiH_*x*_ and *P*-CeSiH_*x*_) can be found in the Supporting Information (see Figure S2, Tables S1, S2).

**Figure 2:**
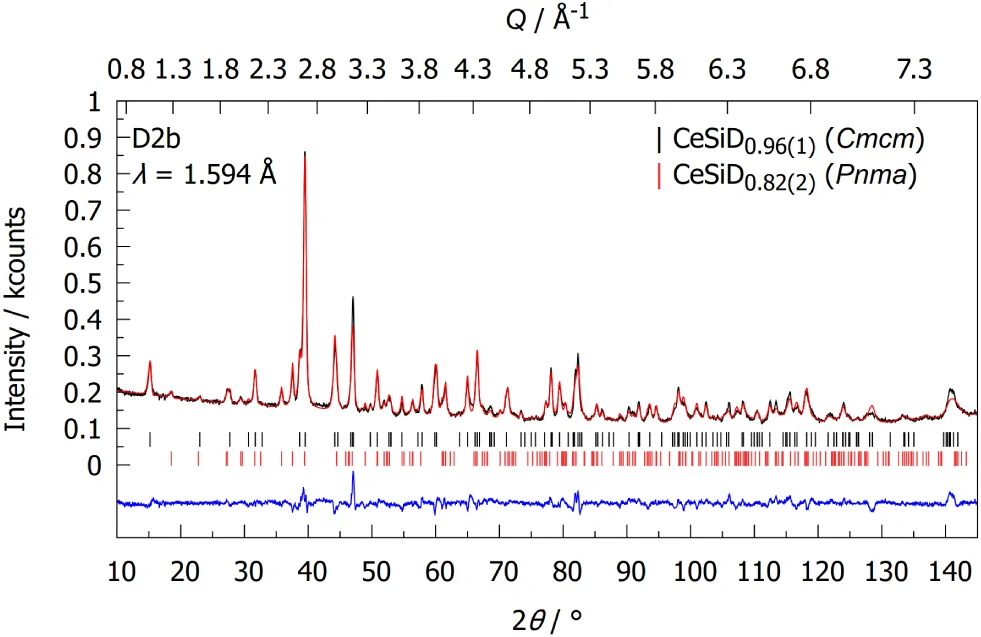
Rietveld refinement of the crystal structures of CeSiD*_x_* based on the NPD pattern at room temperature, *C*-CeSiD_0.96(1)_: 64(1) w.-%, *R*_Bragg_ = 6.51%; *P*-CeSiD_0.82(2)_: 36(1) w.-%, *R*_Bragg_ = 8.35%, taken at D2b (Grenoble, France) at *λ* = 1.594 Å, *R*_p_ = 3.16%, *R*_wp_ = 4.32%, *χ* ^2^ = 5.52, NUMOR: 588195–588212.^25^

**Table 2:** Crystal Structure of *P*-CeSiD_0.82(2)_ and Selected Interatomic Distances Determined by Neutron Powder Diffraction at Room Temperature as Refined from Sample A (Figure 2), NUMOR: 588195–588212 ^25^

CeSiD_0.82(2)_ (*Pnma*): *a* = 8.0860(5) Å, *b* = 4.0067(3) Å, *c* = 6.2801(5) Å, *V* = 203.46(3) Å^3^						
	Wyckoff site	*x*	*y*	*z*	*B*_iso_/Å^2^	sof
Ce	4*c*	0.173(1)	1/4	0.121(2)	0.76(8)	1
Si	4*c*	0.029(2)	1/4	0.612(2)	0.32(7)	1
D	4*c*	0.379(1)	1/4	0.422(2)	1.61(7)	0.82(2)
Ce–Ce		3.76(1) Å/3.93(1) Å/4.0067(3) Å/4.356(1) Å				
Ce–D		2.39(1) Å/2.398(7) Å/2.52(1) Å				
Si–Si		2.494(9) Å				

**Table 3:** Crystal Structure Data of *C*-CeSiD_0.96(1)_ and Selected Interatomic Distances Determined by Neutron Powder Diffraction at Room Temperature as Refined from Sample A (Figure 2), NUMOR: 588195–588212 ^25^

CeSiD_0.96(1)_ (*Cmcm*): *a* = 4.2414(2) Å, *b* = 12.0765(4) Å, *c* = 3.9927(2) Å, *V* = 204.51(1) Å^3^						
	Wyckoff site	*x*	*y*	*z*	*B*_iso_/Å^2^	sof
Ce	4*c*	0	0.3575(3)	1/4	*B*_*iso*_(Ce, *P*-CeSiD_0.82_)	1
Si	4*c*	0	0.0490(4)	1/4	*B*_*iso*_(Si, *P*-CeSiD_0.82_)	1
D	4*c*	0	0.7607(3)	1/4	*B*_*iso*_(D, *P*-CeSiD_0.82_)	0.956(8)
Ce–Ce	3.902(3) Å/3.979(4) Å/3.9927(2) Å/4.2414(2) Å					
Ce–D	2.422(2) Å/2.454(3) Å					
Si–Si	2.321(3) Å					

When comparing the hydrogenation products to their precursors, one point of interest is the Si–Si distance. According to DFT calculations on the hydrogenation of LaSi, hydrogenation does not only depopulate the La-d but also the Si-π* states, leading to a shortening of the Si–Si bonds in the hydrides.^17^ This was reaffirmed for LaSi as well as CeSi and its hydrides in calculations in this study (*vide infra*). In the relaxed structures, the Si–Si distances decrease from the precursor to the *P*-phase and further to the *C*-phase.

As the deuterium content rises from CeSi to *P*-CeSiD_0.82_ and *C*-CeSiD_0.96_ one would expect the experimentally determined Si–Si bonds to decrease accordingly. This is, however, not observed, as the bond length within the Si–Si chains does not change significantly from pristine CeSi (2.488(2) Å) to the *P-*CeSiD_0.82(2)_ (2.494(9) Å) but decreases substantially in the *C*-CeSiD_0.96(1)_ (2.321(3) Å). In the crystal structure refinements (Figure 2), a second minimum with only slightly larger *R* values was found. The deuterium contents and Si–Si distances change here to *P-*CeSiD_0.86(2)_ (2.456(9) Å) and *C*-CeSiD_0.84(1)_ (2.489(3) Å). The two minima can be simplified as minimum A: *d*(Si–Si, *C*-CeSiD_*x*_) ≪ *d*(Si–Si, *P*-CeSiD_*x*_) ≈ *d*(Si–Si, CeSi) and minimum B: *d*(Si–Si, *P*-CeSiD_*x*_) < *d*(Si–Si, *C*-CeSiD_*x*_) < *d*(Si–Si, CeSi). Both are partially consistent with the theoretical calculations that suggest *d*(Si–Si, *C*-CeSiD_*x*_) < *d*(Si–Si, *P*-CeSiD_*x*_) < *d*(Si–Si, CeSi). Taking a broader look at the various diffraction patterns presented in this study (and some additional ones), all refinements on measurements above ambient temperature converged solely in minimum A, while those below ambient resulted clearly in minimum B. At room temperature, both A and B describe the experimental data almost equally well. Table S13 at the end of the Supporting Information contains a summary of the various structural parameters gained from the refinements in this work. This might hint at a temperature dependence of the Si–Si distances possibly caused by the anisotropic thermal expansion. However, this interpretation requires further investigation for confirmation. A comparison with room temperature LaSi/LaSiD_*x*_ from Werwein et al. is difficult, as the *P*-phase in question was taken as is from the theoretical calculation.^17^ The also comparable NdSi/NdSiD_*x*_ does show the expected behavior even though no DFT calculations were presented. The pair LaGe/LaGeD_*x*_ seems to fall into minimum B.^17^

Several factors have to be considered in addition. First, at best there is a roughly 70/30% split between *C*- and *P*-CeSiD_*x*_, and diffraction data suffer from a considerable reflection overlap. Furthermore, the Rietveld refinement (Figure 2) on the neutron diffraction data shows some intensity issues, most notably at *Q* = 3.14 Å^–1^ coinciding with the 2 2 0 and 0 0 2 reflections of the *C*-phase and the 0 2 0 reflection of the *P*-phase. Similar issues were observed in refinements of other *LnTt*H_*x*_ hydrides (*Ln* = La, Nd; *Tt* = Si, Ge, Sn).^17^ The discrepancies might stem from the layered nature of the crystals as visible in the SEM images taken of CeSiD_*x*_ with a secondary electron detector (Figure S3a), thus leading to anisotropic line broadening for both X-ray and neutron powder diffraction. Intensity issues in Rietveld refinements caused by such delaminated crystal layers were previously described for hydrides of CrB-type alkaline earth silicides.^50,51^ The hydrogenation (deuteration) of CeSi always gave a combination of both *P*- and *C*-type hydrides (deuterides) so far. The synthesis of phase-pure *P*- and *C*-phases might therefore help with the differentiation of the intensity issues and subsequently allow for more reliable structural and temperature-dependent data. Because of these issues, we only tentatively interpret the crystal structures to show a temperature-dependent progress of Si–Si distances with a switch from one state (minimum A) with *d*(Si–Si, *C*-CeSiD_*x*_) ≪ *d*(Si–Si, *P*-CeSiD_*x*_) ≈ *d*(Si–Si, CeSi) at high temperature to another state (minimum B) with *d*(Si–Si, *P*-CeSiD_*x*_) < *d*(Si–Si, *C*-CeSiD_*x*_) < *d*(Si–Si, CeSi) upon lowering the temperature.

The homogeneity of the sample was confirmed by SEM with the backscatter electron detector at 20 μm (Figure S3b). Ce and Si were also evenly distributed in EDX mappings, with an analysis of 21 points on a scale of 100 μm giving a slight excess of Ce (52.0(3) at%) over Si (48.0(3) at%) (Figure 3). As the pellet was not polished, this deviation from a 1:1 ratio seems plausible.

**Figure 3:**
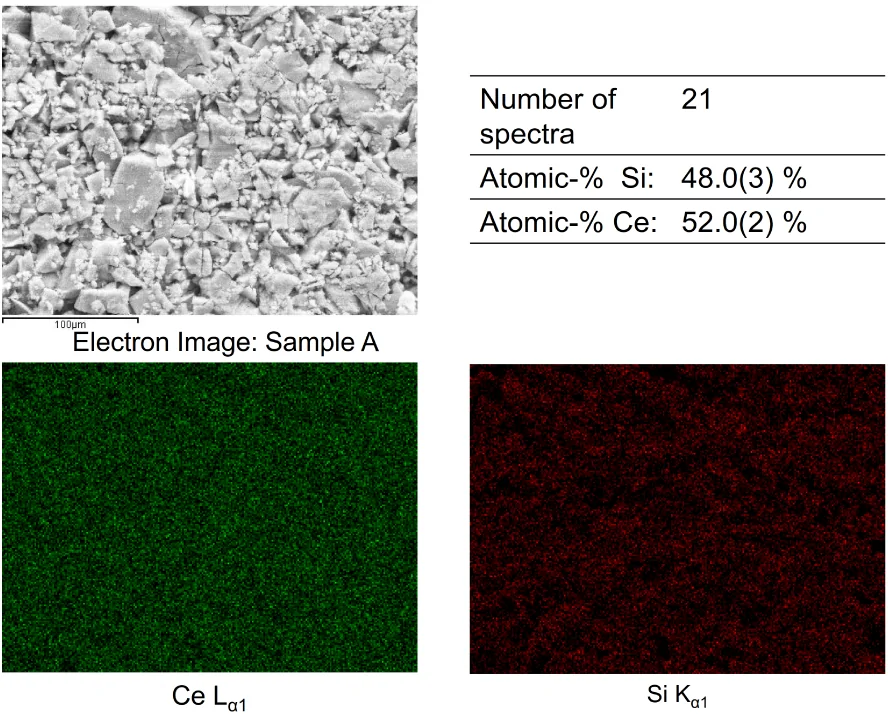
Scanning electron microscope image of a pressed pellet of Sample A (scale: 100 μm) and the averaged result of the 21 EDX spectra; values in brackets give the propagated standard deviation derived from individual measurements. Bottom: distribution of Ce and Si through the shown surface.

### 3.2 Hydrogenation Behavior

While the *P*-phase can be described as hydrogen-filled CeSi, the CrB-based *C*-phase is only related to its precursor through the building blocks in the form of the capped trigonal 7 + 2 coordinated prisms (see Figure 1d,f,g). A former study, therefore, assumed that the *P*-phase is formed first, with the thermodynamically favored *C*-phase emerging at higher temperatures.^17^ To confirm this hypothesis, an *in situ* neutron powder diffraction experiment on the deuteration of CeSi was performed. The experiment consisted of two steps; for the full experiment, see Figure S4. First, CeSi was kept in an atmosphere of 2.0 MPa D_2_ pressure, heated up to 713 K, and then cooled again (Figure 4). For each measurement of 2 min, an individual refinement was performed. The lattice parameters of CeSi showed a linear rise alongside the temperature, showing no signs of a reaction around 440 K. From there on, CeSi was consumed to form the *P*-phase of CeSiD_*x*_, shortly followed by the *C*-phase from 540 K. Higher temperatures led to an increase in the *C*-phase fraction. The patterns from 100 min/710 K onward no longer contained reflections of the educt (Figure 5). The deuterium positions were kept fully occupied for both phases to stabilize the Rietveld refinements. In accordance with the room temperature measurement, both phases show a volumetric increase compared to CeSi, with the *C*-phase being slightly larger than the *P*-phase (Figure 6). Between CeSi and *P*-CeSiD_*x*_, the anisotropy regarding the lattice parameters can be observed again (*vide supra*), with a decrease in *a,* a relatively unchanged *b* and a rise in *c* (Figure S5). The Si–Si distances are again almost equal between CeSi and *P*-CeSiD and lower for *C*-CeSiD (Figure S6). For both deuterides, the Si–Si distances increase with the temperature while intrachain angles decrease (Figure S7). The opposite is observed for CeSi. Throughout the whole reaction, the Si–Si distances in *C*-CeSiD remain shorter and the intrachain angles closer to 120° than those of *P*-CeSiD, hinting at a higher Si–Si bond order. The phase contents of roughly 70% *C*- to 30% *P*-phase were reached at this state and did slightly revert upon cooling.

**Figure 4:**
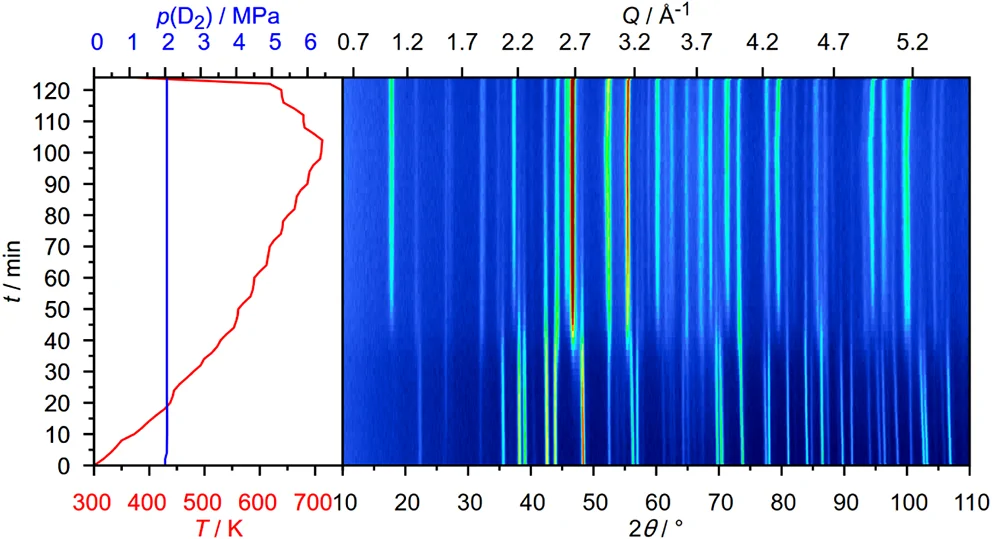
False color plot of the *in situ* deuteration experiment of CeSi (up to 124 min; see Figure S5 for the full experiment); D20 (ILL), *λ* = 1.8685(3) Å, NUMOR: 244362–244424.^29^

**Figure 5:**
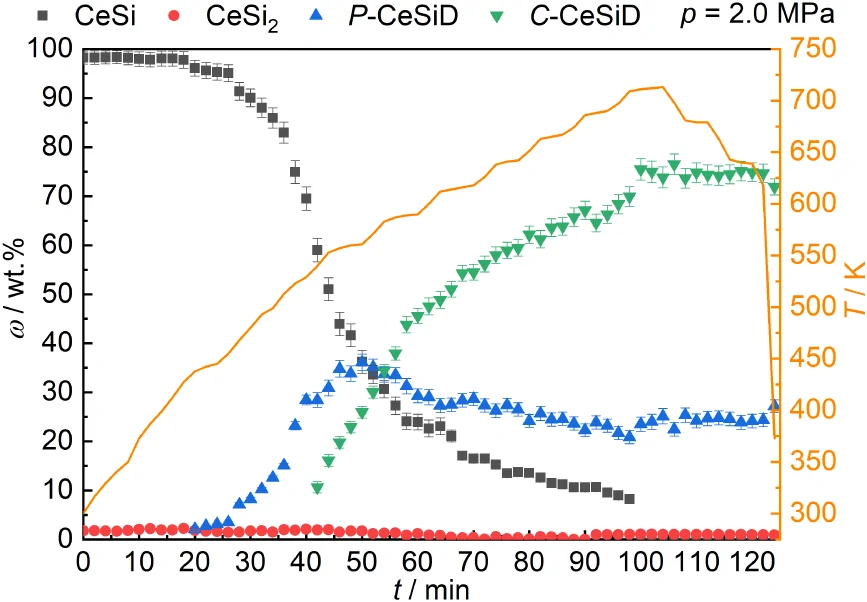
Refined phase fractions from the *in situ* deuteration experiment on CeSi; errors are given as ±*σ* or smaller than the symbols.

**Figure 6:**
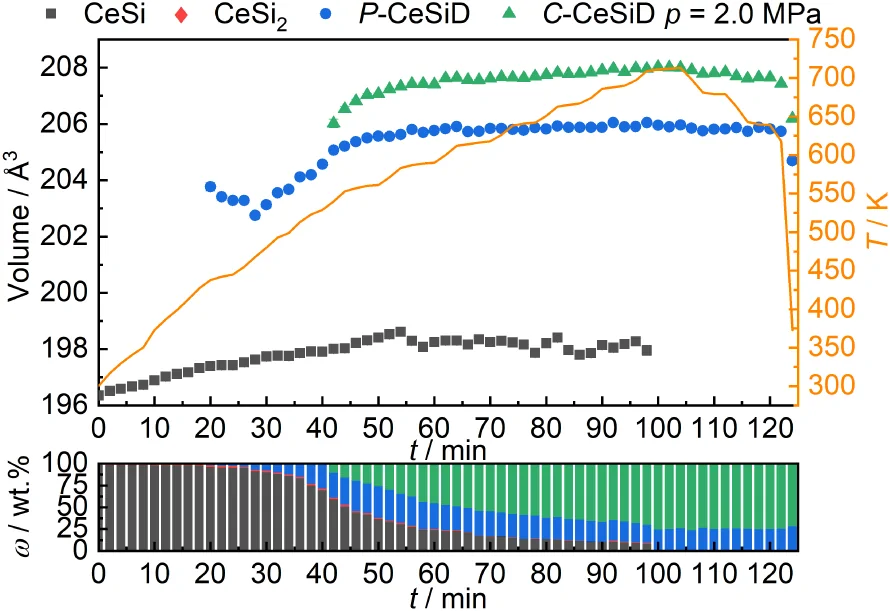
Refined unit cell volumes of CeSi, *P*-CeSiD, and *C*-CeSiD from the *in situ* deuteration experiment on CeSi, with the lower part showing the phase fractions at corresponding times; errors are given as ±*σ* or smaller than the symbols.

In the second step of the experiment, the deuteride was kept at a temperature of 500 K, and the pressure gradually increased to 6.45 MPa. A sequential refinement (Figure S8, Tables S3 and S4) showed no changes regarding the mass fraction (Figure S9) or lattice parameters (Figures S10 and S11). The refined deuterium contents (Figure S12) did not deviate significantly from full occupation; however, higher values were reached for the *C*-CeSiD_*x*_. This is in agreement with the higher deuterium contents of the *C*-phase observed in CeSiD_*x*_ (*vide supra*) as well as the *C*-phase of LaSiH_*x*_.^17^ The last measurement was taken at a pressure of 6.27 MPa and 353 K. The refinement can be found in the Supporting Information (Figure S13, Tables S5 and S6).

The *in situ* NPD experiment, therefore, confirms that the *P*-phase is formed first, with the *C*-phase following at higher temperatures. The ratio between the *C*- and *P*-phases is rather temperature-dependent than hydrogen (deuterium) pressure-dependent. As the formation of the *P*- and *C*-phases was differentiable by the *in situ* NPD experiment, one might expect two distinct signals in thermal analysis as well. This was, for example, the case for the hydrogenation of NdGe.^17^ However, a DSC measurement under 2.4 MPa of hydrogen pressure did only show one clear signal, only allowing the confirmation of the exothermic nature of the hydrogenation (Figure S13). Changes in heat flow were only observed in the first heating phase, with none registered during cooling or a second cycle.

### 3.3 Stability and Bonding Analysis

As a complement to the experimental observations discussed before, we conducted density-functional theory and chemical-bonding analyses. First, we determined the respective formation energies of *C*-CeSiH and *P*-CeSiH according to the experimental reaction equation:

1 $$CeSi+\frac{1}{2}H_{2}\to CeSiH$$

In accordance with the experimental observation, both phases of CeSiH are formed in an exothermic hydrogenation with a reaction enthalpy of −66.4 kJ mol^–1^ for *C*-CeSiH and −61.3 kJ mol^–1^ for *P*-CeSiH. The formation of *C*-CeSiH is slightly favored by 5.1 kJ mol^–1^. The stability of both compounds can be rationalized by the respective energy–volume curves and the Birch–Murnaghan fits shown in Figure 7. From this perspective, it is clear that *C*-CeSiH is thermodynamically preferred over *P*-CeSiH as the former possesses a lower energy over the whole range examined here. However, the difference between both curves is relatively low, with a shift of about 5 kJ mol^–1^ that can easily be attributed to the strong structural similarity between both *C*- and *P*-CeSiH.

**Figure 7:**
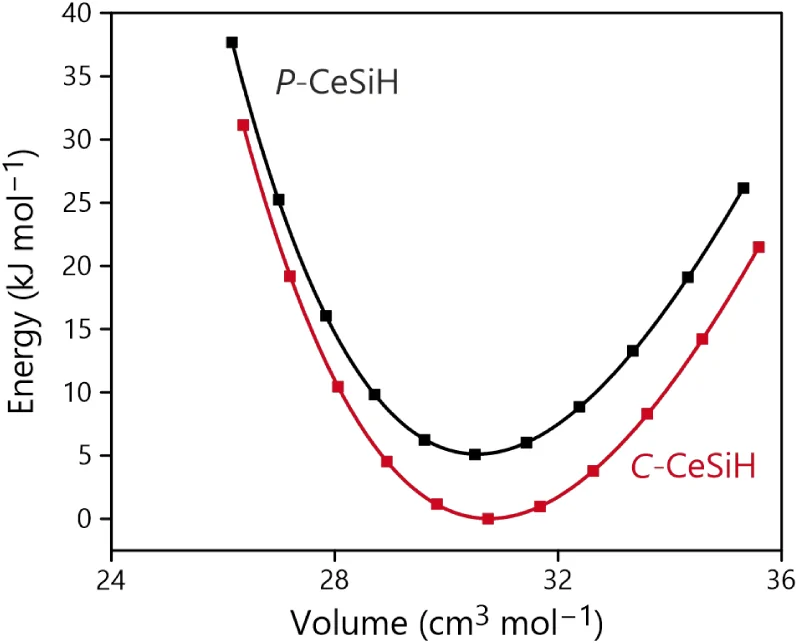
Energy–volume curve and Birch–Murnaghan fit of *C*-CeSiH (red curve) and *P*-CeSiH (black curve). The fit parameters are summarized in Table S7.

As already alluded to in the introduction, hydrogenation of rare-earth tetrel compounds oxidizes the rare-earth metal, and this is also found for CeSiH, as revealed by the Löwdin charges in Table 4 and local DOS in Figure 8. In the educt CeSi, the charges of both Ce and Si (±0.33) are well below the Zintl–Klemm–Busmann limit of ±2. This large deviation is caused by the metallic character of CeSi, and it was also found for other metallic silicides such as Ba_3_Si_4_.^48^ Larger charges would indicate higher electron localization, meaning hindered electric conductivity. From the projected DOS, it is clear that both 6s and 5d orbitals of Ce are depopulated compared to the CeSiH phases. The valence orbitals of silicon are also slightly depopulated, which leads to a lowered Löwdin charge from −0.33 to −0.26.

**Table 4:** Löwdin Charges of CeSi, *P*-CeSiH, and *C*-CeSiH

	CeSi	*P*-CeSiH	*C*-CeSiH
Ce	+0.33	+0.46	+0.47
Si	–0.33	–0.26	–0.26
H		–0.20	–0.21

**Figure 8:**
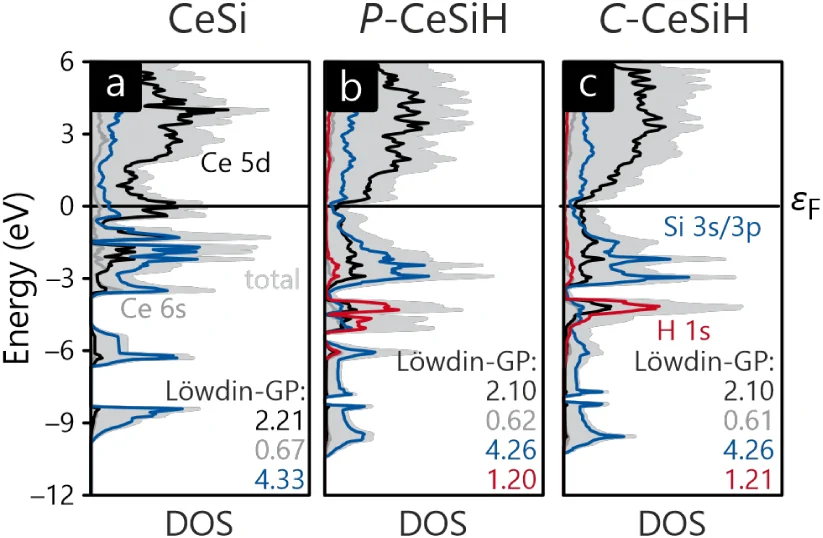
Local density of states (DOS) of a) CeSi, b) *P*-CeSiH, and c) *C*-CeSiH. Cumulative Löwdin gross populations are given for Ce-5d, Ce-6s, Si-3*s*/3p, and H-1s orbitals.

Since the energetic differences between the *P*- and *C*-phases of CeSiH do not show in the DOS and charges, it is only natural to assume that its origin is not in the ionic part of the lattice energy but can rather be attributed to *covalent* contributions. For these analyses, we employ the Crystal Orbital Bond Index (COBI)^47^ that is the generalization of the molecular Wiberg/Mayer bond index to the periodic solid.^52,53^ As such, the integrated COBI (ICOBI) equals the bond order of a certain atom–atom pair. Figure 9 shows the COBI plots for a Si–Si bond in CeSi as well as *P*- and *C*-CeSiH. From this consideration, the bond order increases from CeSi (0.62) to *P*-CeSiH (0.70) and reaches its maximum value for *C*-CeSiH (0.72). This course was already inferred in the respective bond lengths: the Si–Si distance is largest for CeSi and shortest for *C*-CeSiH, both for the experimentally observed as well as DFT-optimized structures. Interestingly, all three compounds have a σ- and π-bonding contribution that are similar in their magnitude. The respective π-part (red curves in Figure 9) is about the same in all three cases. Their difference can be found in the σ-part (blue lines). Here, *C*-CeSiH has the largest value, meaning the σ-part of the Si–Si bond is stronger compared to *P*-CeSiH and CeSi. Since all other bonds, i.e., Ce–Ce, Ce–Si, Ce–H, Si–H, and H–H, do not differ significantly between *C*-CeSiH and *P*-CeSiH, we can conclude that the structural preference is caused by the Si–Si bond and the σ-bonding contribution in particular.

**Figure 9:**
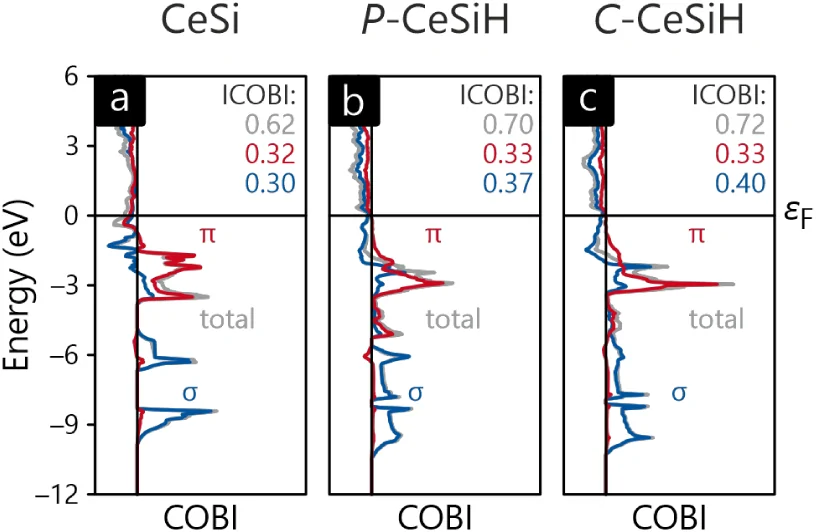
Crystal orbital bond index (COBI) of the Si–Si bonds in a) CeSi, b) *P*-CeSiH, and c) *C*-CeSiH. The total COBI is separated into σ- and π-contributions according to the orbital symmetry of the rotated basis functions.

The bonding situation of the Si–Si bond is not only visible in COBI, but it is also apparent in the respective frontier orbitals of this bond. The Linear Combination of Fragment Orbitals technique can construct these orbitals from a given atomic-orbital basis. Thereby, the Molecular Orbital Formation Energy (MOFE) falls off as a generalization of the Crystal Orbital Hamilton Population (COHP).^54^ In contrast to COHP, MOFE decomposes the band energy into atomic- or molecular-orbital contributions that arise when atomic orbitals combine into molecular or fragment orbitals. The MOFE as well as the IMOFE in Figure 10a and c further reveal the π-contribution inside the Si–Si bond that is formed by e_1u_ and e_1g_ orbitals. As already found in COBI, the total IMOFE of the π-bonding e_1u_ and antibonding e_1g_ is about −1.0 eV for both *C*- and *P*-CeSiH. The difference between both fragments is again in the σ-bonding 1a_1g_ orbital that is the lowest-lying valence orbital. Interestingly, the highest occupied molecular orbital (HOMO) is the 2a_1g_ that is occupied by about 1.2 electrons but has an IMOFE of just +0.3 eV. Such minimal energetic influence with simultaneous significant orbital occupation is characteristic of nonbonding contributions, and, as such, this 2a_1g_ orbital can clearly be identified as having lone-pair character.

**Figure 10:**
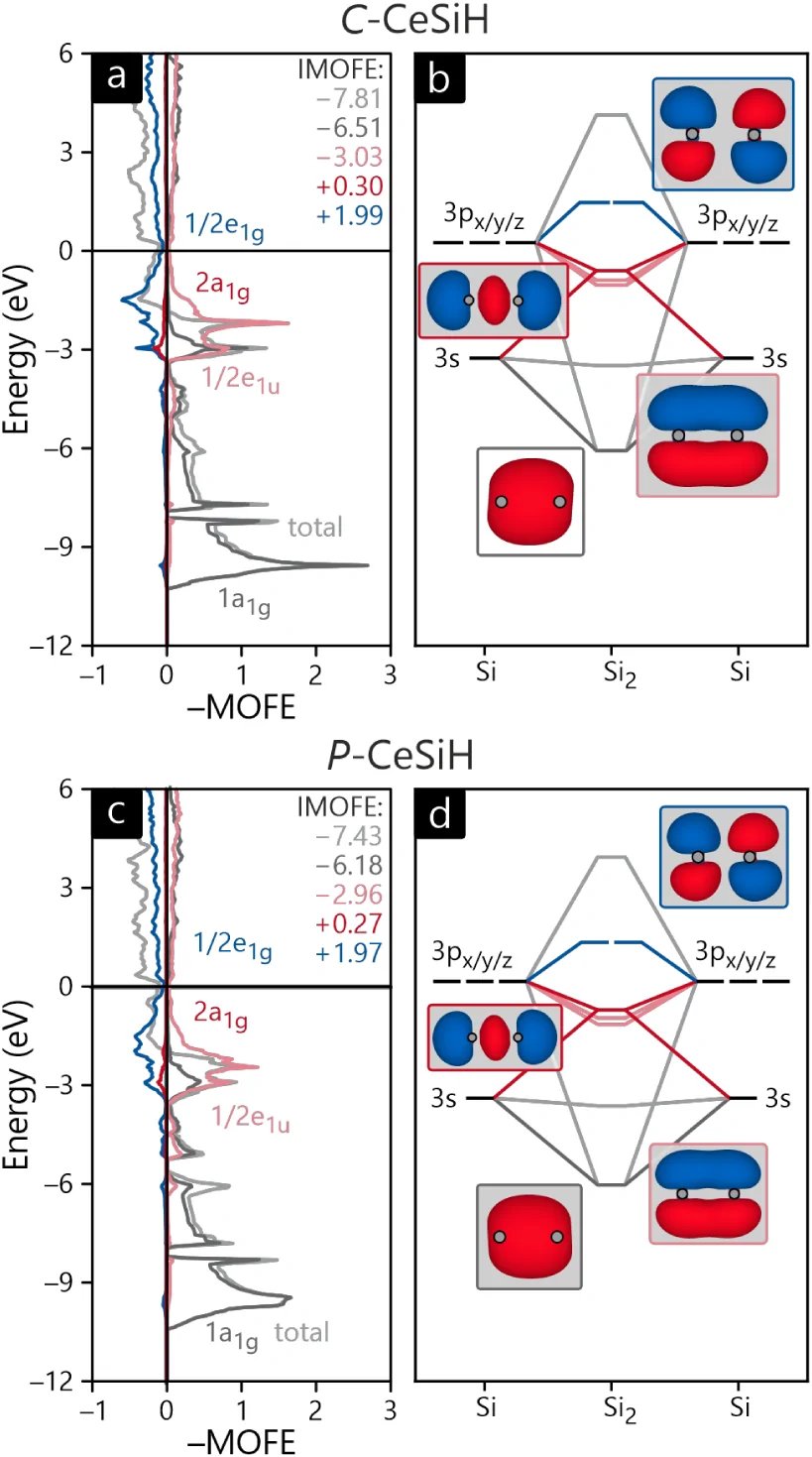
Molecular orbital formation energy (a, c) and molecular orbital diagrams (b, d) of the Si–Si bond in *C*-CeSiH (a, b) and *P*-CeSiH (c, d).

From the theoretical observations above, it is now safe to conclude that both phases of CeSiH are very close, with a slight thermodynamic preference to *C*-CeSiH, while *P*-CeSiH can be identified as the kinetic product in the hydrogenation of CeSi. This conclusion is in perfect agreement with the experimental results that first show the formation of *P*-CeSiH that later transforms into *C*-CeSiH. Yet, this conversion is not complete and likely has a low activation barrier, leading to an equilibrium with both phases existing simultaneously.

#### 3.3.1 Comparison with La-Based Compounds

Since LaSi shows the same hydrogenation behavior, we will conclude the theoretical analyses by a comparison between the isostructural Ce- and La-based compounds.

Figure 11a shows the relation between the bond order (ICOBI) and interatomic distance for CeSi and LaSi as well as their hydrides and elemental silicon. Both ICOBI and bond length of the Si–Si bonds are very close among the isostructural phases. All hydrides have an ICOBI around 0.7, whereas CeSi as well as LaSi are about 0.6. The interatomic distances of the Ce-containing compounds are slightly shorter, a consequence of the lanthanide contraction. This shortening is accompanied by an increased bond strength as measured by ICOBI. In summary, the relation of bond length–bond strength is reproduced quantitatively for all examined Si–Si bonds. The Löwdin charges in Figure 11b also propose a strong similarity in the underlying bonding mechanisms in La/CeSi as well as the respective hydrides. It should be noted that the Löwdin charges are slightly larger in the lanthanum-containing structures for all respective elements. The respective *C*- and *P*-structures possess identical charges despite the small differences in covalency as determined by ICOBI.

**Figure 11:**
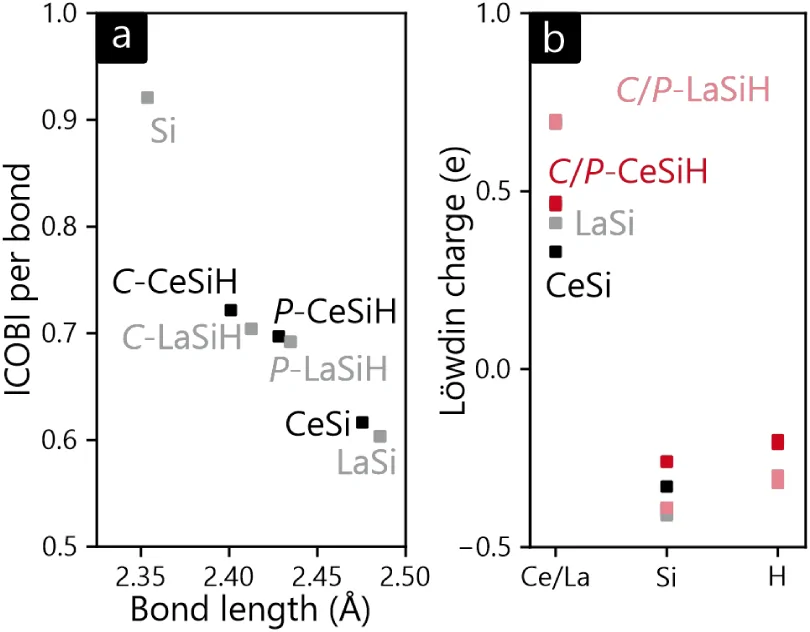
a) Comparison of ICOBI as a function of interatomic distance for CeSi, *C*/*P*-CeSiH, LaSi *C*/*P*-LaSiH, and elemental Si. The well-known correlation of shorter bonds/higher bond order is found throughout all compounds. b) Löwdin charges of all elements in CeSi, LaSi, and their hydrides. There is little difference between the isostructural compounds, while charges of lanthanum-containing phases are generally larger.

### 3.4 Magnetic Properties

As a basic characterization, the temperature dependence of the magnetic susceptibility data of CeSi was measured. The data of CeSi obtained by zero-field-cooling (ZFC) and zero-field-cooling/field-cooling (ZFC/FC) experiments measured at an external magnetic flux density of 10 kOe and 100 Oe is shown in Figure 12. Above 50 K, we observe classical Curie–Weiss paramagnetic behavior (2.43(1) μ_B_ Ce atom^–1^ and *Θ*_P_ = 0.87(2) K), in agreement with the earlier data reported by Shaheen.^16^ CeSi has a stable antiferromagnetic ground state and a comparatively low Néel temperature of *T*_N_ = 5.7(1) K. The magnetic structure of CeSi was first deemed to be incommensurate but later corrected to be commensurate.^14,15^

**Figure 12:**
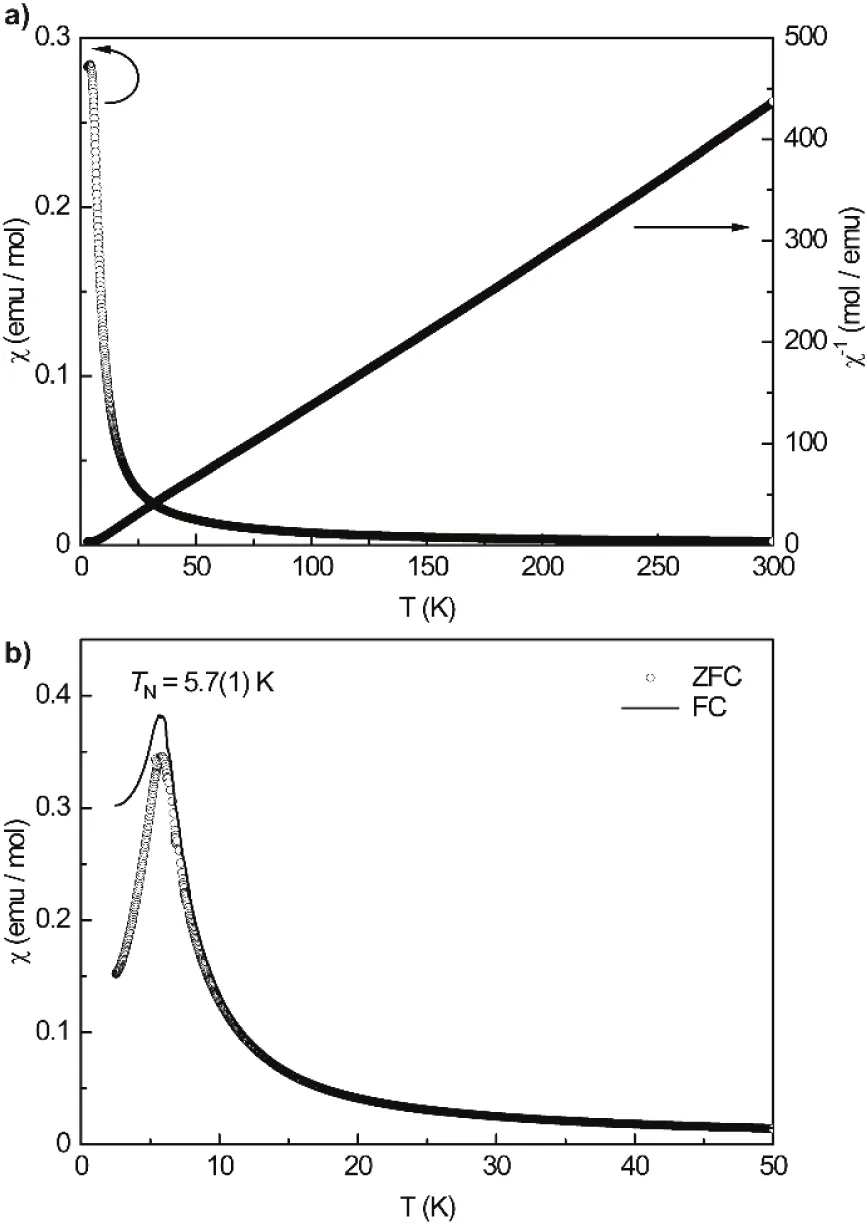
Temperature dependence of the magnetic susceptibility of CeSi measured at a) 10 kOe zero-field-cooling (ZFC) and b) at 100 Oe in zero-field-cooling/field-cooling (ZFC/FC) mode.

Hydrogenation and deuteration of the CeSi samples lead to a drastic change of the magnetic ground state and the ordering temperature. High- and low-field data of CeSiH_*x*_ collected in zero-field-cooling (ZFC) and zero-field-cooling/field-cooling (ZFC/FC) at 10 kOe and 100 Oe, respectively, clearly indicate ferromagnetic ordering (Figure 13). From the high-field data (Figure 13a), an effective magnetic moment of μ_eff_ = 2.53(1) μ_B_ Ce atom^–1^ and *Θ*_P_ = 25.3(1) K were deduced. The effective magnetic moment matches the literature value almost perfectly (μ_eff_(Ce^3+^) = 2.54(1) μ_B_ ^55^ indicating a stable trivalent ground state, while the Weiss constant indicates dominant ferromagnetic interactions in the paramagnetic temperature range. The sample shows small deviations from Curie–Weiss behavior in going to lower temperatures. This might be attributed to small amounts of impurity phases. It is well-known that trace amounts of iron in commercial cerium might affect the magnetic data.

**Figure 13:**
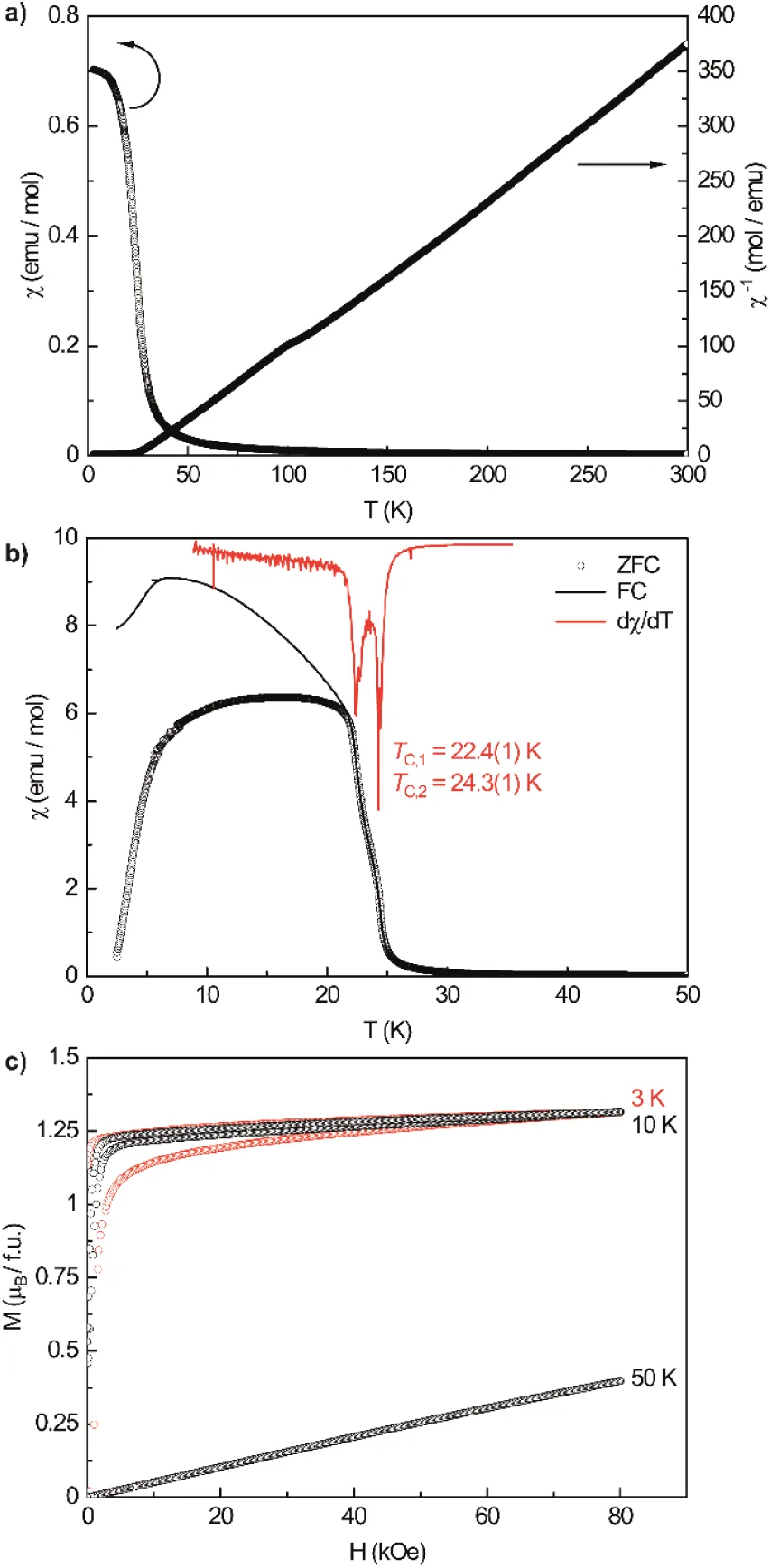
Temperature dependence of the magnetic susceptibility of CeSiH*_x_* measured at a) 10 kOe zero-field-cooling (ZFC) and b) 100 Oe in zero-field-cooling/field-cooling (ZFC/FC) mode. The derivative d*χ*/d*T* of the field-cooling curve is shown in red, emphasizing the two Curie temperatures. c) Magnetization isotherms recorded at 3 (red), 10, and 50 K (black).

The field-cooling curve does not show a monotonic increase (Figure 13b) but hints at two independent ordering phenomena. This becomes clear from the derivative d*χ*/d*T* which shows Curie temperatures of *T*_C,1_ = 22.4(1) and *T*_C,2_ = 24.3(1) K, respectively. The observation of two magnetic ordering phenomena is in line with the two observed phases in the diffraction experiments. However, an assignment of the magnetic transitions to the respective crystallographic phases is not possible based on the magnetization experiments (*vide infra*). The magnetization isotherms (Figure 13c) finally confirm the ferromagnetic ground state since the 3 and 10 K isotherms show a steep increase already at low fields. At 3 K and 80 kOe, the magnetization reaches 1.32(1) μ_B_, which is significantly below the theoretical saturation magnetization of 2.14 μ_B_ according to *g*_J_ × *J*. However, the saturation magnetization matches the expected value for a randomly oriented polycrystalline powder of 1.36 μ_B_ quite well, which is 64% of the calculated saturation magnetization.

As magnetism can be sensitive to even small structural changes, the magnetic behavior of CeSiD_*x*_ was also studied and can be found in the Supporting Information (Figure S16). The magnetic moments are again in agreement with Ce^3+^ ions, while the Curie temperatures are about 0.3 K higher. The difference seems reasonable, as the decreased thermal vibration of the deuterides lowers the lattice parameters and therefore reduces the Ce–Ce distances (*vide infra*).

The remarkable feature of the magnetic properties of CeSiH_*x*_/CeSiD_*x*_ is the high magnetic ordering temperature. Usually, such intermetallic cerium compounds exhibit fairly low transition temperatures. A good example is the enormously large family of equiatomic intermetallic cerium compounds Ce*TX* and Ce*XX*′ (*T* = transition metal; *X* = *p* element of the third, fourth, or fifth main group).^7–9,11^ The vast majority of these intermetallics show magnetic ordering (if present at all) below 10 K. Remarkable exceptions are the 17.5 K ferromagnet CePdSb^56^ and the 46 K antiferromagnet CeScGe.^57^ Already in 1970, Hill proposed a criterion (the so-called *Hill limit*)^58^ for discriminating between superconductivity and magnetic ordering in intermetallic cerium compounds. This criterion relies on the shortest Ce–Ce spacing in a given crystal structure. The Hill limit of ca. 3.4 Å then discriminates the superconducting phases below and the magnetically ordering ones above. This criterion has been resumed by Koelling^59^ and most recently in a broader context by Miyahara et al.^60^ They analyzed more than 700 intermetallic cerium compounds with respect to their Ce–Ce distances and magnetic ordering temperatures, nicely extending the original Hill plot. From their systematic study, it readily turned out that only a few of these phases exhibit high ordering temperatures, i.e., 14 compounds have ordering temperatures above 18 K. The three striking examples are CeRh_3_B_2_ (*T*_C_ = 115 K),^61^ Ce_5_NiPb_3_ (*T*_C_ = 48 K),^62^ and Ce_5_Pb_3_O (*T*_C_ = 46 K).^63^ The situation is most complex (and outstanding) for CeRh_3_B_2_, where cerium is in an intermediate-valent state and the high ferromagnetic ordering temperature is most likely mediated by the rhodium 4d electrons. Ce_5_NiPb_3_ exhibits the highest *T*_C_ when considering pure 4f magnetism. Miyahara et al. concluded that the prerequisite for a high *T*_C_ for an intermetallic cerium compound should be short Ce–Ce distances along with a suppression of a valence instability. Thus, CeSiH_*x*_ (CeSiD_*x*_) belongs to this remarkable class of cerium intermetallics as well.

For the pair CeSi (3.767(2) Å, Table 1) and CeSiH (3.787(3) Å, Table S1), there is only a very small difference, and the Ce–Ce distances alone might not be the intrinsic reason for the drastic change in the magnetic properties. The hydrogen incorporation changes the electronic structure (*vide supra*) and thus the RKKY interactions. For determining the exact crystal field parameters, inelastic neutron scattering experiments are required.

### 3.5 The Magnetic Structures of *C*-CeSiD*_x_* and *P*-CeSiD*_x_*

In order to assign the two hydride/deuteride phases to their respective Curie temperatures and to determine their magnetic structures, temperature-dependent NPD was performed between 10 and 50 K (Figure 14). During the measurements, both phases showed magnetic contributions below 24 and 22 K, respectively. A Rietveld refinement was performed at 30 K (Figure 15) as the base for the determination of the magnetic structure. Curiously, the refined deuterium content appeared smaller for the *C*- than for the *P*-phase, although previous refinements on the same sample (Figure 2) yielded the opposite. This might be a consequence of the temperature as described in Section 3.1. Another cause could have been the two-week break between measurements at D2b and D20; however, the kinetics of hydrogen release from *LnTt* hydrides have not been studied yet. At 10 K (Figure S18) all magnetic contributions were located on top of already existing reflections. The propagation vector of both phases was therefore determined to be *k* = 0. The refinement of these magnetic contributions is shown in Figure 16. The symmetry analysis was done using the BasIreps^64^ program within the FullProf suite^30^ with Ce atoms on the Wyckoff position 4*c* for both phases. For the *C*-phase, six irreducible representations (irreps), each having one basis vector (BV), were found, with three of those allowing a ferromagnetic coupling. The best refinement of the experimental reflections was achieved using a ferromagnetic structure with the magnetic moments in the *c* direction. The corresponding irrep has only one BV. By symmetry considerations, the Shubnikov group was derived to be *Cm'c'm* (Figure 17b). For the *P*-phase eight irreps having one or two BV’s were found. The best refinement was achieved with a ferromagnetic structure featuring magnetic moments in the *b* direction, giving better results than the two other irreps with ferromagnetic BV’s. This solution has again only one BV. The corresponding Shubnikov group was determined to be *Pn'ma'* (Figure 17a).

**Figure 14:**
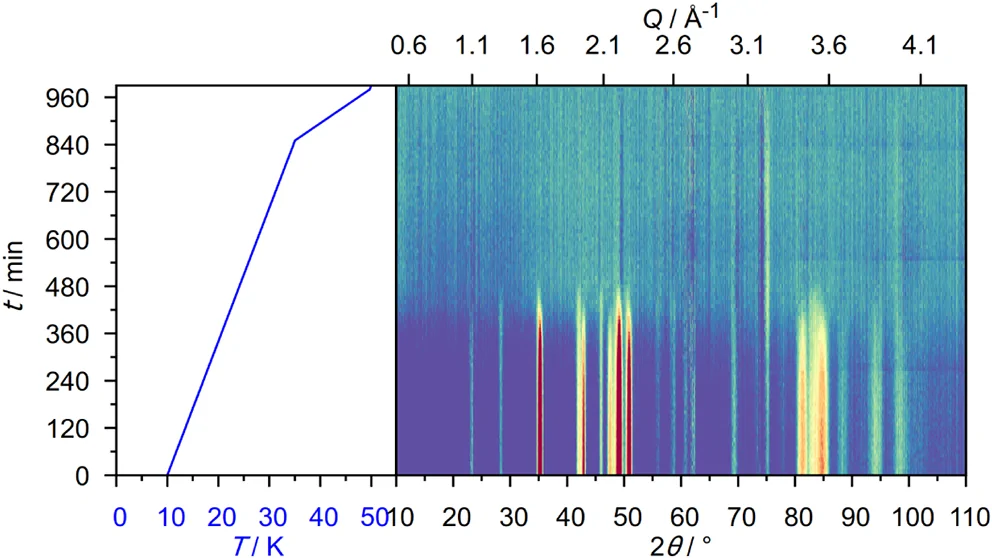
Pseudocolor image of the temperature-dependent magnetic contributions (intensity differences between current temperature and 50 K) in sample A (*C*- to *P*-phase ratio of roughly 2:1; D20, ILL Grenoble, France, *λ* = 2.4166(2) Å), NUMOR: 256666–256765.^27^

**Figure 15:**
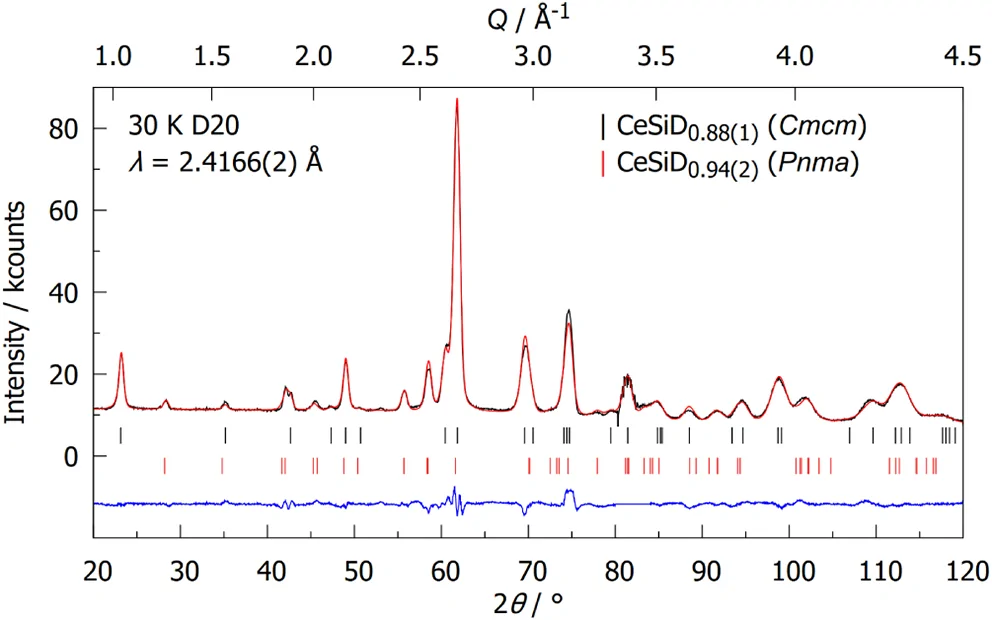
Rietveld refinement of the crystal structures of *C*-CeSiD_0.88(1)_ (71(2) w.-%) and *P*-CeSiD_0.94(2)_ (29(1) w.-%) on neutron powder diffraction pattern at 30 K, taken at D20 (Grenoble, France) at *λ* = 2.4166(2) Å, *R*_p_ = 2.48%, *R*_wp_ = 3.40%, *χ* ^2^ = 14.9, NUMOR: 256734.^27^

**Figure 16:**
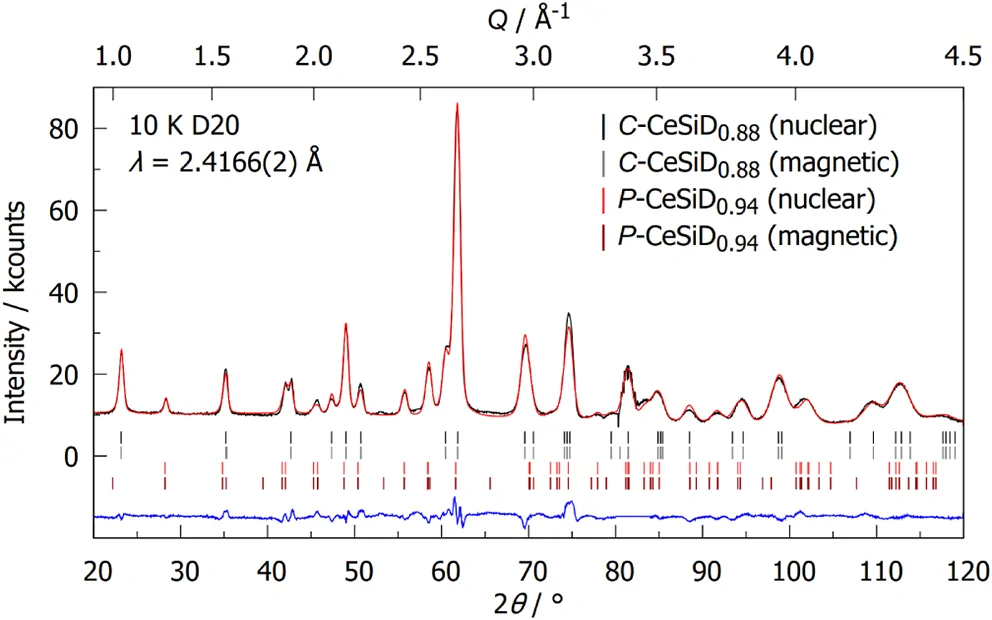
Rietveld refinement of the nuclear and magnetic structures of *C*-CeSiD_0.88_ (71 w.-%) and *P*-CeSiD_0.94_ (29 w.-%) on neutron powder diffraction pattern at 10 K, taken at D20 (Grenoble, France) at *λ* = 2.4166(2) Å, *R*_p_ = 3.28%, *R*_wp_ = 4.24%, *χ* ^2^ = 22.8, *m*_Ce_(*C*-phase) = 1.89(2) μ_B_, *R*_mag_(*C*-phase) = 7.46%, *m*_Ce_(*P*-phase) = 1.77(6) μ_B_, *R*_mag_(*P*-phase) = 9.22%, NUMOR: 256666.^27^

**Figure 17:**
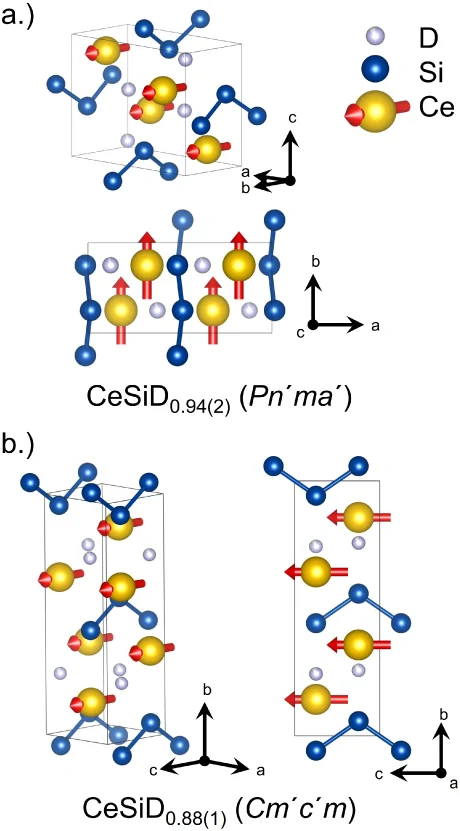
Crystal and magnetic structures of a) CeSiD_0.94(2)_ (*Pn′ma′*) and b) CeSiD_0.88(1)_ (*Cm′c′m*) at 10 K.

To assign the deuterides to their Curie temperatures, the magnetic intensities were obtained by subtracting the 50 K measurement from all patterns at lower temperatures (Figure 14). Using the lattice parameters and atom positions of the 30 K measurement (Figure 15), the two magnetic structures were refined on each pattern (Figures 18, 19). For *C*-CeSiD_0.88_, the last refinement was performed at 23.3 K, and for *P*-CeSiD_0.94_, at 24.1 K, as no more reflections were visible. The resulting magnetic moments *m* were then fitted according to the Landau theory as a second-order phase transition (eq 2). The fitted parameters (Table S8) alongside their plots (Figures S20, S21) can be found in the Supporting Information.

2 $$m=A\times {\left(\frac{\left(T_{c}-T\right)}{T_{c}}\right)}^{\beta }$$

**Figure 18:**
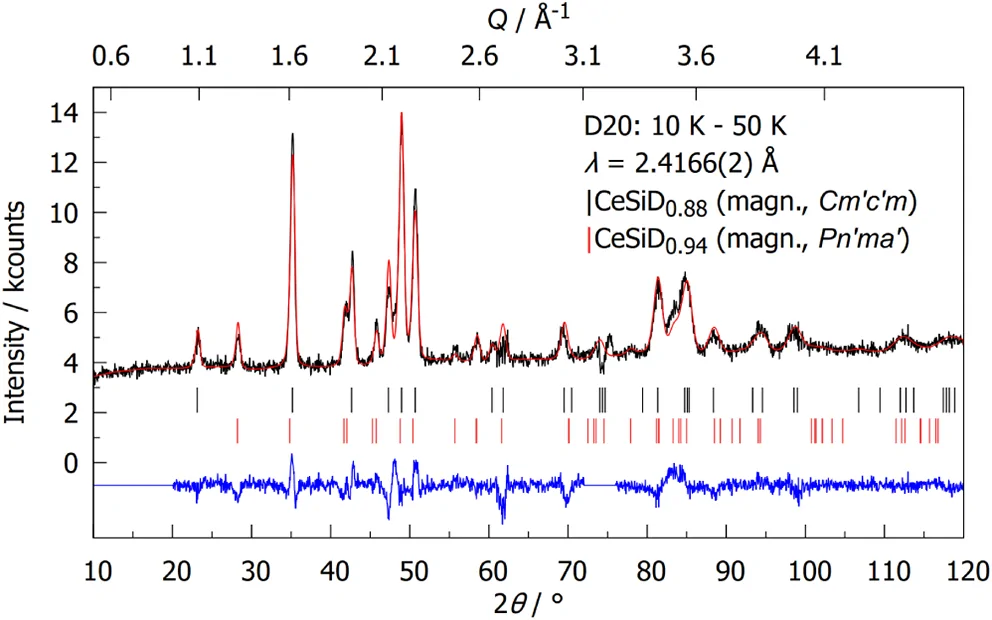
Rietveld refinement on the magnetic contribution (difference between 10 and 50 K measurement) of sample A consisting of *C*-CeSiD_0.88_ (71 w.-%) and *P*-CeSiD_0.94_ (29 w.-%) taken at D20 (Grenoble, France) at *λ* = 2.4166(2) Å, *R*_p_ = 3.93%, *R*_wp_ = 5.07%, *χ* ^2^ = 2.39, *m*_Ce_(*C*-phase) = 2.022(7) μ_B_, *R*_mag_(*C*-phase) = 7.76%, *m*_Ce_(*P*-phase) = 1.59(2) μ_B_, *R*_mag_(*P*-phase) = 12.5%, NUMOR: Difference of (256666–265765).^27^

**Figure 19:**
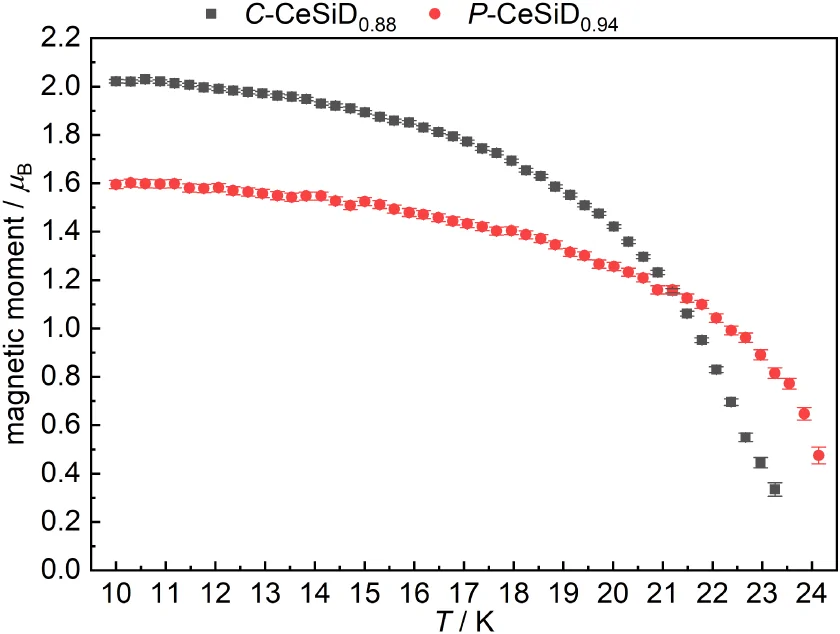
Refined magnetic moments of sample A from 10 to 24.1 K. The last three refinements were done with a magnetic moment of 0 for *C*-CeSiD_0.88_, NUMOR: 256666–256714.^27^

According to the phase-transition fits, the higher Curie temperature of *T*_C_ = 24.0(1) K was assigned to *P*-CeSiD_0.94_, while *C*-CeSiD_0.88_ exhibits a slightly lower *T*_C_ of 22.43(8) K. For the *P*-phase, these values match reasonably well with *T*_C_ = 24.6(1) K (*P*-CeSiH_*x*_) and *T*_C_ = 24.3(1) K (*P*-CeSiD_*x*_), determined by ZFC-FC measurements (Figures 13 and S16b). For the *C*-phase, due to the wiggly trace in the deuteride ZFC-FC measurement (Figure S16b), only the *C*-hydride with *T*_C_ = 22.4(1) K (*C*-CeSiH_*x*_) can be compared, which also matches well.

At 10 K, the ordered magnetic moments are *m*_Ce_ = 2.022(7) μ_B_ for the *C*-phase and *m*_Ce_ = 1.59(2) μ_B_ for the *P*-phase (Figure 18). The *C*-phase therefore compares well to the theoretical value of 2.14 μ_B_ according to *g*_J_ × *J*,^55^ while the *P*-phase shows a significantly lower value. A lower value for the *P*-deuteride was also observed for another sample of CeSiD_*x*_ (*vide infra*). Values below 2.14 μ_B_ are commonly observed for Ce-containing intermetallics. They may be caused by crystal electric fields as well as Kondo interactions. Given the generally lower hydride/deuterium contents in the *P*-phase (with the exception of this specific measurement), it might be plausible that the Ce^3+^ valence is less stabilized than in the *C*-phase, leading to a weaker magnetic moment. It is well-known that hydrogen uptake in intermetallic phases can drastically change the magnetic properties.^65,66^

Notably, the fits of eq 2 to the magnetic moments (Figure 19) show considerable discrepancies around *T*_C_ (Figures S20, S21) as well as toward 10 K. Furthermore, the values of *β* = 0.233(12) and 0.216(10) in Table S8 do not match the expected value for a magnetic phase transition of 0.33. This might hint at deviations from a second-order phase transition behavior. Parameter *A*, which corresponds to the ordered magnetic moment, also seems overestimated, as the *C*-phase’s 2.41(3) μ_B_ is well above the expected 2.14 μ_B_. Due to the lower time resolution and the inherent issue of determining when exactly a low-intensity reflection completely vanishes, the *T*_C_ obtained from ZFC-FC measurements should be considered to be more reliable.

Additionally, another sample of CeSiD_*x*_ was measured via NPD at 50 K (Figure S22, Tables S9, S10) and 1.6 K (Figure S23, Tables S11, S12). The refined values of μ­(Ce^3+^, *C*-phase) = 1.88(6) μ_B_ and μ­(Ce^3+^, *P*-phase) = 1.63(5) μ_B_ are not significantly different from those derived from the measurement at 10 K (Figure 17) within ±2σ. Between 50 and 1.6 K, CeSiD_*x*_ showed no signs of a phase transition or a change in magnetic behavior.

Given the relatively similar Ce–Ce distances in comparison to CeSi (Tables 1–3), the remarkably high *T*_*C*_ of the hydrides (deuterides) seems to stem from the changes in the electronic rather than the geometric structure. Between the *P*- and the *C*-phase, however, the picture is more complex. On one hand, the *C*-phase is the thermodynamically favored product and generally reaches higher hydrogen contents, so one would expect a stronger suppression of valence instability than in the *P*-phase. On the other hand, the shortest Ce–Ce distance was recorded for the *P*-phase (Table 2). In the direct comparison, the shorter Ce–Ce distances in the *P*-phase seem to overshadow a possible difference in Ce valence, leading to the slightly higher Curie temperature.

## 4 Conclusion

Hydrogenation (deuteration) of CeSi results in two hydrides. The *P*-phase retains the overall FeB-type structure in space group *Pnma*: CeSiD_0.82(2)_ (LaGeH type), *a* = 8.0859(5) Å, *b* = 4.0067(3) Å, *c* = 6.2801(4) Å. The *C*-phase is a CrB-type-based structure in space group *Cmcm*: CeSiD_0.96(1)_ (ZrNiH type), *a* = 4.2414(2) Å, *b* = 12.0765(4) Å, *c* = 3.9927(2) Å. Both structures feature hydrogen-filled Ce_4_ quasi-tetrahedral sites and can reach hydrogen *SOF*s close to or equal to 1, resulting in the composition CeSiH. Compared to pristine CeSi, the *P*-phase shows a volumetric increase of 3.6% and the *C*-phase a more pronounced increase of 4.1%. Higher temperatures favor the *C*-phase; a hydrogen pressure dependency was, however, not observed. The theoretical examination of both CeSiH phases revealed slight but significant differences that cause a thermodynamic preference for *C*-CeSiH and its preferred formation in the hydrogenation reaction of CeSi, while *P*-CeSiH is formed first as the kinetic product. Likewise, the Si–Si bond order increases along CeSi < *P*-CeSiH < *C*-CeSiH, leading to a gradual decrease in Si–Si distance from CeSi to *P*- and *C*-CeSiD_*x*_. Experimentally, however, the *P*-phase was observed to retain the Si–Si intrachain distances and bond angles of CeSi, whereas the *C*-phase showed shorter bonds and wider angles, at least at room temperature and above.

Both phases order ferromagnetically with an effective magnetic moment of μ­(Ce^3+^) = 2.53 μ_B_ which is very close to the free ion value of 2.54 μ_B_. Remarkable for both phases are their Curie temperatures of 22.4(1) K for *C*-CeSiH_*x*_ and 24.3(1) K for *P*-CeSiH_*x*_, which are exceptionally high for cerium intermetallics. The respective deuterides exhibit about 0.3 K higher Curie temperatures. In both cells, the magnetic moments are oriented alongside the polyanionic Si–Si chains with a propagation vector of *k* = 0. This behavior is in stark contrast to CeSi, which is an antiferromagnet with a Néel temperature of *T*_N_ = 5.7(1) K.

For the differences in magnetic behavior, both geometric and electronic effects have to be considered. As the step from CeSi to the two hydrides is only accompanied by small changes in Ce–Ce distances, the leap in *T*_C_ should predominantly stem from the changed electronic structure. Hydrogen localizes Ce-d electrons, which suppresses the Kondo screening of 4f electrons and enables the strong exchange interaction leading to high Curie temperatures.

Between the two hydrides, however, the amount of hydrogen incorporated is often comparable, leading to similar electronic states. Here the small difference in *T*_C_ seems to stem from the Ce–Ce distance, as it was consistently shorter in the *P*-hydride, leading to a higher *T*_C_. Between the hydrides and the deuterides, the small increase in *T*_C_ of around 0.3 K for the deuterides is likely caused by the reduced thermal displacement and, therefore, even smaller Ce–Ce distances.

## Supplementary Material



## Notes

Rietveld refinements of the crystal structures of CeSi, and CeSiH_*x*_ based on on XRPD; structural parameters of CeSiH_*x*_; SEM images of CeSiD_*x*_; the full *in situ* deuteration experiment on CeSi; thermal analysis (DSC) of CeSi under H_2_; parameters of the Birch–Murnaghan fit of CeSiH; magnetic properties determination on CeSiD_*x*_; the 2nd order phase transition fits on CeSiD_*x*_ and refinements on the NPDs of CeSiD_*x*_ (at 50 and 1.6 K); table of refined structural parameters at different temperatures and devices (PDF)

## References

[1] Ott H. R. (1987). Heavy-Electron
Metals. Annu. Rev. Mater. Sci..

[2] O̅nuki Y., Settai R., Sugiyama K., Takeuchi T., Kobayashi T. C., Haga Y., Yamamoto E. (2004). Recent Advances
in the Magnetism
and Superconductivity of Heavy Fermion Systems. J. Phys. Soc. Jpn..

[3] Kumar R. S., Kohlmann H., Light B. E., Cornelius A. L., Raghavan V., Darling T. W., Sarrao J. L. (2004). Anisotropic
Elastic
Properties of CeRhIn_5_. Phys. Rev.
B: Condens. Matter Mater. Phys..

[4] Steglich F. (2005). Superconductivity
and Magnetism in Heavy-Fermion Compounds. J.
Phys. Soc. Jpn.

[5] Kitaoka Y., Kawasaki S., Mito T., Kawasaki Y. (2005). Unconventional Superconductivity
in Heavy-Fermion Systems. J. Phys. Soc. Jpn.

[6] Matar S. F. (2013). Review
on Cerium Intermetallic Compounds: A Bird’s Eye Outlook through
DFT. Prog. Solid State Chem.

[7] Pöttgen R., Janka O., Chevalier B. (2016). Cerium Intermetallics
Ce*TX* – Review III. Z.
Naturforsch. B: J.
Chem. Sci..

[8] Pöttgen R., Chevalier B. (2015). Cerium Intermetallics with ZrNiAl-Type Structure –
a Review. Z. Naturforsch. B: J. Chem. Sci..

[9] Janka O., Niehaus O., Pöttgen R., Chevalier B. (2016). Cerium Intermetallics
with TiNiSi-Type Structure. Z. Naturforsch.
B: J. Chem. Sci..

[10] Wiesinger, G. ; Hilscher, G. Chapter Five Magnetism of Hydrides. In Handbook of magnetic materials, Buschow, K. H. J. , Eds.;Elsevier: Amsterdam, 2007; Vol. 17, pp. 293–456.

[11] Pöttgen R., Chevalier B. (2015). Equiatomic Cerium Intermetallics Ce*XX* ′ with Two *p* Elements. Z. Naturforsch. B: J. Chem. Sci..

[12] Hohnke D., Parthé E. (1966). *AB* Compounds with ScY and Rare Earth
Metals. II. FeB and CrB Structures of Monosilicides and Germanides. Acta Crystallogr..

[13] Gladyshevskii E. I., Kripyakevich P. I. (1964). Monosilicides of Rare Earth Metals
and Their Crystal
Structures. Zh. Strukt. Khim..

[14] Schobinger-Papamantellos P., Buschow K. H. (1994). Incommensurate Magnetic Structure of CeSi as Observed
by Neutron Diffraction. J. Magn. Magn. Mater..

[15] Schobinger-Papamantellos P., Kenzelmann M., Schenck A., Gygax F. N., Buschow K. H. J., Ritter C. (2004). Magnetic Properties
of the CeGe1–*x*Si*
_x_
* Series Studied by Powder Neutron
Diffraction and *μ*SR-Measurements. Phys. B.

[16] Shaheen S. A. (1988). CeSi: A
Non-Kondo Trivalent Ce Compound. J. Appl. Phys..

[17] Werwein A., Auer H., Kuske L., Kohlmann H. (2018). From Metallic *LnTt* (*Ln* = La*Tt* = Si,
Ge, Sn) to Electron-precise Zintl Phase Hydrides *LnTt*H. Z. Anorg. Allg. Chem..

[18] Parthé E. (1976). The CrB and
Related Structure Types Interpreted by Periodic Unit-Cell Twinning
of Close-Packed Structures. Acta Crystallogr.,
Sect. B.

[19] Hashimoto K., Kurosaki K., Imamura Y., Muta H., Yamanaka S. (2007). Thermoelectric
Properties of BaSi_2_, SrSi_2_, and LaSi. J. Appl. Phys..

[20] Bohmhammel K., Henneberg E. (2001). Hydriding
And Dehydriding Behavior Of Lanthanum Silicides. Solid State Ionics.

[21] Werwein A., Hansen T. C., Kohlmann H. (2019). Size Matters: New Zintl
Phase Hydrides
of *RE*Ga (*RE* = Y, La, Tm) and *RE*Si (*RE* = Y, Er, Tm) with Large and Small
Cations. Crystals.

[22] Häussermann U., Kranak V. F., Puhakainen K. (2010). Hydrogenous Zintl Phases: Interstitial
Versus Polyanionic Hydrides. Struct. Bonding.

[23] Pöttgen R., Gulden T., Simon A. (1999). Miniaturisierte
Lichtbogenapparatur
für den Laborbedarf. GIT Labor-Fachz..

[24] Hewat A. W. (1986). D2B, A
New High Resolution Neutron Powder Diffractormeter at Ill Grenboble. Mater. Sci. Forum.

[25] Kohlmann, H. ; Dorsch, L. Y. ; Pöttgen, R. ; Ritter, C. Crystal structure and magnetism of CeSiD. Institut Laue-Langevin (ILL). 2024, 10.5291/ILL-DATA.5-24-753.

[26] Hansen T. C., Henry P. F., Fischer H. E., Torregrossa J., Convert P. (2008). The D20 Instrument at the ILL: A Versatile High-Intensity
Two-Axis Neutron Diffractometer. Meas. Sci.
Technol..

[27] Ritter, C. Follow up of D2b experiment 5–24–753. Institut Laue-Langevin (ILL). 2024, 10.5291/ILL-DATA.EASY-1381.

[28] Finger R., Kurtzemann N., Hansen T. C., Kohlmann H. (2021). Design and use of a
sapphire single-crystal gas-pressure cell for *in situ* neutron powder diffraction. J. Appl. Crystallogr..

[29] Sturza, M. I. ; Dorsch, L. Y. ; Goetze, A. ; Hansen, T. C. ; Kohlmann, H. ; Michak, M. ; Sikolenko, V. In situ investigations of the hydrogenation of Ni_3_In; Institut Laue-Langevin (ILL), 2023, 10.5291/ILL-DATA.5-24-720.

[30] Rodríguez-Carvajal J. (1993). Recent Advances
in Magnetic Structure Determination by Neutron Powder Diffraction. Phys. B (Amsterdam, Neth.).

[31] Williams, T. ; Kelley, C. Gnuplot Version 6.0 (Patchlevel 1); Gnuplot, 2024.

[32] OriginPro 2019 (Version 9.6.0.172); OriginLab Corporation: Northampton, Massachusetts, USA, 2019.

[33] Momma K., Izumi F. (2011). VESTA 3 for Three-Dimensional Visualization of Crystal, Volumetric
and Morphology Data. J. Appl. Crystallogr..

[34] OriginPro 2024 (version 10.1.0.170); OriginLab Corporation: Northampton, Massachusetts, USA, 2024.

[35] CorelDRAW Graphics Suite 2017 (version 19.0.0.328); Ottawa: Corel Corporation, 2017.

[36] Kresse G., Hafner J. (1993). Ab Initio Molecular
Dynamics for Liquid Metals. Phys. Rev. B: Condens.
Matter Mater. Phys..

[37] Kresse G., Furthmüller J. (1996). Efficiency of Ab-Initio Total Energy Calculations for
Metals and Semiconductors Using a Plane-Wave Basis Set. Comput. Mater. Sci..

[38] Kresse G., Furthmüller J. (1996). Efficient Iterative Schemes for Ab
Initio Total-Energy
Calculations Using a Plane-Wave Basis Set. Phys.
Rev. B: Condens. Matter Mater. Phys..

[39] Kresse G., Joubert D. (1999). From Ultrasoft Pseudopotentials
to the Projector Augmented-Wave
Method. Phys. Rev. B: Condens. Matter Mater.
Phys..

[40] Blöchl P. E. (1994). Projector
Augmented-Wave Method. Phys. Rev. B: Condens.
Matter Mater. Phys..

[41] Csonka G. I., Perdew J. P., Ruzsinszky A., Philipsen P. H. T., Lebègue S., Paier J., Vydrov O. A., Ángyán J. G. (2009). Assessing the Performance of Recent
Density Functionals for Bulk Solids. Phys. Rev.
B.

[42] Grimme S., Ehrlich S., Goerigk L. (2011). Effect of
the Damping Function in
Dispersion Corrected Density Functional Theory. J. Comput. Chem..

[43] Monkhorst H. J., Pack J. D. (1976). Special Points for Brillouin-Zone Integrations. Phys. Rev. B.

[44] Blöchl P. E., Jepsen O., Andersen O. K. (1994). Improved Tetrahedron
Method for Brillouin-Zone
Integrations. Phys. Rev. B: Condens. Matter
Mater. Phys.

[45] Nelson R., Ertural C., George J., Deringer V. L., Hautier G., Dronskowski R. (2020). LOBSTER: Local Orbital Projections, Atomic Charges,
and Chemical-Bonding Analysis from Projector-Augmented-Wave-Based
Density-Functional Theory. J. Comput. Chem..

[46] Ertural C., Steinberg S., Dronskowski R. (2019). Development of a Robust Tool to Extract
Mulliken and Löwdin Charges from Plane Waves and Its Application
to Solid-State Materials. RSC Adv..

[47] Müller P. C., Ertural C., Hempelmann J., Dronskowski R. (2021). Crystal Orbital
Bond Index: Covalent Bond Orders in Solids. J. Phys. Chem. C.

[48] Müller P. C., Schmit N., Sann L., Steinberg S., Dronskowski R. (2024). Fragment Orbitals Extracted from First-Principles Plane-Wave
Calculations. Inorg. Chem..

[49] Eck, B. wxDragon 2.2.3; RWTH Aachen University: Aachen, Germany, 2020.

[50] Auer H., Yang F., Playford H. Y., Hansen T. C., Franz A., Kohlmann H. (2019). Covalent Si–H
Bonds in the Zintl Phase Hydride
CaSiH_1+*x*
_ (*x* ≤
1/3). Inorganics.

[51] Armbruster M., Wörle M., Krumeich F., Nesper R. (2009). Structure
and Properties
of Hydrogenated Ca, Sr, Ba, and Eu Silicides. Z. Anorg. Allg. Chem..

[52] Wiberg K. B. (1968). Application
of the Pople-Santry-Segal CNDO Method to the Cyclopropylcarbinyl and
Cyclobu-Tyl Cation and to Bicyclobutane. Tetrahedron.

[53] Mayer I. C. (1983). Bond Order
and Valence in the Ab Initio SCF Theory. Chem.
Phys. Lett..

[54] Dronskowski R., E P. E. (1993). Crystal orbital Hamilton populations (COHP): energy-resolved visualization
of chemical bonding in solids based on density-functional calculations. J. Phys. Chem..

[55] Lueken, H. ; Magnetochemie, B. G. Teubner: Stuttgart, Leipzig, 1999.

[56] Katoh K., Takabatake T., Ochiai A., Uesawa A., Suzuki T. (1997). Quasi Two-Dimensional
Conductivity in CePtSb and CePdSb. Phys. B.

[57] Ritter C., Provino A., Manfrinetti P., Pathak A. K. (2017). Tetragonal to triclinic
structural transition in the prototypical CeScSi induced by a two-step
magnetic ordering: a temperature-dependent neutron diffraction study
of CeScSi, CeScGe and LaScSie. J. Phys.: Condens.
Matter.

[58] Hill, H. H. The early actinides: The periodic system’s f electron transition metal series. In Plutonium 1970 and other Actinides; Miner, W. ed., The Metallurgical Society of the AIME: New York; 1970, pp. 2–19.

[59] Koelling D. D. (1985). Cerium
Compounds in the Fashion of the Light Actinides. Phys. B+C (Amsterdam).

[60] Miyahara J., Shirakawa N., Setoguchi Y., Tsubota M., Kuroiwa K., Kitagawa J. (2018). Hill Plot Focusing
on Ce Compounds with High Magnetic
Ordering Temperatures and Consequent Study of Ce_2_AuP_3_. J. Supercond. Novel Magn..

[61] Givord F., Boucherle J.-X., Galéra R.-M., Fillion G., Lejay P. (2007). Ferromagnetism
and Crystal Electric Field in the Cerium Compound CeRh_3_B_2_. J. Phys.: Condens. Matter.

[62] Goruganti V., Rathnayaka K. D. D., Ross J. H. (2009). R = CeNd Magnetic Phase Transitions
in R_5_NiPb_3_and Gd). J.
Appl. Phys..

[63] Macaluso R. T., Moreno N. O., Fisk Z., Thompson J. D., Chan J. Y. (2004). Structure
and Magnetism of Ce_5_Pb_3_O. Chem. Mater..

[64] Rodríguez-Carvajal, J. B. BasIReps, A Program For Calculating Irreducible Representation Of Little Groups And Basis Functions Of Polar And Axial Vector Properties. Vers.: July-2010 Grenoble, France, 2010.

[65] Buschow, K. H. J. Hydrogen Absorption in Intermetallic Compounds. In Handbook on the Physics and Chemistry of Rare Earths, Gschneidner, K. A. ; Eyring, L. , Eds.; Elsevier Science, 1984, Vol. 6, 1.

[66] Buschow K.
H. J. (1980). Magnetic
properties of CeCo_3_, Ce_2_Co_7_ and CeNi_3_ and their ternary hydrides. J. Less-Common
Met..

