# Supplementary material for: Trapping the Excess Electron of FeB-Type CeSi by Hydrogenation — Formation of Ferromagnetic Hydrides CeSiH x (T C ≈ 24 K)
Source: Inorg Chem. 2026 Aug 4;65(32):18572–84. doi: 10.1021/acs.inorgchem.6c01660 (PMC13488391; doi:10.1021/acs.inorgchem.6c01660)
Supplement: Supplementary file 1 [file ic6c01660_si_001.pdf]

Supporting Information

Trapping the excess electron of FeB-type CeSi by hydrogenation – formation of ferromagnetic hydrides $CeSiH_x$ ($T_c \approx 24$ K)

Leonhard Y. Dorsch,[a] *Peter C. Müller,*[b] *Sarah Michels,*[b] *Clemens Ritter,*[c] *Anton Werwein,*[a] *Oliver Janka,*[d] *Jasper A. Baldauf,*[e] *Rainer Pöttgen,*[e] *Richard Dronskowski,*[b] *Holger Kohlmann**[a]

*Corresponding Author

[a] Leipzig University, Faculty of Chemistry, Institute of Inorganic Chemistry and Crystallography, Johannisallee 29, 04103 Leipzig, Germany. E-mail: holger.kohlmann@uni-leipzig.de

[b] Institute of Inorganic Chemistry, RWTH Aachen University, D-52056 Aachen, Germany.

[c] Institut Laue Langevin, 71 Avenue des Martyrs, BP 156, F-38042 Grenoble Cedex 9, France

[d] Inorganic Solid State Chemistry, Saarland University, Campus C4.1, 66123 Saarbrücken, Germany.

[e] Institut für Anorganische und Analytische Chemie, Universität Münster, Corrensstrasse 30, 48149 Münster, Germany.

Table of content:

SI 1. Rietveld refinements of CeSi, $CeSiH_x$ and structural parameters of $CeSiH_x$……………S3-S5

SI 2. SEM images of $CeSiD_x$……………………………………………………………………..S6

SI 3. Additional plots, refinements and tables from the *in situ* neutron deuteration of CeSi...S7-S13

SI 4. Thermal analysis of the hydrogenation of CeSi …………………………………… S14-S15

SI 5. Parameters of the Birch–Murnaghan fit of CeSiH…………………………………………S16

SI 6. Magnetic properties of $CeSiD_x$………………………………………………………S17-S18

SI 7. Magnetic reflections of $CeSiD_x$ and second order phase transition fits………………S19-S23

SI 8. Magnetic refinement of $CeSiD_x$ at 1.6 K……………………………………………...S24-S28

SI 9. Table of refined structural Parameters at different Temperatures and Devices..........…S29-S30

All references in the supporting information are identical to those found in the main text.

SI 1. Rietveld refinements of CeSi, $CeSiH_x$ and structural parameters of $CeSiH_x$

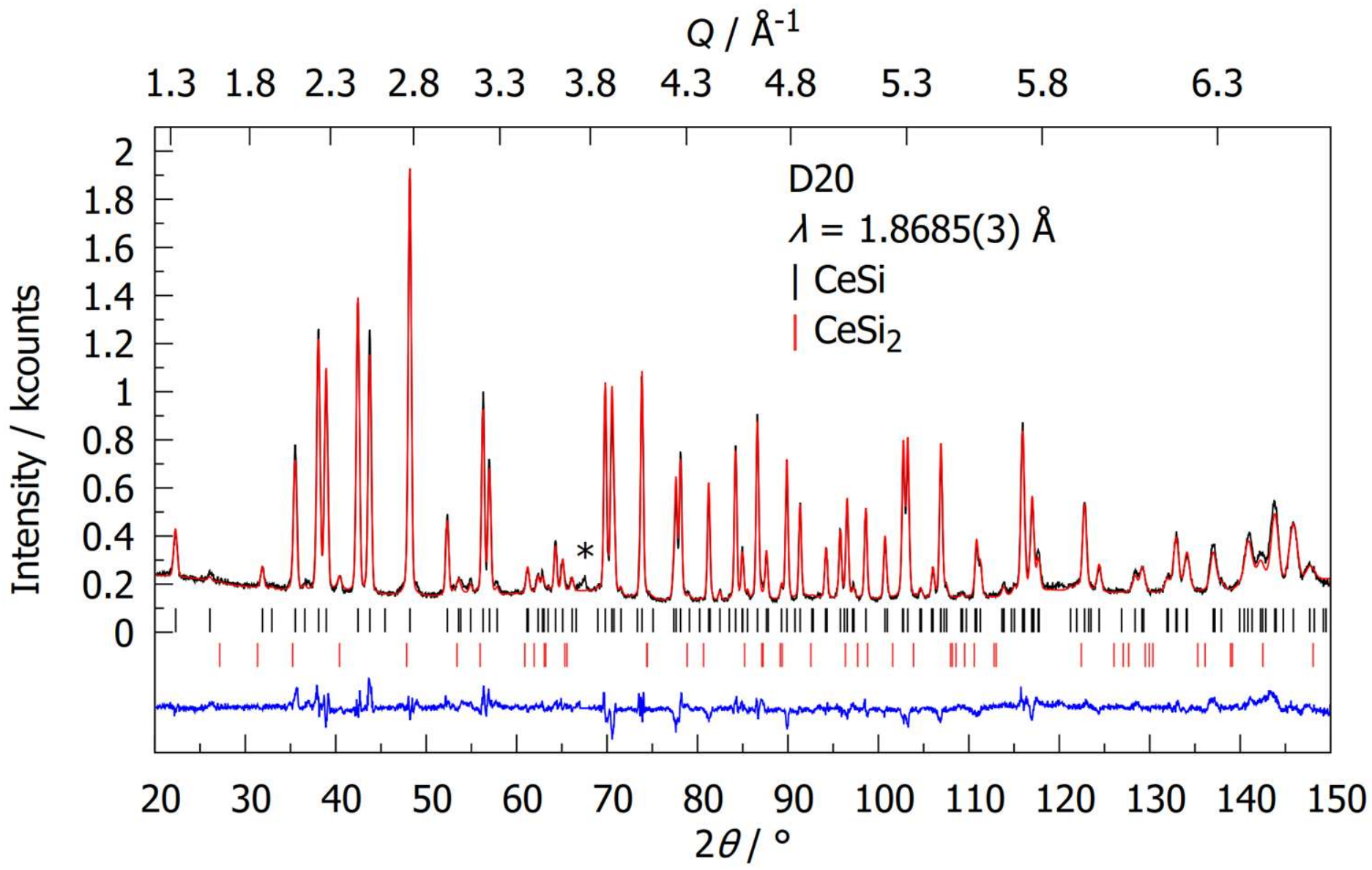

Figure S1: Rietveld refinement on the neutron powder diffraction pattern of CeSi in a sapphire single crystal cell taken before the *in situ* hydrogenation at D20 (ILL), corresponding to the structural data in Table 1, NUMOR: 244361.[29] * marks a parasitic reflection from the sapphire cell. CeSi: 97.2(9) w.-%, $R_{Bragg(CeSi)}$ = 8.07 %, $CeSi_2$: 2.8(2) w.-%, $R_{Bragg(CeSi2)}$ = 18.2 %, R_p = 5.07 %, R_{wp} = 6.28 %, χ^2 = 3.64.

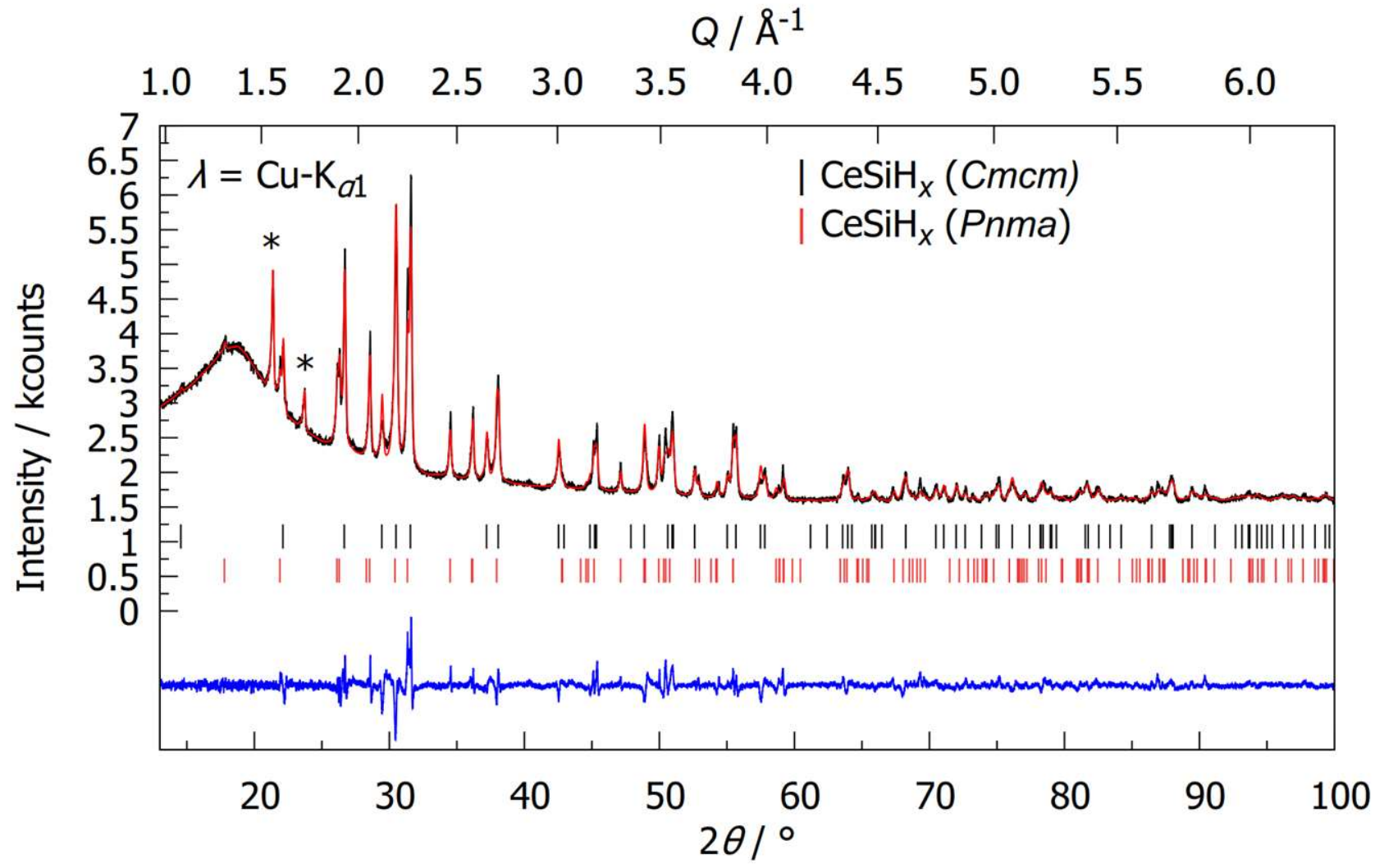

Figure S2: Rietveld refinement on the X-ray powder diffraction pattern of hydrogenated CeSi (6Mpa H_2 at 775 K for 48 h), Huber G670, *C*-$CeSiH_x$: 58.9(6) w.-%, $R_{Bragg(C\text{-}CeSiHx)}$ = 7.45 %, *P*-$CeSiH_x$: 41.1(5) w.-%, $R_{Bragg(P\text{-}CeSiHx)}$ = 7.82 %, R_p = 1.84 %, R_{wp} = 2.86 %, χ^2 = 1.71, * marks reflections caused by Apiezon grease that were treated as part of the background.

Table S1: Crystal structure parameters of *P*-$CeSiH_x$ and selected interatomic distances determined by X-ray powder diffraction at room temperature, values for hydrogen were taken from the deuteride *P*-$CeSiD_{0.82}$.

$CeSiH_{0.82}$ (*Pnma*)
a = 8.0821(4) Å, *b* = 4.0049(2) Å, *c* = 6.2837(3) Å, *V* = 203.39(2) Å^3

	Wyckoff site	*x*	*y*	*z*	B_{iso} / Å^2	*sof*
Ce	4*c*	0.1740(3)	¼	0.1238(3)	0.29(5)	1
Si	4*c*	0.035(1)	¼	0.587(2)	1.3(1)	1
H	4*c*	0.379	¼	0.422	1.61	0.82
Ce – Ce	3.787(3) Å / 3.923(2) Å / 4.0049(2) Å / 4.341(3) Å					
Si – Si	2.348(8) Å					

Table S2: Crystal structure parameters of *C*-$CeSiH_x$ and selected interatomic distances determined by X-ray powder diffraction at room temperature, values for hydrogen were taken from the deuteride *C*-$CeSiD_{0.96}$.

$CeSiH_{0.96}$ (*Cmcm*)
a = 4.2396(2) Å, b = 12.0918(5) Å, c = 3.9903(2) Å, V = 204.56(1) Å^3

	Wyckoff site	*x*	*y*	*z*	B_{iso} / Å^2	*sof*
Ce	4*c*	0	0.3548(1)	¼	B_{iso} (Ce, *P*-$CeSiH_{0.82}$)	1
Si	4*c*	0	0.0551(5)	¼	B_{iso} (Si, *P*-$CeSiH_{0.82}$)	1
H	4*c*	0	0.7607	¼	1.61	0.956
Ce – Ce						3.860(1) Å / 3.9903(2) Å / 4.039(2) Å / 4.2396(2) Å
Si – Si						2.399(5) Å

SI 2. SEM images of $CeSiD_x$

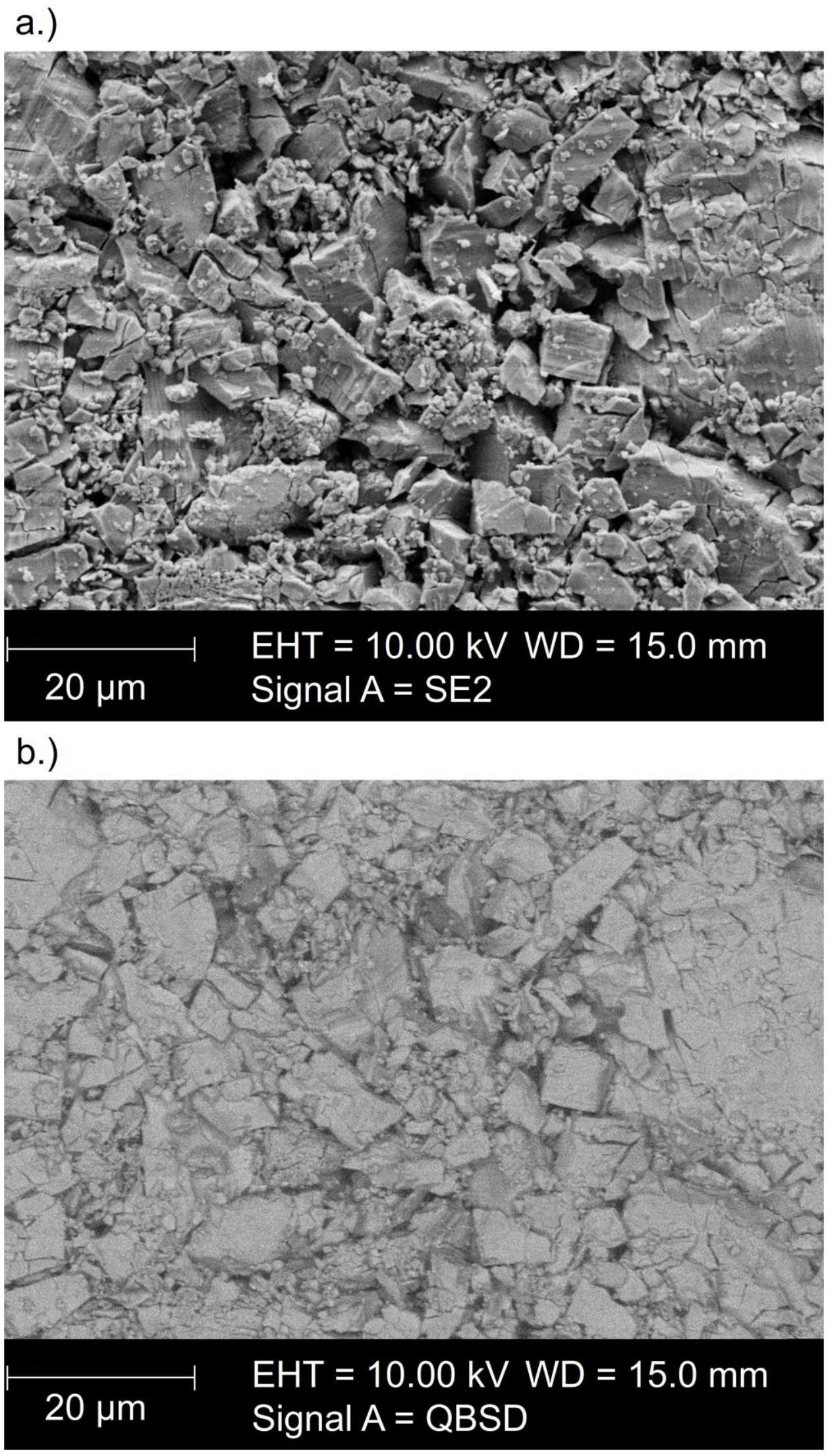

Figure S3: Scanning electron microscope images of a pressed pellet of Sample A ($CeSiD_x$) at a scale of 20 µm, a) secondary electron detector, b) back scatter electron detector.

SI 3. Additional plots from the *in situ* neutron deuteration of CeSi

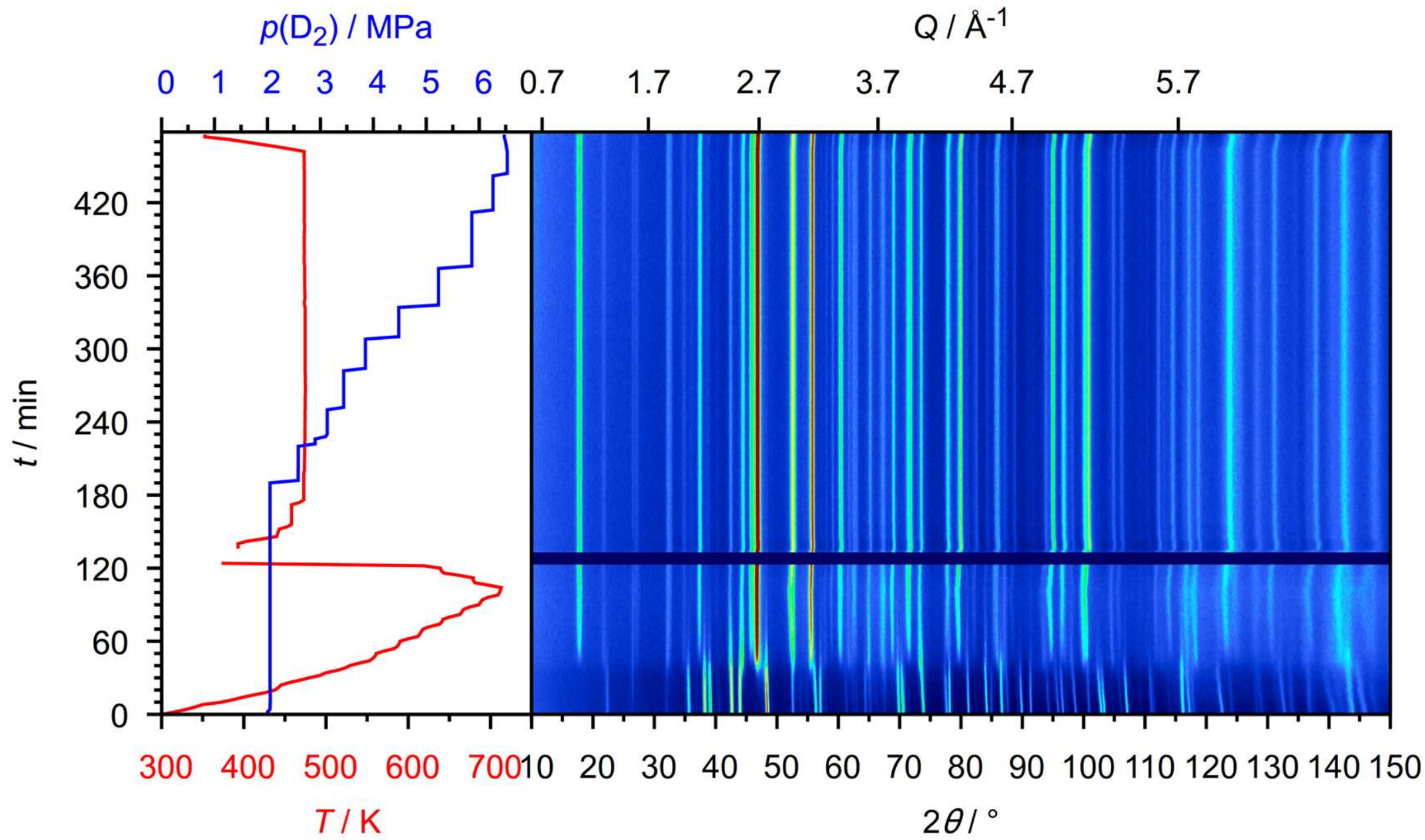

Figure S4: *In situ* neutron powder diffraction experiment on the deuteration of CeSi, the gap at the 120-minute mark signals a break due to necessary maintenance of the laser heater, coincidentally separating the temperature dependent from the pressure dependent part; D20 (ILL, λ = 186.85(3) pm, NUMOR: 244362 – 244595.[29]

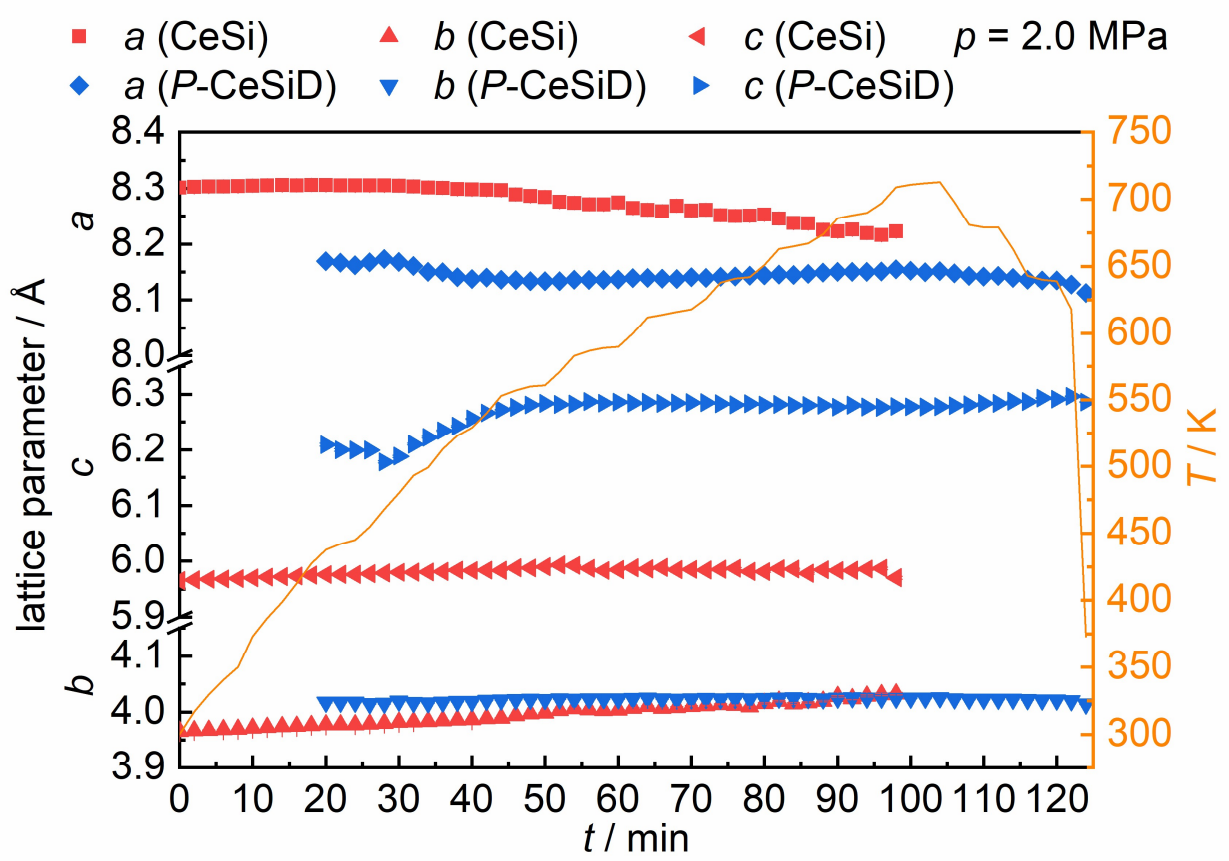

Figure S5: Comparison between the lattice parameters of CeSi and *P*-CeSiD during the temperature dependent part of the *in situ* deuteration of CeSi; errors are given as $\pm\sigma$ or smaller than the symbols.

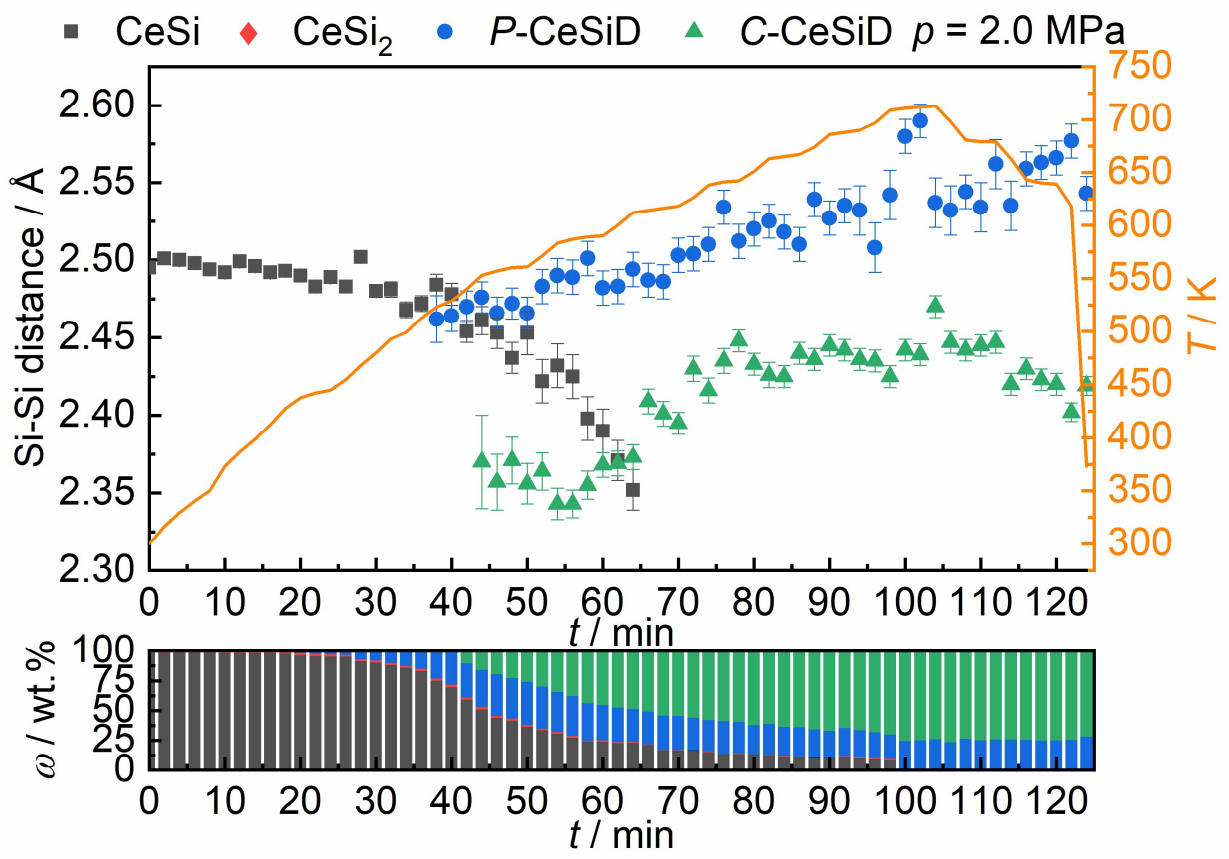

Figure S6: Refined Si - Si intrachain distances of CeSi, *P*- and *C*-CeSiD during the temperature dependent part of the *in situ* deuteration of CeSi, the bottom part shows the phase fractions at corresponding times; errors are given as ±*σ* or smaller than the symbols.

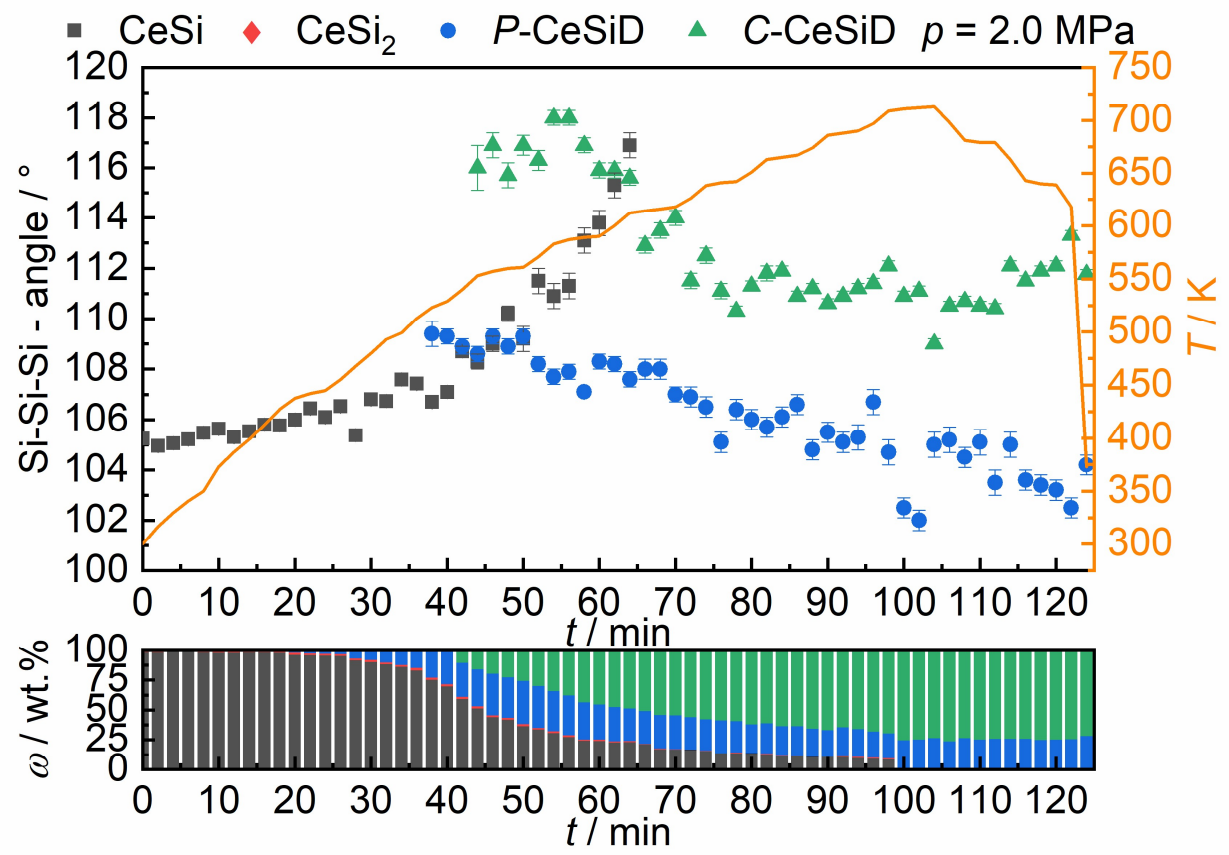

Figure S7: Refined Si – Si – Si angles of CeSi, *P*- and *C*-CeSiD during the temperature dependent part of the *in situ* deuteration of CeSi. Included were all values for phases with refinable atomic positions, the bottom part shows the phase fractions at corresponding times; errors are given as ±*σ* or smaller than the symbols.

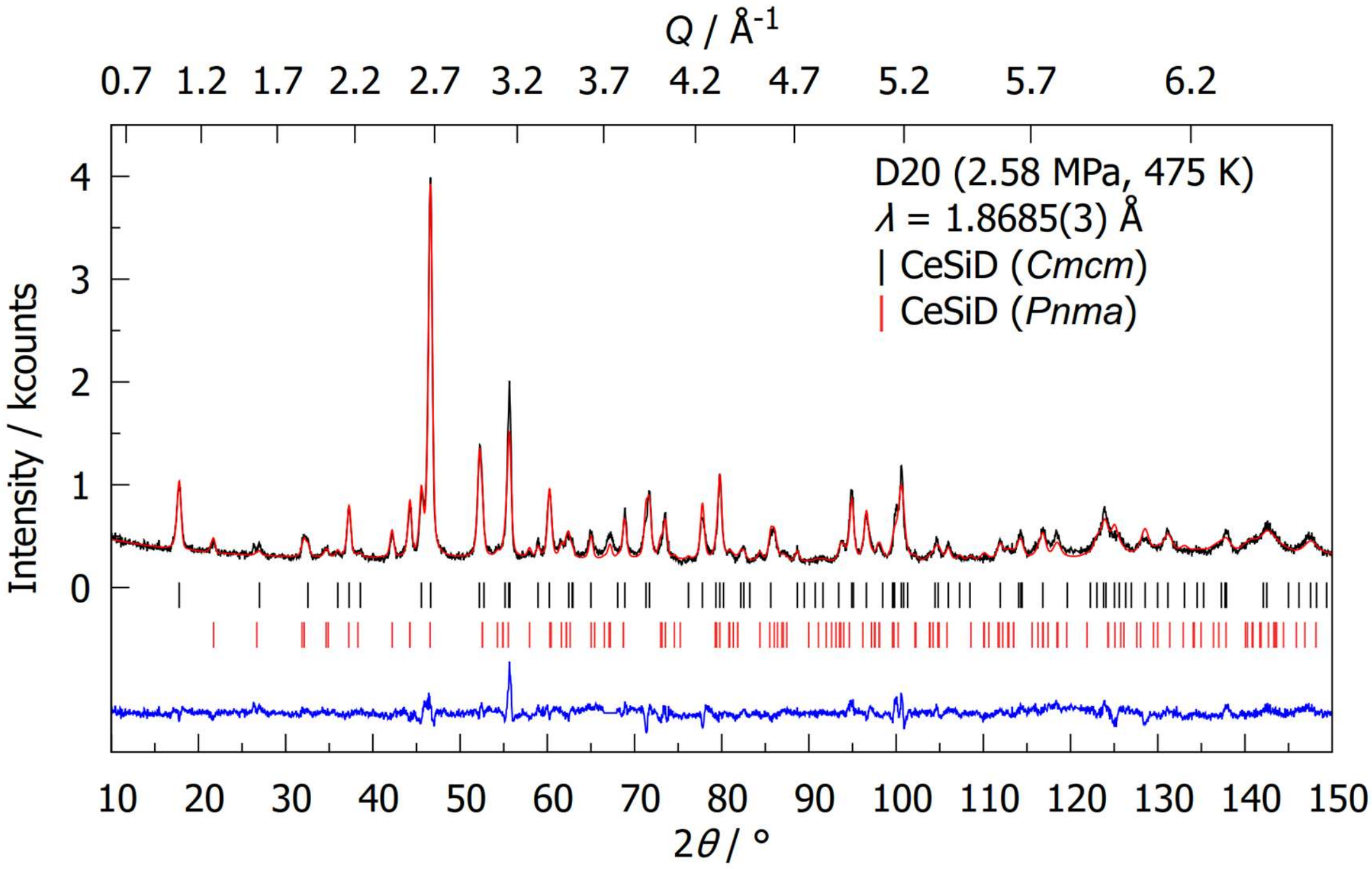

Figure S8: Rietveld refinement on the neutron powder diffraction pattern of deuterated CeSi at 475 K / 2.58 MPa, taken at D20 (Grenoble, France) at $\lambda = 1.8685(3)$ Å. *C*-CeSiD: 69(2) %, *P*-CeSiD: 31(1) %, $R_p = 6.96$ %, $R_{wp} = 8.82$ %, $\chi^2 = 2.79$. The *sof* of D1 in both phases was fixed at 1. This refinement was used as the basis for the following sequential refinements. NUMOR: 244457.[29]

Table S3: Crystal structure of *P*-CeSiD and selected interatomic distances determined by neutron powder diffraction at 475 K / 2.58 MPa, taken at D20 (Grenoble, France) (Figure S8), NUMOR: 244457.[29]

CeSiD (*Pnma*)
$a = 8.1124(7)$ Å, $b = 4.0158(3)$ Å, $c = 6.2870(6)$ Å, $V = 204.82(3)$ Å^3

	Wyckoff site	*x*	*y*	*z*	B_{iso} / Å^2	*sof*
Ce	4*c*	0.177(1)	¼	0.131(2)	0.2(2)	1
Si	4*c*	0.019(2)	¼	0.614(2)	1.4(3)	1
D	4*c*	0.379(1)	¼	0.422(1)	2.3(2)	1
Ce - Ce			3.87(1) Å / 3.92(1) Å / 4.0158(3) Å / 4.32(1) Å			
Ce - D				2.44(1) Å / 2.442(7) Å / 2.46(1) Å		
Si - Si						2.49(1) Å
Si - Si - Si						107.7(3)° @ 2.49(1) Å

Table S4: Crystal structure of *C*-CeSiD and selected interatomic distances determined by neutron powder diffraction at 475 K / 2.58 MPa, taken at D20 (Grenoble, France) (Figure S8), NUMOR: 244457.[29]

CeSiD (*Cmcm*)
a = 4.25140(2) Å, b = 12.1213(6) Å, c = 4.0040(2) Å, V = 206.33(2) Å^3

	Wyckoff site	x	y	z	B_{iso} / Å^2	*sof*
Ce	4*c*	0	0.3546(4)	¼	B_{iso}(Ce, *P*-CeSiD)	1
Si	4*c*	0	0.0562(6)	¼	B_{iso}(Si, *P*-CeSiD)	1
D	4*c*	0	0.7566(4)	¼	B_{iso}(D, *P*-CeSiD)	1
Ce - Ce			3.867(4) Å / 4.0040(2) Å / 4.054(6) Å / 4.25140(2) Å			
Ce - D						2.413(4) Å / 2.435(3) Å
Si - Si						2.422(6) Å
Si - Si - Si						111.5(2)° @ 2.422(6) Å

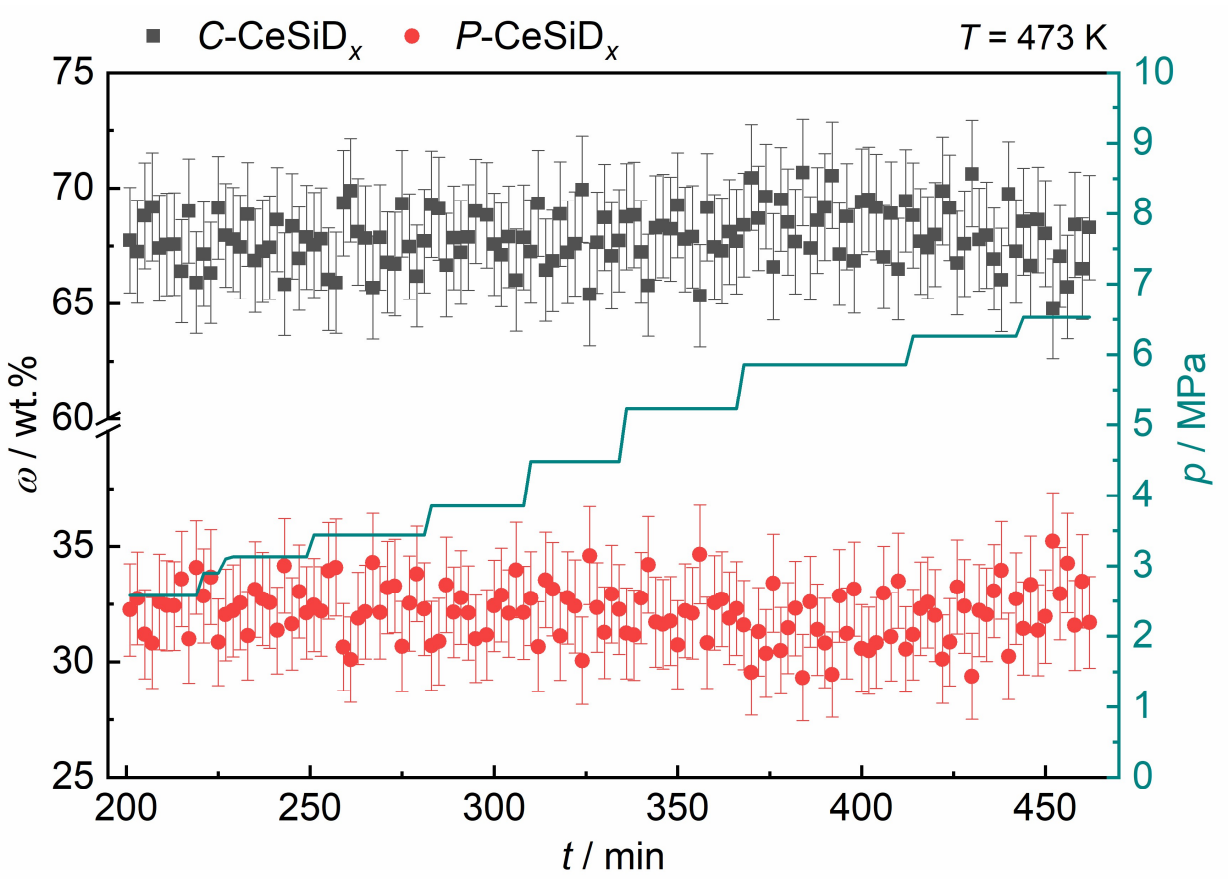

Figure S9: Mass fractions of *C*- and *P*-CeSiD$_x$ during the pressure dependent part of the *in situ* deuteration of CeSi.

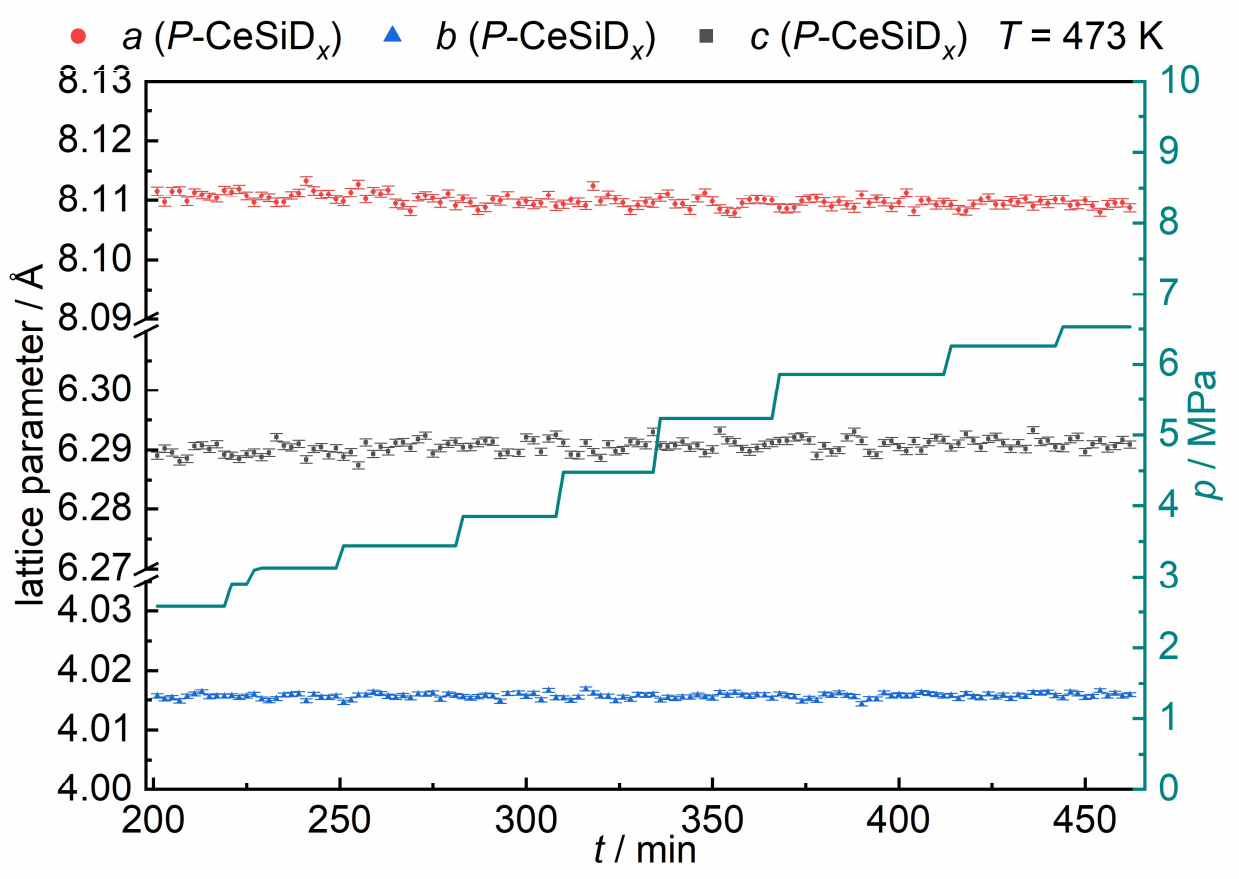

Figure S10: Lattice parameters of *P*-$CeSiD_x$ during the pressure dependent part of the *in situ* deuteration of CeSi; errors are given as $\pm\sigma$ or smaller than the symbols.

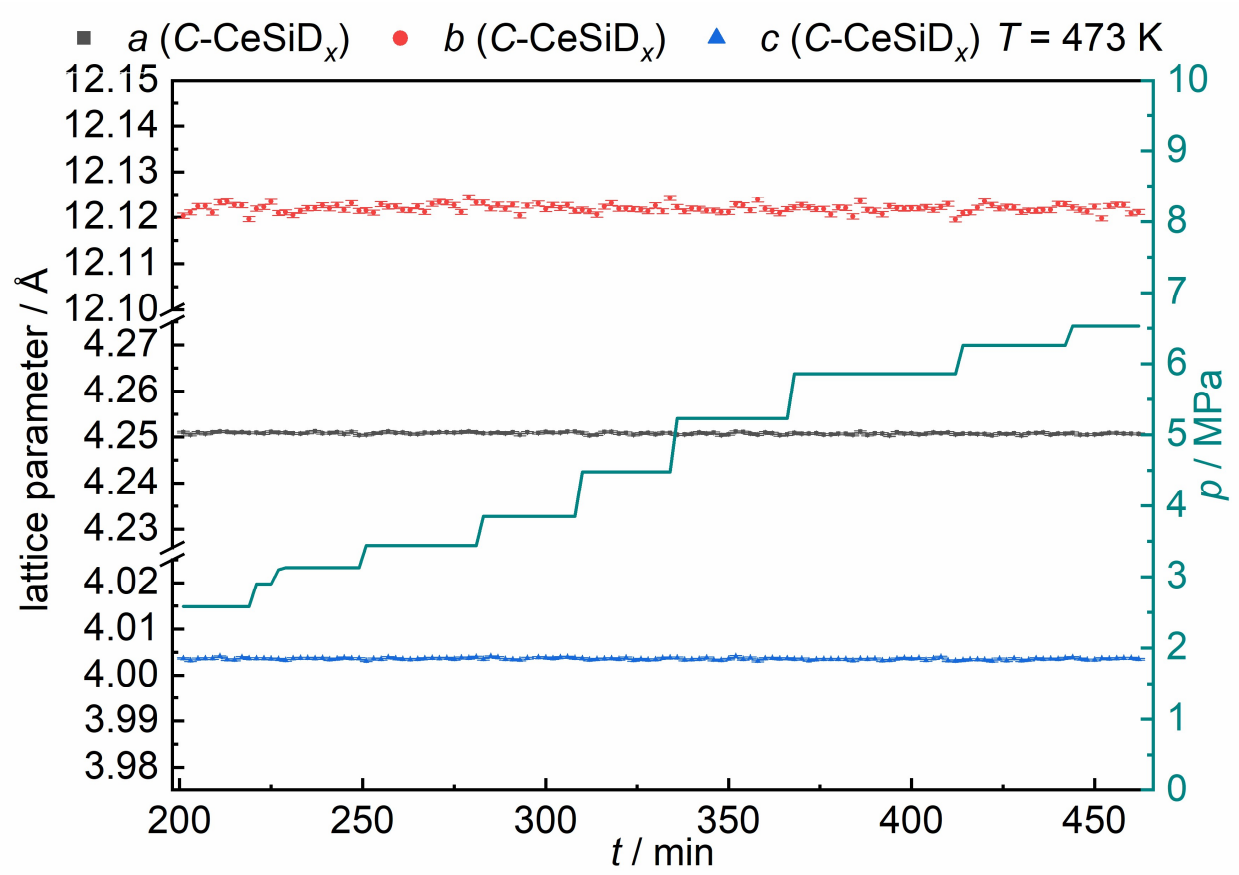

Figure S11: Lattice parameters of *C*-$CeSiD_x$ during the pressure dependent part of the *in situ* deuteration of CeSi; errors are given as $\pm\sigma$ or smaller than the symbols.

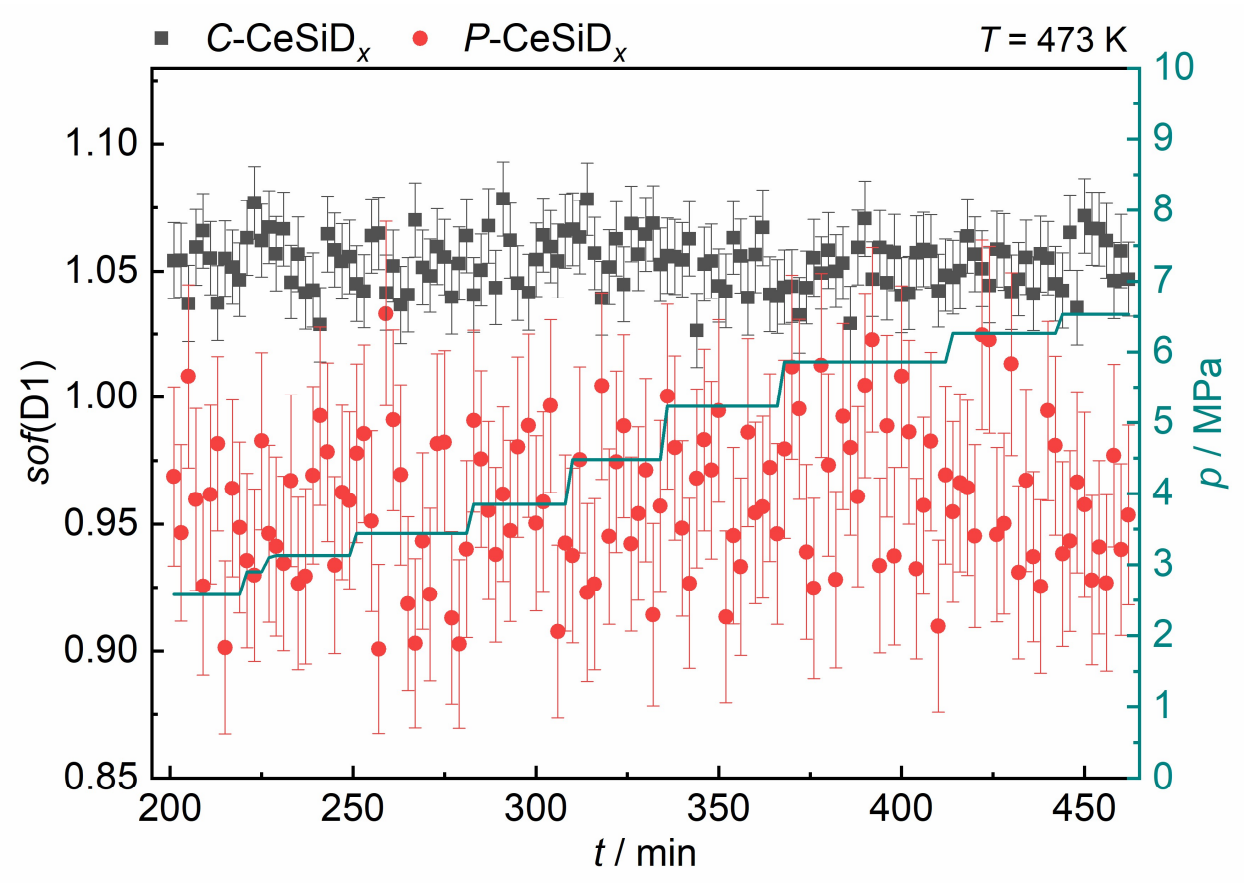

Figure S12: Site occupation factors of D1 in *C*- and *P*-$CeSiD_x$ during the pressure dependent part of the *in situ* deuteration of CeSi. (the *sof* was allowed to reach values beyond the physical limit of 1).

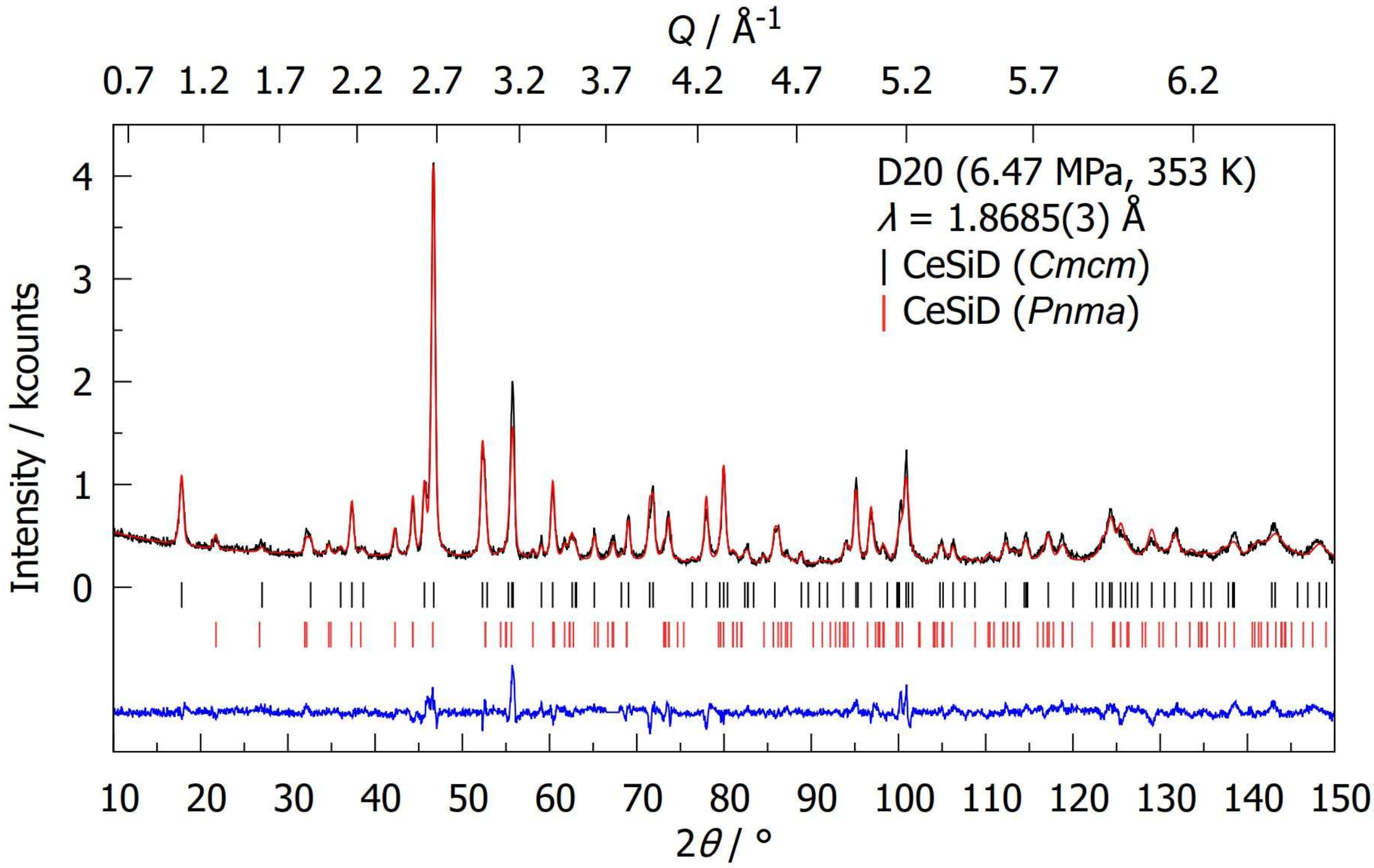

Figure S13: Rietveld refinement on the neutron powder diffraction pattern of deuterated CeSi at 353 K / 6.47 MPa, taken at D20 (Grenoble, France) at $\lambda = 1.8685(3)$ Å. *C*-CeSiD: 71(2) %, $R_{Bragg} = 6.96$ %, *P*-CeSiD: 29(1) %, $R_{Bragg} = 9.79$ %, $R_p = 6.94$ %, $R_{wp} = 8.83$ %, $\chi^2 = 2.76$. The *sof* of D1 in both phases was fixed at 1. NUMOR: 244594.[29]

Table S5: Crystal structure of *P*-CeSiD and selected interatomic distances determined by neutron powder diffraction at 353 K / 6.47 MPa, taken at D20 (Grenoble, France) (Figure S13), NUMOR: 244594.[29]

CeSiD (*Pnma*)
a = 8.0946(7) Å, *b* = 4.0095(3) Å, *c* = 6.2791(5) Å, *V* = 203.79(3) Å³

	Wyckoff site	*x*	*y*	*z*	B_{iso} / Å²	*sof*
Ce	4*c*	0.177(1)	¼	0.126(2)	0.1(2)	1
Si	4*c*	0.02(2)	¼	0.615(2)	0.9(2)	1
D	4*c*	0.377(1)	¼	0.424(1)	1.7(2)	1
Ce - Ce	3.84(1) Å / 3.91(1) Å / 4.0095(3) Å / 4.34(1) Å					
Ce - D	2.410(7) Å / 2.45(1) Å / 2.48(1) Å					
Si - Si	2.49(1) Å					
Si - Si - Si	107.2(3)° @ 2.49(1) Å					

Table S6: Crystal structure of *C*-CeSiD and selected interatomic distances determined by neutron powder diffraction at 353 K / 6.47 MPa, taken at D20 (Grenoble, France) (Figure S13), NUMOR: 244594.[29]

CeSiD (*Cmcm*)
a = 4.2439(2) Å, *b* = 12.0966(5) Å, *c* = 3.9956(2) Å, *V* = 205.12(1) Å³

	Wyckoff site	*x*	*y*	*z*	B_{iso} / Å²	*sof*
Ce	4*c*	0	0.3555(4)	¼	B_{iso}(Ce, *P*-CeSiD)	1
Si	4*c*	0	0.0544(6)	¼	B_{iso}(Si, *P*-CeSiD)	1
D	4*c*	0	0.7576(3)	¼	B_{iso}(D, *P*-CeSiD)	1
Ce - Ce	3.874(5) Å / 3.9956(2) Å / 4.027(6) Å / 4.2439(2) Å					
Ce - D	2.421(3) Å 2.430(3) Å					
Si - Si	2.392(6) Å					
Si - Si - Si	113.3(2)° @ 2.392(6) Å					

SI 4. Thermal analysis of the hydrogenation of CeSi

Differential scanning calorimetry with a hydrogen pressure of 2.4 MPa showed an exothermic signal on the first cycle, and no further thermal signals on cooling or the subsequent cycles (Figure S4). The reaction product was confirmed to be a mixture of CeSi and the two hydrides by X-ray powder diffraction (Figure S5). This behavior is in line with the previously investigated *LnTt* phases (*Ln* = La, Nd; *Tt* = Si, Ge, Sn) which, (apart from LaSi) all underwent a detectable exothermic hydrogenation reaction.[17] The existence of CeSi residues after a second cycle without signals might be explained by a number of factors like particle size, a partial dehydrogenation between the DSC and XRPD measurement or insufficient hydrogen diffusion through the pan.

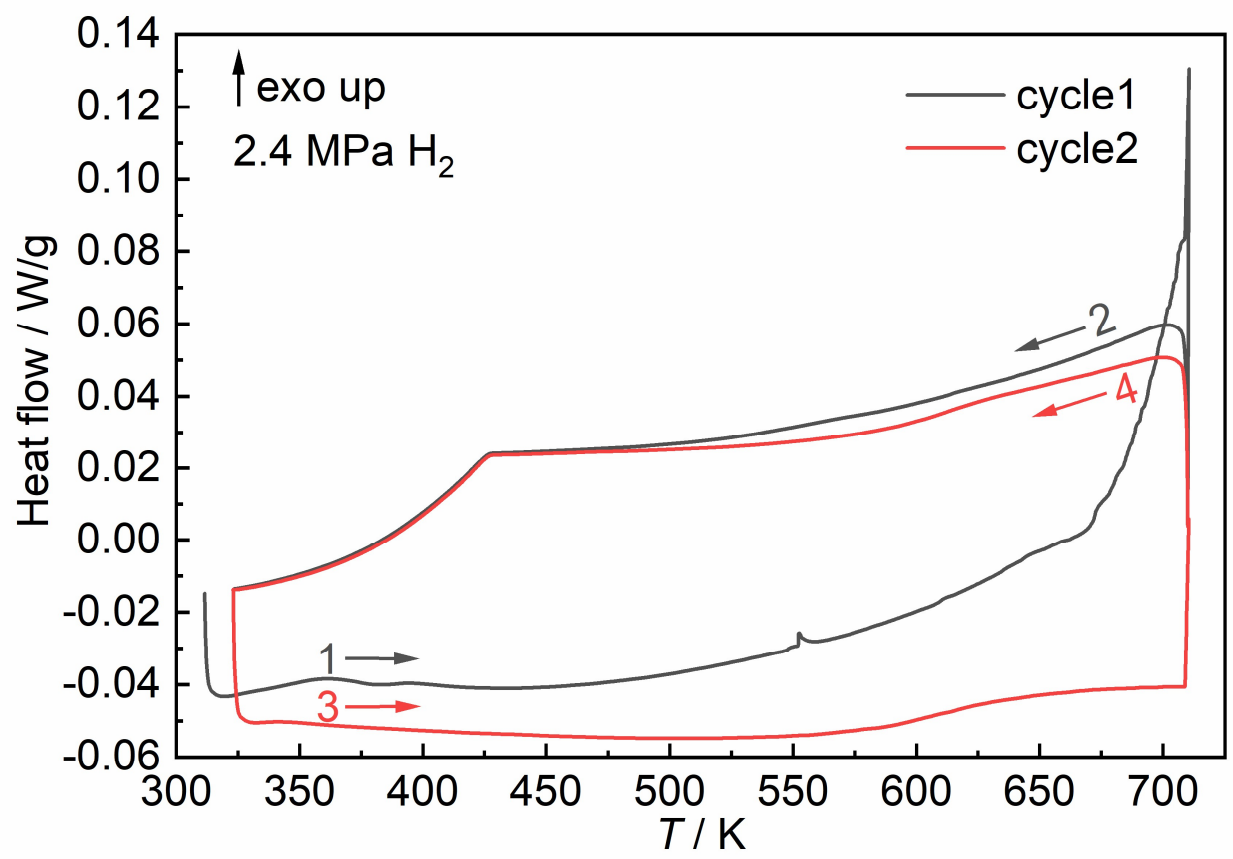

Figure S14: Differential scanning calorimetry measurement of 20.9 mg of CeSi under 2.4 MPa H_2 pressure. All 4 heating/cooling segments were conducted with rates of +/-10 K/min. The maximum temperature of 710 K was kept for 60 min in both cycles. The sudden change in heat flow while cooling at 425 K is caused by the cooling cycle of the device.

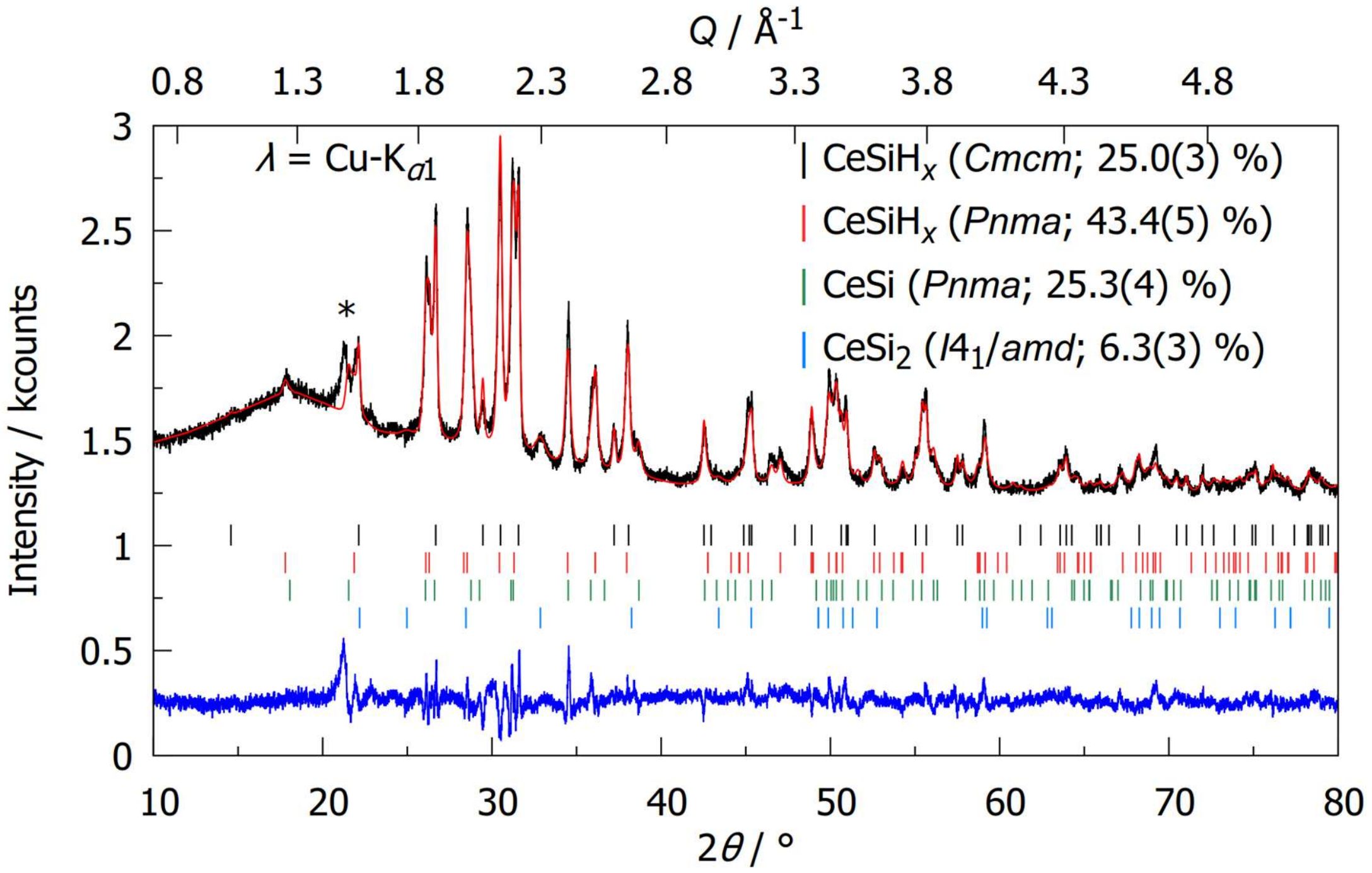

Figure S15: Rietveld refinement on the X-ray powder diffraction pattern of hydrogenated CeSi, Huber G670, *C*-$CeSiH_x$: 25.0(3) w.-%, *P*-$CeSiH_x$: 43.4(5) w.-%, CeSi: 25.3(4) w.-%, $CeSi_2$: 6.3(3) w.-%, $R_p = 1.73$ %, $R_{wp} = 2.37$ %, $\chi^2 = 0.829$, * marks a reflection causes by Lithelen grease.

SI 5. Parameters of the Birch–Murnaghan fit of CeSiH

Table S7: Parameters of the Birch–Murnaghan fit of CeSiH.

	P-CeSiH	*C*-CeSiH
E_0 (kJ mol^{-1})	5.11(3)	0.022(12)
V_0 (cm^3 mol^{-1})	50.727(9)	51.0796(4)
B_0 (GPa)	45.57(8)	44.68(4)
B_1 (-)	5.05(6)	4.47(3)

SI 6. Magnetic properties of $CeSiD_x$

As magnetism can be sensitive to even small structural changes, the magnetic behavior of $CeSiD_x$ was also studied. Above 150 K it shows Curie-Weiss behavior with an experimental magnetic moment of 2.86(2) μ_B Ce atom^{-1} (Figure S16a). This value is in fair agreement with the value of 2.54 μ_B for a free Ce^{3+} ion.[55] We can thus safely assume stable trivalent ground states for cerium, in line with $CeSiH_x$. The sample also shows small deviations from Curie-Weiss behavior in going to lower temperatures. This might be attributed to small amounts of impurity phases.

An additional measurement in ZFC-FC mode was conducted at a much lower external field of 100 Oe (Figure S16b) in order to determine the precise ordering temperatures. The sample shows two minima in the $d\chi/dT$ derivatives. With a T_C of 24.6(1) K and a wiggly trace below, that can probably be attributed to domains with different deuterium content. The magnetization behavior of $CeSiD_x$ is finally summarized in Figure S16c. The isotherm at 50 K, i.e., well above the magnetic ordering temperatures shows a monotonous increase as usually observed for paramagnetic materials. The 10 respectively 3 K isotherms show an extremely steep increase already at very small field strengths and then tend to saturation. Given the very small hysteresis, $CeSiD_x$ is a very soft ferromagnet. The saturation magnetization at 3 K and 90 kOe is 1.77(1) μ_B Ce atom^{-1}, much smaller than the theoretical saturation magnetization of 2.14 μ_B Ce atom^{-1} according to $g_J \times J$.[55] Considering again the argument of a randomly oriented powder the magnetization is (slightly) higher than expected, indicating potential preferred orientation in the field or influences from the impurities. A further reason might be crystal field splitting of $J = 5/2$ ground state of Ce^{3+}.

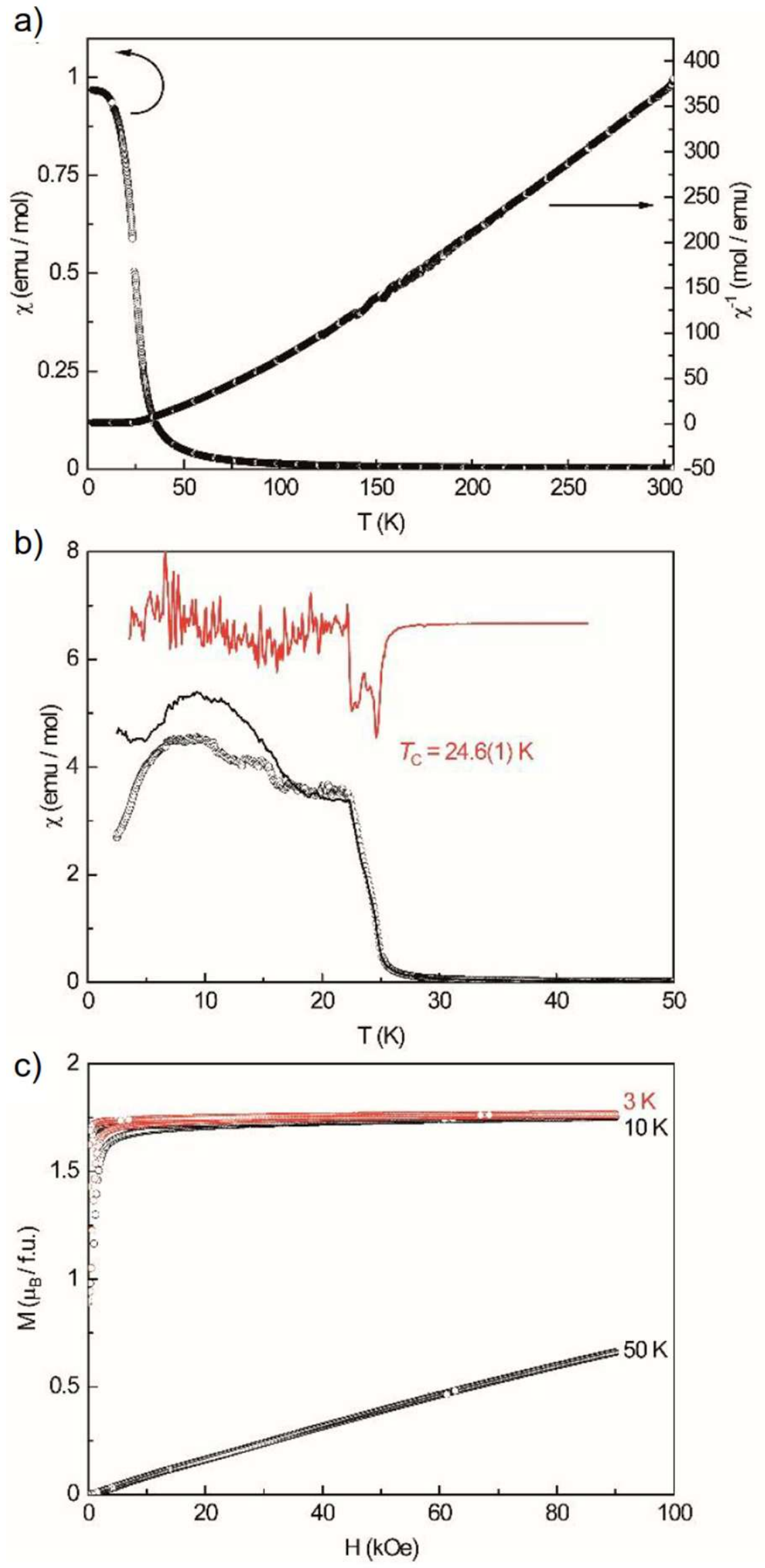

Figure S16: Temperature dependence of the magnetic susceptibility of CeSiD$_x$ measured at a) 10 kOe zero-field-cooling (ZFC) and b) at 100 Oe in zero-field-cooling / field-cooling (ZFC/FC) mode. The derivative dχ/dT of the field-cooling curve in shown in red, emphasizing the two Curie temperatures. c) Magnetization isotherms recorded at 3 (red), 10 and 50 K (black).

SI 7. Magnetic reflections of $CeSiD_x$ and second order phase transition fits

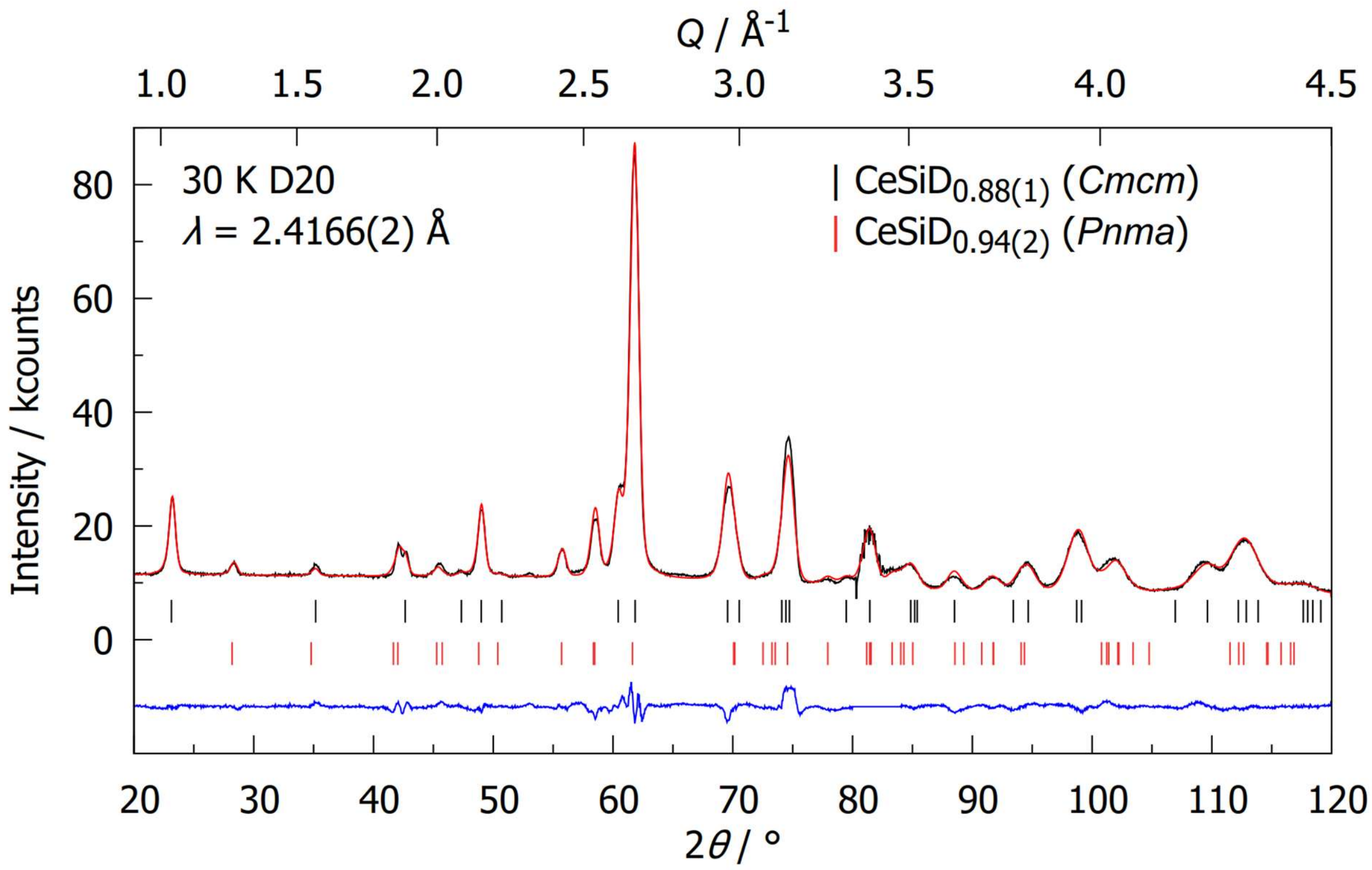

Figure S17 (also as Figure 15 in the main text): Rietveld refinement of the crystal structures of *C*-$CeSiD_{0.88(1)}$ (71(2) w.-%) and *P*-$CeSiD_{0.94(2)}$ (29(1) w.-%) on neutron powder diffraction pattern at 30 K, taken at D20 (Grenoble, France) at $\lambda = 2.4166(2)$ Å, $R_p = 1.48$ %, $R_{wp} = 3.40$ %, $\chi^2 = 14.9$, NUMOR: 256734.[27]

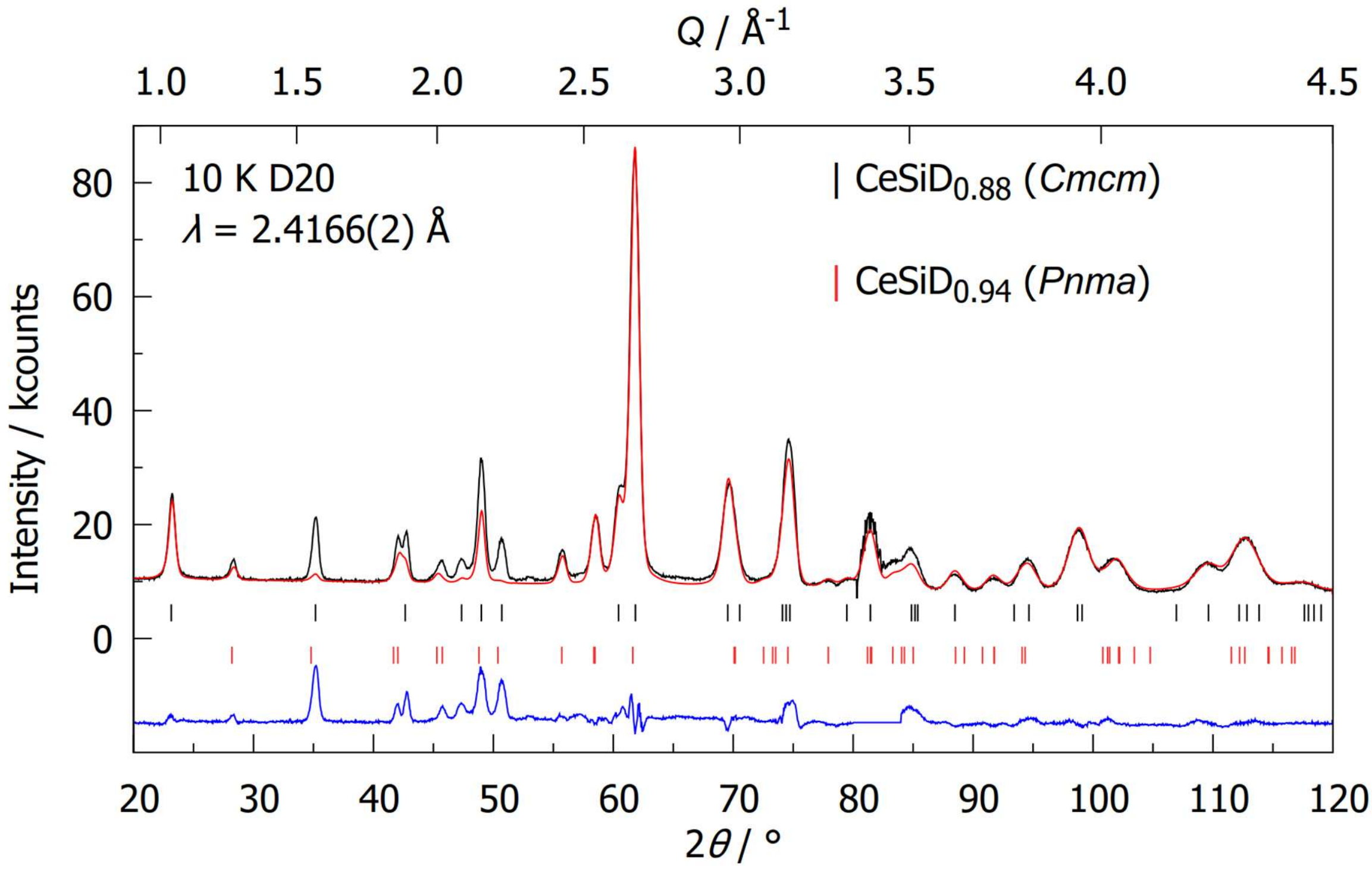

Figure S18: Rietveld refinement on the neutron powder diffraction pattern of deuterated CeSi at 10 K, taken at D20 (Grenoble, France) at λ = 2.41 Å. All parameters except for zero-shift and lattice parameters were taken from the refinement in **Figure S17** and fixed, NUMOR: 256666.[27]

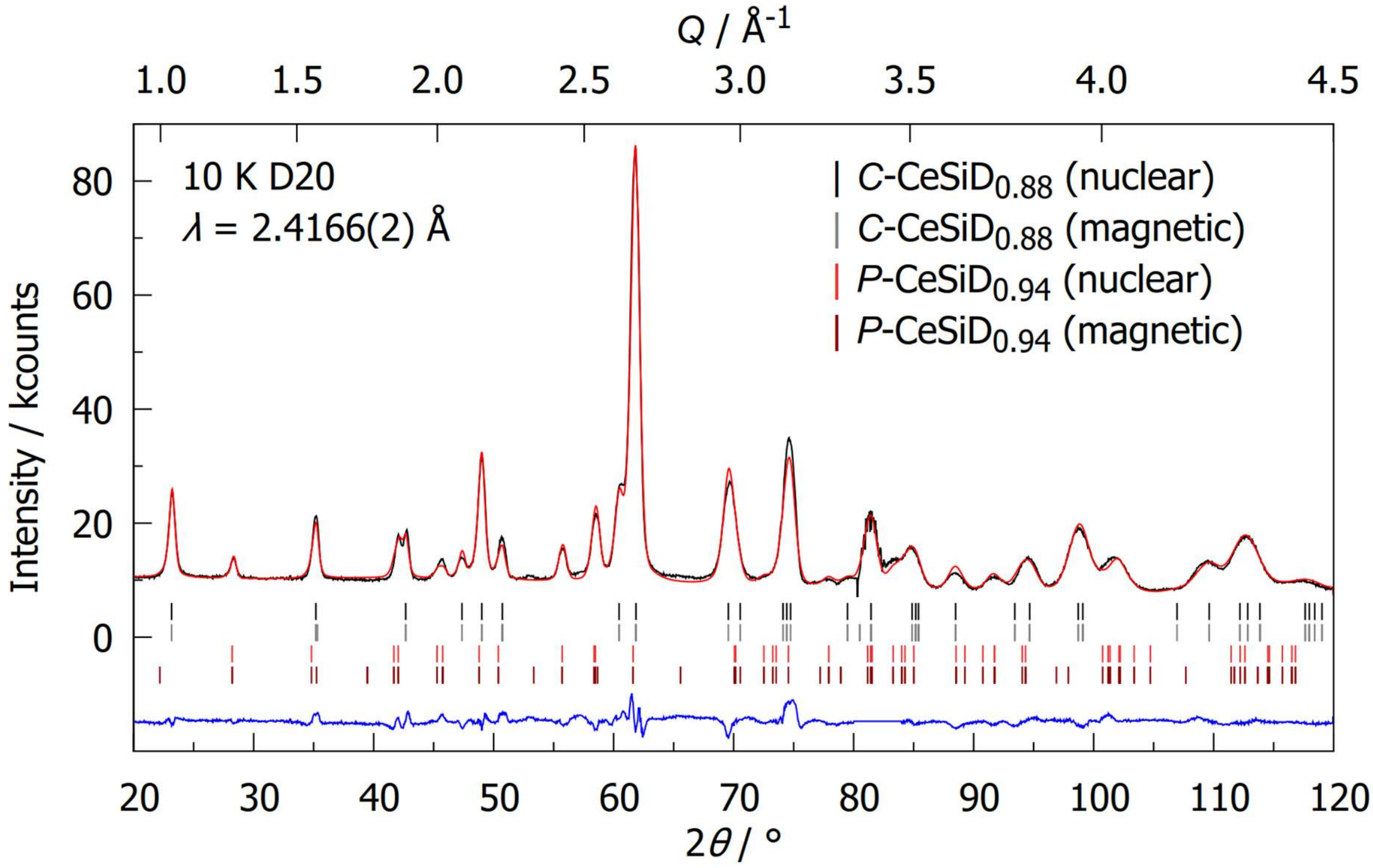

Figure S19 (also as Figure 16 in the main text): Rietveld refinement of the nuclear and magnetic structures of *C*-$CeSiD_{0.88}$ (71 w.-%) and *P*-$CeSiD_{0.94}$ (29 w.-%) on neutron powder diffraction pattern at 10 K, taken at D20 (Grenoble, France) at λ = 2.4166(2) Å, R_p = 3.28 %, R_{wp} = 4.24 %, χ^2 = 22.8, μ(Ce^{3+}, *C*-phase) = 1.89(2) μ_B, $R_{mag(C\text{-phase})}$ = 7.46 %, μ(Ce^{3+}, *P*-phase) = 1.77(6) μ_B, $R_{mag(P\text{-phase})}$ = 9.22 %, NUMOR: 256666.[27]

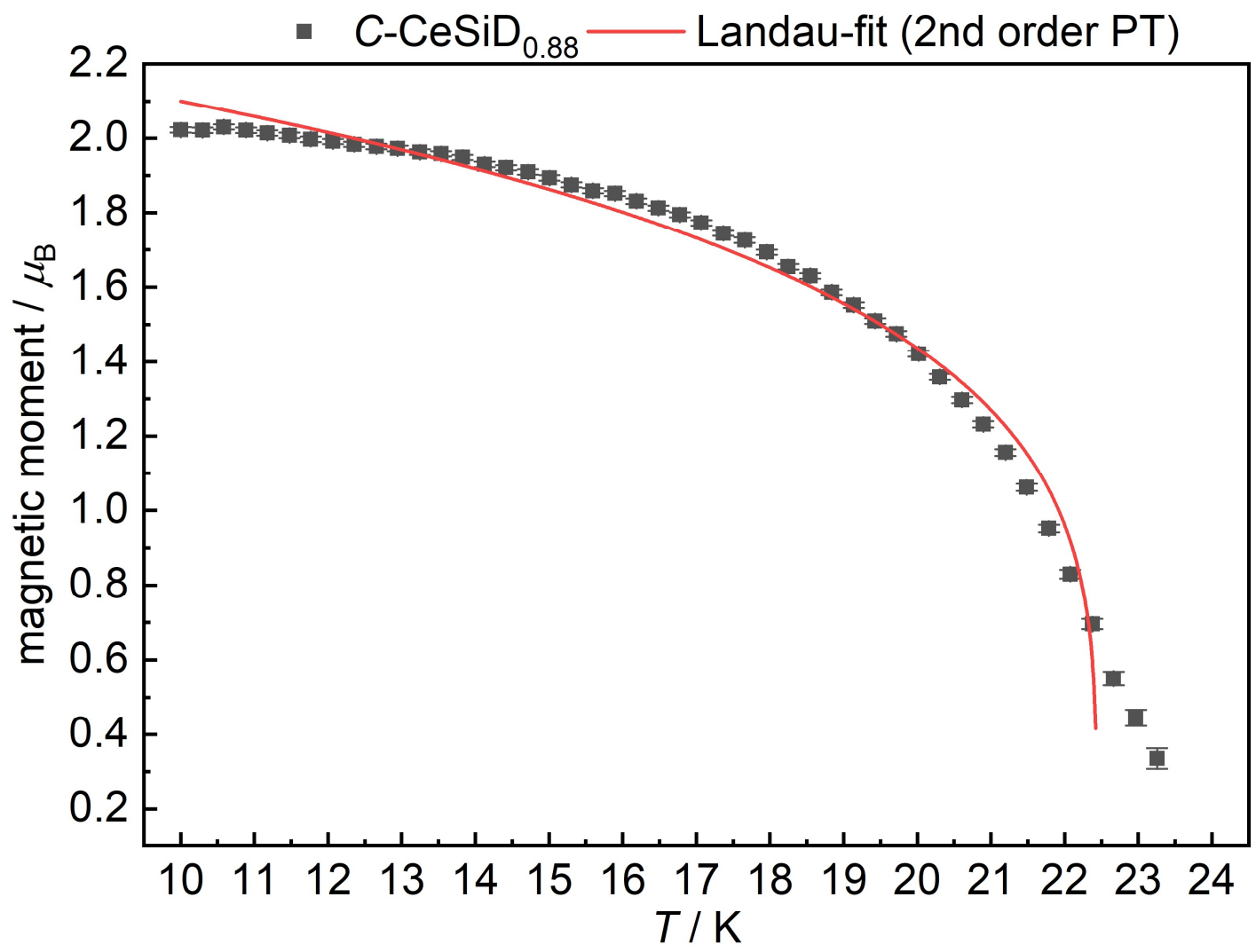

Figure S20: Refined magnetic moments *m* of *C*-CeSiD_x from the refinement of the difference between the measurement at the respective temperature and one at 50K. The second order phase transition was fitted with eq. (2); errors are given as $\pm\sigma$ or smaller than the symbols.

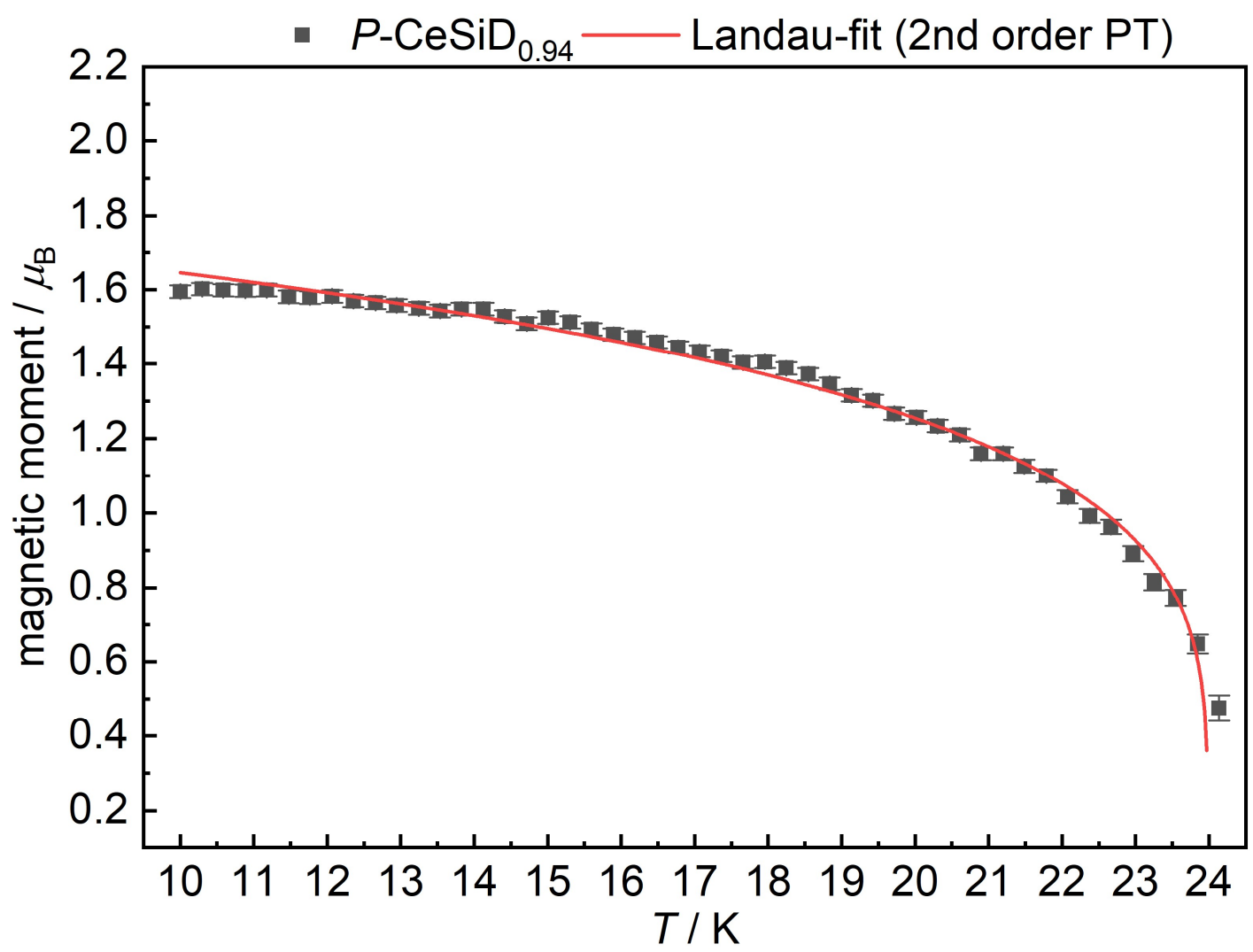

Figure S21: Refined magnetic moments *m* of *P*-CeSiD_x from the refinement of the difference between the measurement at the respective temperature and one at 50K. The second order phase transition was fitted with eq. (2), errors are given as $\pm\sigma$ or smaller than the symbols.

Table S8: Fitted values of the coefficients of equation (2) for the magnetic contributions of *C*-$CeSiD_{0.88}$ and *P*-$CeSiD_{0.94}$.

	$CeSiD_{0.88}$ (*Cmcm*)	$CeSiD_{0.94}$ (*Pnma*)
A / μ_B	2.41(3)	1.85(2)
T_C / K	22.43(8)	24.0(1)
β	0.23(1)	0.22(1)
Red. χ^2	65.18	5.85
Adj. R^2	0.96	0.97

SI 8. Magnetic refinement of $CeSiD_x$ at 1.6 K

In order to synthesize a *P*-phase rich sample, a second sample of $CeSiD_x$ (separate from the one reported on in the main text) was prepared by putting CeSi in an autoclave under 8.5 MPa D_2 pressure and heating to 575 K for 24h.

The sample was measured at Diffractometer D2b (ILL, Grenoble) in experiment 5-24-573 with temperatures of 50 K (NUMORS: 588141 – 588158), 13 K (NUMORS: 588159 – 588176) and 1.6 K (NUMORS: 588177 – 588194) The raw data is available at.[25]

Due to the lower reaction temperature, the sample contains a higher amount (59(1) %) of *P*-rather than *C*-phase (41(1) %) (see Figure S17). The *C*-phase (C-$CeSiD_{0.98(1)}$), featuring a higher deuterium content than the *P*-phase (P-$CeSiD_{0.94(1)}$) (see Tables S7, S8). Down to a temperature of 1.6 K (Figure S18) no signs of a phase transition or a change in magnetic behavior were observed (Table S9, S10). The refined values of $\mu(Ce^{3+}$, *C*-phase) = 1.88(6) μ_B and $\mu(Ce^{3+}$, *P*-phase) = 1.63(5) μ_B are not significantly different from those derived from the measurement at 10 K (Figure 17) within $\pm 2\sigma$.

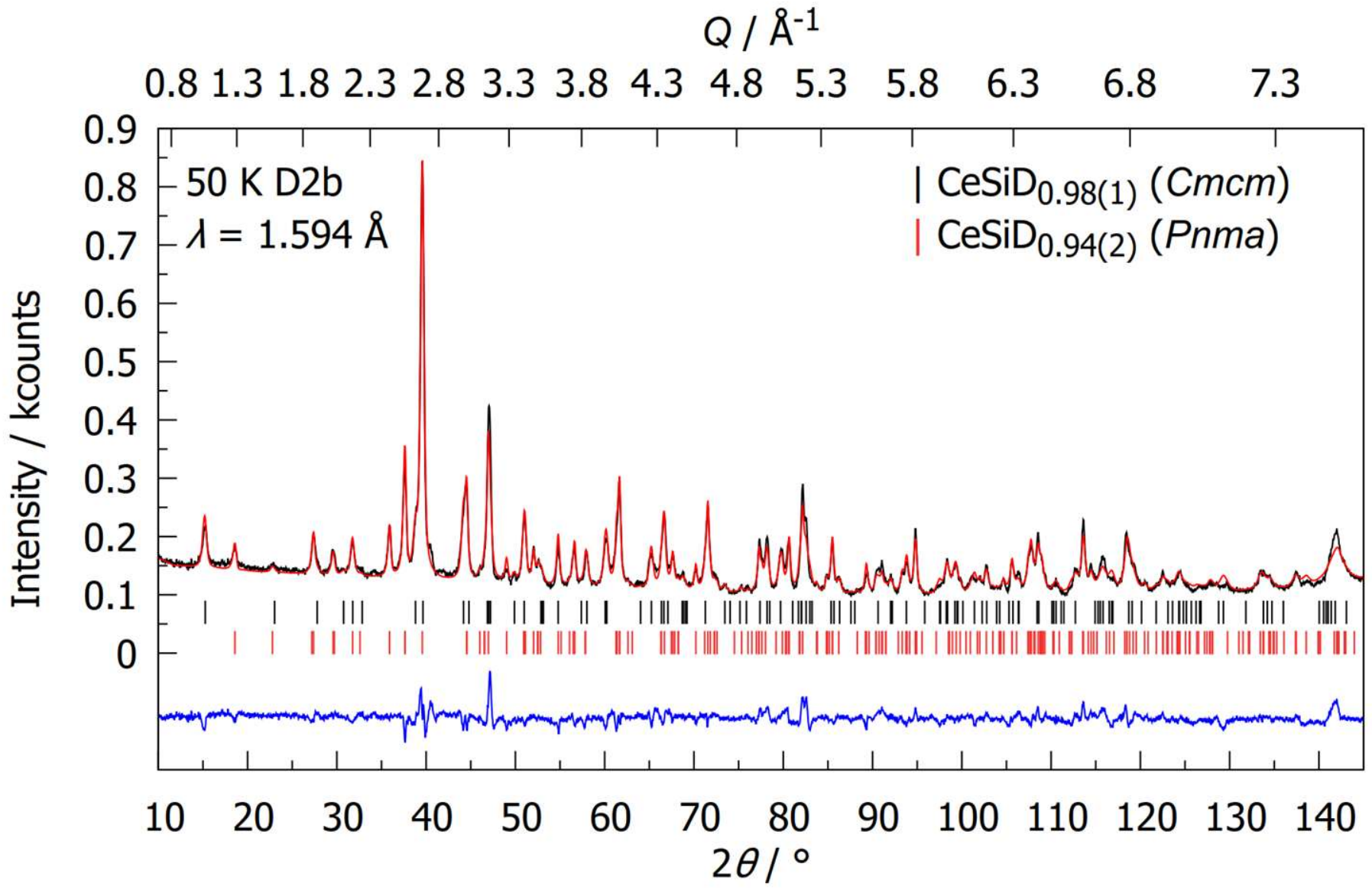

Figure S22: Rietveld refinement on the neutron powder diffraction pattern of $CeSiD_x$ at 50 K, taken at D2b (Grenoble, France) at $\lambda = 1.594$ Å. C-$CeSiD_{0.98(1)}$: 41(1) %, $R_{Bragg(C\text{-}CeSiD0.98)} = 9.77$ %, P-$CeSiD_{0.94(2)}$: 59(1) %, $R_{Bragg(P\text{-}CeSiD0.94)} = 9.09$ %, $R_p = 3.83$ %, $R_{wp} = 5.00$ %, $\chi^2 = 6.51$, NUMOR: 588141-588158.[25]

Table S9: Crystal structure data of P-$CeSiD_{0.94(2)}$ and selected interatomic distances determined by neutron powder diffraction at 50 K as refined from Sample B, NUMOR: 588141 – 588158.[25]

$CeSiD_{0.94(2)}$ (*Pnma*)
$a = 8.0825(3)$ Å, $b = 4.0024(2)$ Å, $c = 6.2527(3)$ Å, $V = 202.27(1)$ Å^3

	x	*y*	*z*	B_{iso} / Å^2	*sof*
Ce	0.1737(6)	¼	0.1227(8)	0.65(7)	1
Si	0.0274(9)	¼	0.6035(9)	0.50(7)	1
D	0.3805(6)	¼	0.4162(7)	1.88(8)	0.94(2)
Ce - Ce		3.774(6) Å / 3.912(6) Å / 4.0024(2) Å / 4.343(7) Å			
Ce - D			2.382(7) Å / 2.422(4) Å / 2.482(7) Å		
Si - Si					2.424(5) Å

Table S10: Crystal structure data of C-CeSiD$_{0.98(1)}$ and selected interatomic distances determined by neutron powder diffraction at 50 K as refined from Sample B, NUMOR: 588141 – 588158.[25]

CeSiD$_{0.98(1)}$ (*Cmcm*)
a = 4.2410(3) Å, b = 12.0354(7) Å, c = 3.9842(2) Å, V = 203.36(2) Å^3

	x	y	z	B_{iso} / Å^2	*sof*
Ce	0	0.3505(4)	¼	B_{iso} (Ce, P-CeSiD$_{0.94}$)	1
Si	0	0.0605(6)	¼	B_{iso} (Si, P-CeSiD$_{0.94}$)	1
D	0	0.7541(5)	¼	B_{iso} (D, P-CeSiD$_{0.94}$)	0.976(8)
Ce - Ce				3.784(7) Å / 3.9842(3) Å / 4.113(9) Å / 4.2410(4) Å	
Ce - D				2.357(5) Å / 2.417(4) Å	
Si - Si				2.468(9) Å	

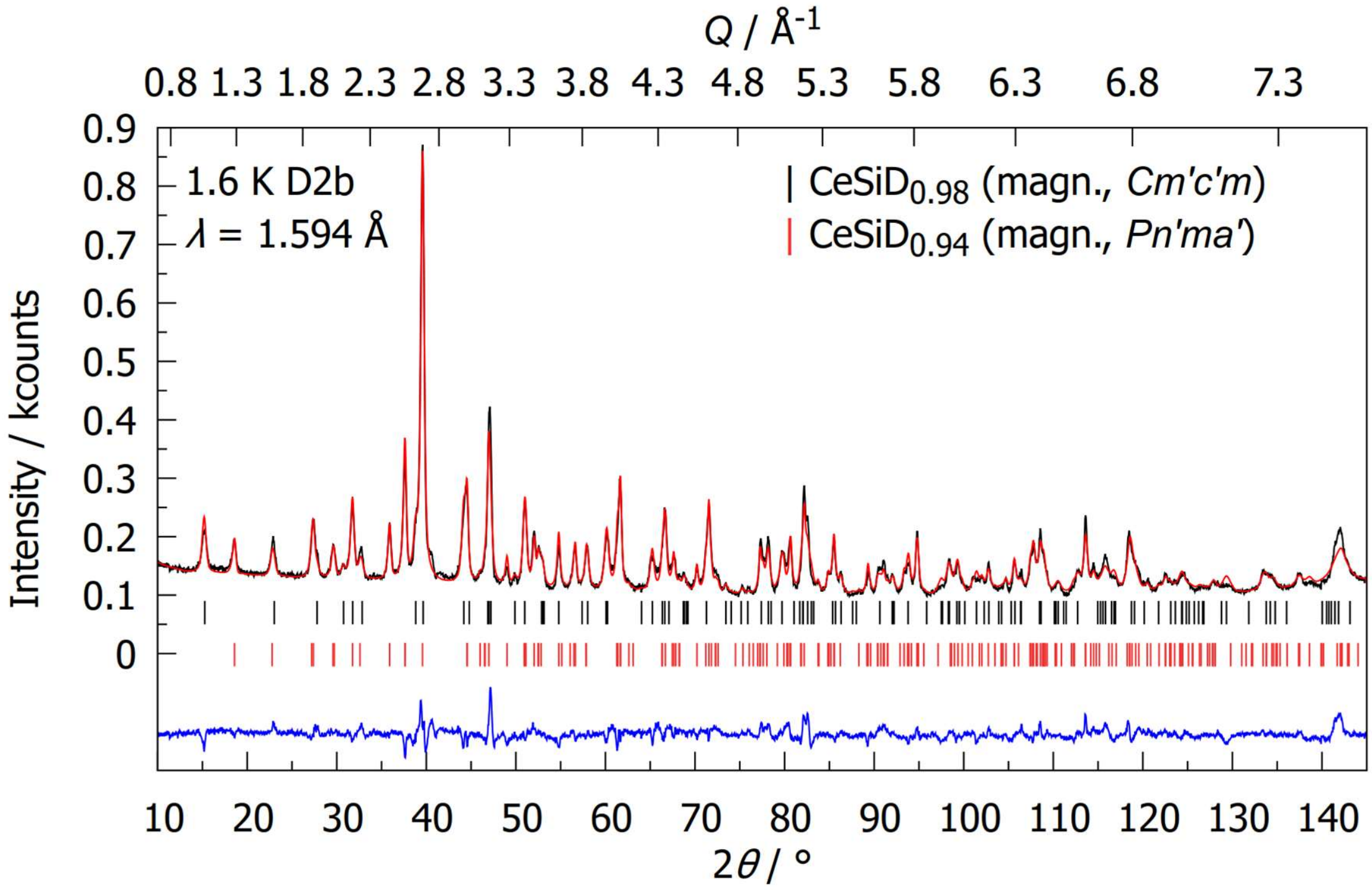

Figure S23: Rietveld refinement on the neutron powder diffraction pattern of sample B at 1.6 K, taken at D2b (Grenoble, France) at λ = 1.594 Å, scale factors and deuterium contents were taken from a refinement at 50 K (Figure S17), R_p = 3.95 %, R_{wp} = 5.18 %, χ^2 = 6.96, $R_{Bragg(C\text{-CeSiD0.98})}$ = 8.01 %, $m_{(C1)}$ = 1.88(6) μ_B, $R_{Bragg(P\text{-CeSiD0.94})}$ = 8.01 %, $R_{mag(C1)}$ = 11.7 %, $m_{(P1)}$ = 1.63(5) μ_B, $R_{mag(P1)}$ = 10.5 %, NUMOR: 588177-588194.[25]

Table S11: Crystal structure data of P-$CeSiD_{0.94(1)}$ and selected interatomic distances determined by neutron powder diffraction at 1.6 K as refined from Sample B, NUMOR: 588195 – 588212.[25]

$CeSiD_{0.94(1)}$ (*Pnma*) a = 8.0830(2) Å, b = 4.0015(1) Å, c = 6.2521(2) Å, V = 202.22(1) Å^3					
	x	y	z	B_{iso} / Å^2	*sof*
Ce	0.1735(5)	¼	0.1245(7)	0.61(6)	1
Si	0.0275(9)	¼	0.6016(9)	0.51(6)	1
D	0.3806(6)	¼	0.4159(7)	1.85(6)	0.936 (fix)
Ce - Ce	3.781(5) Å / 3.912(5) Å / 4.0015(1) Å / 4.335(6) Å				
Ce - D	2.381(6) Å / 2.428(4) Å / 2.474(6) Å				
Si - Si	2.411(5) Å				

Table S12: Crystal structure data of C-$CeSiD_{0.96(1)}$ and selected interatomic distances determined by neutron powder diffraction at 1.6 K as refined from Sample B, NUMOR: 588195 – 588212.[25]

$CeSiD_{0.98(1)}$ (*Cmcm*) a = 4.2415(2) Å, b = 12.0335(6) Å, c = 3.9834(2) Å, V = 203.31(2) Å^3					
	x	y	z	B_{iso} / Å^2	*sof*
Ce	0	0.3514(4)	¼	B_{iso}(Ce, P-$CeSiD_{0.94}$)	1
Si	0	0.0598(6)	¼	B_{iso}(Si, P-$CeSiD_{0.94}$)	1
D	0	0.7537(4)	¼	B_{iso}(D, P-$CeSiD_{0.94}$)	0.976 (fix)
Ce - Ce	3.797(4) Å / 3.9834(2) Å / 4.094(6) Å / 4.2415(3) Å				
Ce - D	2.359(4) Å / 2.425(3) Å				
Si - Si	2.457(6) Å				

The magnetic behavior of this $CeSiD_x$ sample was also studied. Above 150 K it shows Curie-Weiss behavior with an experimental magnetic moment of 2.40(2) μ_B Ce atom^{-1} (Figure S23a). This value is also in fair agreement with the value of 2.54 μ_B for a free Ce^{3+} ion.[55] The very small anomaly around 150 K is an artifact from a trace amount of an impurity phase. It is well known that trace amounts of iron in commercial cerium might affect the magnetic data. In the ZFC-FC measurement at an external field of 100 Oe (Figure S23b), the sample shows two minima in the $d\chi/dT$ derivatives, with a magnetic ordering happening at 24.7(1) K, similarly to the sample in the main text. As this sample contained a higher amount of P-phase and shows a more pronounced

signal at the higher T_C, it reinforces the assignment of T_C(*P*-phase) > T_C(*C*-phase). The saturation magnetization at 3 K and 90 kOe is and 1.40(1) μ_B Ce atom^{-1} (Figure S23c).

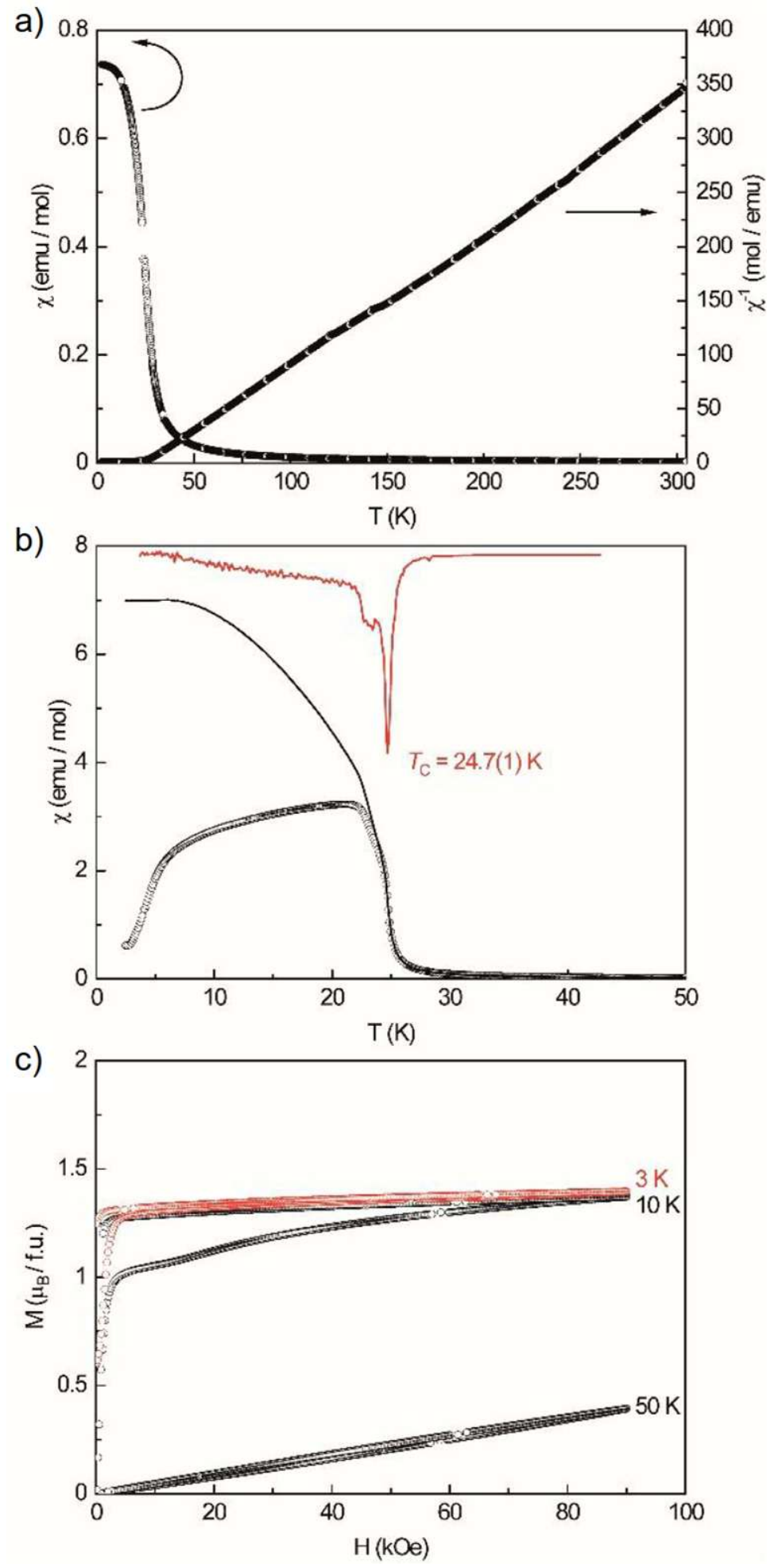

Figure S24: Temperature dependence of the magnetic susceptibility of the second $CeSiD_x$ sample, measured at a) 10 kOe zero-field-cooling (ZFC) and b) at 100 Oe in zero-field-cooling / field-cooling (ZFC/FC) mode. The derivative dχ/dT of the field-cooling curve in shown in red, emphasizing the two Curie temperatures. c) Magnetization isotherms recorded at 3 (red), 10 and 50 K (black).

SI 9. Table of refined structural Parameters at different Temperatures and Devices

Table S13: Structural parameters from CeSi and its hydrides (deuterides) as determined by theoretical calculations, X-ray and neutron powder diffraction. Dashed lines divide phases refined on the same measurements.

Fig.	Device	R_{wp} / %	Phase	R_{Bragg} / %	a / Å	b / Å	c / Å	V / Å^3	At.	x	y	z	d(Ce–Ce) / Å
T/K	type	χ^2	content / %										d(Si–Si) / Å
-	DFT	-	CeSi	-	8.25669	3.97122	5.98212	196.14865	Ce	0.17717	¼	0.88816	3.78057
0	calc.	-							Si	0.03299	¼	0.38514	2.47548
S1	D20	6.28	CeSi	8.07	8.3014(1)	3.96559(5)	5.96533(7)	196.379(4)	Ce	0.1797(1)	¼	0.1166(3)	3.767(2)
295	neut.	3.64	97.2(9)						Si	0.0340(2)	¼	0.6167(4)	2.488(2)
-	DFT	-	*P*-CeSiH	-	8.08839	4.02093	6.23183	202.67710	Ce	0.17530	¼	0.62254	3.79688
0	calc.	-							Si	0.02553	¼	0.10409	2.42807
									H	0.12224	¼	0.42116	
-	DFT	-	*C*-CeSiH	-	4.23677	12.09830	3.98432	204.22681	Ce	0	0.35447	¼	3.85309
0	calc.	-							Si	0	0.05538	¼	2.40097
									H	0	0.24343	¾	
S22	D2b	5.18	*P*-$CeSiD_{0.94(1)}$	8.55	8.0830(2)	4.0015(1)	6.2521(2)	202.22(1)	Ce	0.1735(5)	¼	0.1245(7)	3.781(5)
1.6	neutr.	6.96	59(1)						Si	0.0275(9)	¼	0.6016(9)	2.411(5)
									D	0.3806(6)	¼	0.4159(7)	
S22	D2b	5.18	*C*-$CeSiD_{0.98(1)}$	8.01	4.2415(2)	12.0335(6)	3.9834(2)	203.31(2)	Ce	0	0.3514(4)	¼	3.797(4)
1.6	neutr.	6.96	41(1)						Si	0	0.0598(6)	¼	2.457(6)
									D	0	0.7537(4)	¾	
S21	D2b	5.00	*P*-$CeSiD_{0.94(1)}$	9.09	8.0825(3)	4.0024(2)	6.2527(3)	202.27(1)	Ce	0.1737(6)	¼	0.1227(8)	3.774(6)
50	neut.	6.51	59(1)						Si	0.0274(9)	¼	0.6035(9)	2.424(5)
									D	0.3805(6)	¼	0.4162(7)	
S21	D2b	5.00	*C*-$CeSiD_{0.98(1)}$	9.77	4.2410(3)	12.0354(7)	3.9842(2)	203.36(2)	Ce	0	0.3505(4)	¼	3.784(4)
50	neut.	6.51	41(1)						Si	0	0.0605(6)	¼	2.468(9)
									D	0	0.7541(5)	¾	
2	D2b	4.32	*P*-$CeSiD_{0.82(2)}$	8.35	8.0860(5)	4.0067(3)	6.2801(5)	203.46(3)	Ce	0.173(1)	¼	0.121(2)	3.76(1)
295	neut.	5.52	36(1)						Si	0.029(2)	¼	0.612(2)	2.494(9)
									D	0.379(1)	¼	0.422(2)	
2	D2b	4.32	*C*-$CeSiD_{0.96(1)}$	6.51	4.2414(2)	12.0765(4)	3.9927(2)	204.51(1)	Ce	0	0.3575(3)	¼	3.902(3)
295	neut.	5.52	64(1)						Si	0	0.0490(4)	¼	2.321(3)
									D	0	0.7607(3)	¾	
S13	D20	8.83	*P*-CeSiD	9.79	8.0946(7)	4.0095(3)	6.2791(5)	203.79(3)	Ce	0.177(1)	¼	0.126(2)	3.84(1)
353	neut.	2.76	29(1)						Si	0.02(2)	¼	0.615(2)	2.49(1)
									D	0.377(1)	¼	0.424(1)	

S13	D20	8.83	*C*-CeSiD	6.96	4.2439(2)	12.0966(5)	3.9956(2)	205.12(1)	Ce	0	0.3555(4)	¼	3.874(5)
353	neut.	2.76	71(2)						Si	0	0.0544(6)	¼	2.392(6)
									D	0	0.7576(3)	¾	
S8	D20	8.82	*P*-CeSiD	14.1	8.1124(7)	4.0158(3)	6.2870(6)	204.82(3)	Ce	0.177(1)	¼	0.131(2)	3.87(1)
475	neut.	2.79	31(1)						Si	0.019(2)	¼	0.614(2)	2.49(1)
									D	0.379(1)	¼	0.422(1)	
S8	D20	8.82	*C*-CeSiD	8.46	4.25140(2)	12.1213(6)	4.0040(2)	206.33(2)	Ce	0	0.3546(4)	¼	3.867(4)
475	neut.	2.79	69(2)						Si	0	0.0562(6)	¼	2.422(6)
									D	0	0.7566(4)	¾	
S2	G670	2.86	*P*-$CeSiH_{0.82}$	7.82	8.0821(4)	4.0049(2)	6.2837(3)	203.39(2)	Ce	0.1740(3)	¼	0.1238(3)	3.787(3)
295	X-ray	1.71	41.1(5)						Si	0.035(1)	¼	0.587(2)	2.348(8)
									H	0.379	¼	0.422	
S2	G670	2.86	*C*-$CeSiH_{0.96}$	7.45	4.2396(2)	12.0918(5)	3.9903(2)	204.56(1)	Ce	0	0.3548(1)	¼	3.860(1)
295	X-ray	1.71	58.9(6)						Si	0	0.0551(5)	¼	2.399(5)
									H	0	0.7607	¾	
Fig.	Device	R_{wp} / %	Phase	R_{Bragg} / %	*a* / Å	*b* / Å	*c* / Å	*V* / Å^3	At.	*x*	*y*	*z*	*d*(Ce–Ce) / Å
T/K	type	χ^2	content / %										*d*(Si–Si) / Å
